# Inserting “OFF-to-ON” BODIPY Tags into Cytokines: A Fluorogenic Interleukin IL-33 for Real-Time Imaging of Immune Cells

**DOI:** 10.1021/acscentsci.3c01125

**Published:** 2023-12-20

**Authors:** Abigail E. Reese, Fabio de Moliner, Lorena Mendive-Tapia, Sam Benson, Erkin Kuru, Thomas Bridge, Josh Richards, Jonathan Rittichier, Takanori Kitamura, Amit Sachdeva, Henry J. McSorley, Marc Vendrell

**Affiliations:** † Centre for Inflammation Research, The University of Edinburgh, EH16 4UU Edinburgh, United Kingdom; ‡ IRR Chemistry Hub, Institute for Regeneration and Repair, The University of Edinburgh, EH16 4UU, Edinburgh, United Kingdom; § Department of Genetics, Harvard Medical School, Boston, Massachusetts 02115, United States; ∥ Wyss Institute for Biologically Inspired Engineering, Harvard University, Boston, Massachusetts 02215, United States; ⊥ School of Chemistry, University of East Anglia, Norwich NR4 7TJ, United Kingdom; # Division of Cell Signaling and Immunology, School of Life Sciences, University of Dundee, Dundee DD1 4HN, United Kingdom; ¶ Centre for Reproductive Health, The University of Edinburgh, EH16 4UU Edinburgh, United Kingdom

## Abstract

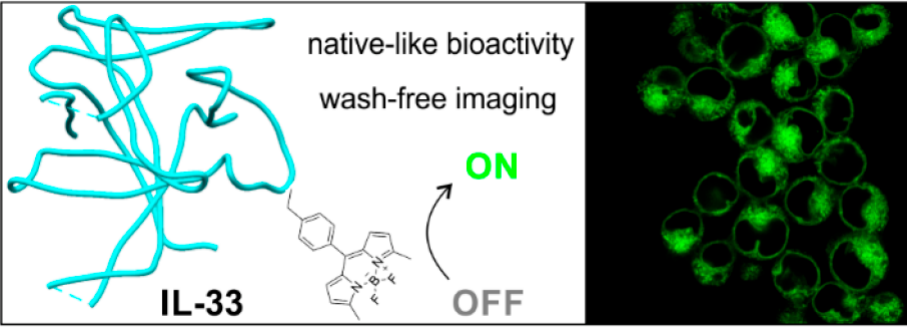

The essential functions that cytokine/immune cell interactions play in tissue homeostasis and during disease have prompted the molecular design of targeted fluorophores to monitor their activity in real time. Whereas activatable probes for imaging immune-related enzymes are common, many immunological functions are mediated by binding events between cytokines and their cognate receptors that are hard to monitor by live-cell imaging. A prime example is interleukin-33 (IL-33), a key cytokine in innate and adaptive immunity, whose interaction with the ST2 cell-surface receptor results in downstream signaling and activation of NF-κB and AP-1 pathways. In the present work, we have designed a chemical platform to site-specifically introduce OFF-to-ON BODIPY fluorophores into full cytokine proteins and generate the first nativelike fluorescent analogues of IL-33. Among different incorporation strategies, chemical aminoacylation followed by bioorthogonal derivatization led to the best labeling results. Importantly, the BODIPY-labeled IL-33 derivatives—unlike IL-33-GFP constructs—exhibited ST2-specific binding and downstream bioactivity profiles comparable to those of the wild-type interleukin. Real-time fluorescence microscopy assays under no wash conditions confirmed the internalization of IL-33 through ST2 receptors and its intracellular trafficking through the endosomal pathway. We envision that the modularity and versatility of our BODIPY labeling platform will facilitate the synthesis of minimally tagged fluorogenic cytokines as the next generation of imaging reagents for real-time visualization of signaling events in live immune cells.

## Abstract 2

We designed a platform to generate fluorogenic analogues of IL-33 by site-specifically introducing OFF-to-ON BODIPYs. Unlike IL-33-GFP, these constructs retain ST2 binding and downstream bioactivity.

## Introduction

Immune cells play essential functions in tissue homeostasis and during the progression of multiple pathological conditions, from inflammatory diseases to neurological disorders.^1−3^ These critical roles have prompted the design of imaging approaches to monitor their physiological patterns in live tissues in a noninvasive manner.^4,5^ One of the most conventional methods for imaging immune function involves the use of transgenic cells that express exogenous optical reporters (e.g., fluorescent proteins (FPs), bioluminescent proteins).^6,7^ While these approaches insert genetic reporters at specific loci for targeted cell imaging, the slow maturation of FPs hinders real-time measurements in cells, and their fluorescence readouts are not amenable to optical modulation (e.g., most FPs provide always-on emission signals). These limitations have encouraged the molecular design of activatable chemical probes for imaging subpopulations of immune cells in complex environments.^8−13^

To date, the majority of immune-targeted activatable fluorescent probes rely on enzyme-responsive structures.^14−16^ For example, our group and others have reported Förster resonance energy transfer (FRET) probes targeting cathepsins,^17,18^ neutrophil elastase,^19^ and granzymes^20−22^ to visualize macrophages, neutrophils, and T cells, respectively. However, many functions of immune cells are not associated with unique enzymatic reactivity profiles and instead are mediated by the specific binding of cytokines and chemokines to their cognate receptors (e.g., class I and II cytokine receptors, IL-1 family receptors, and G-protein coupled receptors) followed by downstream signaling.^23,24^ Our group has demonstrated that fluorescently labeled chemokines (e.g., chemokine ligand 2 or CCL2) can outperform antibodies for functional imaging of live cells^25,26^ and that C-terminal functionalization of CCL2 can produce analogues that (1) retain binding to the receptor (e.g., CCR2) and (2) enable imaging of disease-relevant subpopulations (e.g., tumor-associated macrophages). However, this approach is incompatible with chemical diversification at internal residues and requires relatively large amounts of the proteins for derivatization. In order to expand the toolbox and applications of fluorescent cytokines for immune cell signaling, we designed a generic platform combining synthetic biology and fluorophore chemistry to site-specifically incorporate small BODIPY tags into cytokine proteins.

Interleukin-33 (IL-33) is a cytokine from the IL-1 family and a fundamental component of innate and adaptive immunity.^27,28^ IL-33 is known as an alarmin cytokine because it is released on necrotic cell death, alerting the immune system to tissue damage.^29,30^ Upon release, IL-33 binds with high affinity to the ST2 receptor and recruits the IL-1 receptor accessory protein (IL-1RAcP)^31,32^ to promote downstream signaling in different immune cells, including mast cells, innate lymphoid cells, and regulatory T cells.^33^ The IL-33/ST2 axis modulates several inflammatory diseases such as asthma, atopic dermatitis, and inflammatory bowel diseases^34,35^ and is central to the response against helminth parasites.^36^ To avoid immune-mediated ejection, the intestinal nematode *Heligmosomoides polygyrus bakeri* secretes the HpARI protein,^30^ which binds and blocks IL-33, and HpBARI_Hom2, which binds and blocks ST2, allowing the parasite’s persistence in vivo.^28^ Despite the interest in the IL-33 pathway in a range of immune responses, there are limited tools to visualize real-time trafficking of the IL-33/ST2 complexes in live cells. We envisaged that IL-33 fluorescent analogues able to report binding to ST2 under wash-free imaging conditions would allow us to study cytokine internalization and trafficking in live cells.

BODIPY fluorophores are widely used scaffolds in biological imaging because of their neutral character, cell permeability, and favorable optical properties (e.g., high brightness and photostability). Our group has reported the rational design and synthesis of environmentally sensitive BODIPY amino acids^37,38^ and their integration into multiple peptides for targeted imaging;^39−41^ however, the adaptation of BODIPY fluorogens to protein derivatization has not been reported to date. In this work, we present the chemical synthesis of a collection of BODIPY fluorophores and fluorogens with suitable reactive groups (from reactive esters to charged tRNA moieties) and their characterization and evaluation for site-specific protein labeling. Furthermore, we have employed selected fluorophores to prepare the first BODIPY-tagged IL-33 analogues and used them for real-time imaging of IL-33/ST2 signaling in live cells. The modularity and versatility of this platform will accelerate the design of minimally tagged proteins with BODIPY fluorophores, including other cytokines.

## Results and Discussion

### Chemical Synthesis and Spectral Characterization of Reactive BODIPY Building Blocks

The most conventional strategy for introducing BODIPY tags into protein structures involves the direct conjugation of reactive fluorophores (e.g., *N*-hydroxysuccinimides, maleimides) to nucleophilic amino acids (e.g., lysines and cysteines, respectively).^42,43^ Despite being an effective approach to label proteins with BODIPY groups, these reactions are not site specific and yield heterogeneous mixtures with a diverse range of bioactivity profiles. The groups of Lee and Holland have recently reported photoactivatable fluorophores (e.g., aryl ketones and aryl azides, respectively) for light-controlled insertion of BODIPY moieties into proteins.^44,45^ These reactions are fast and proceed with reasonable yields, but their labeling efficiency is also highly dependent on the accessibility of reactive residues.

**Figure 1:**
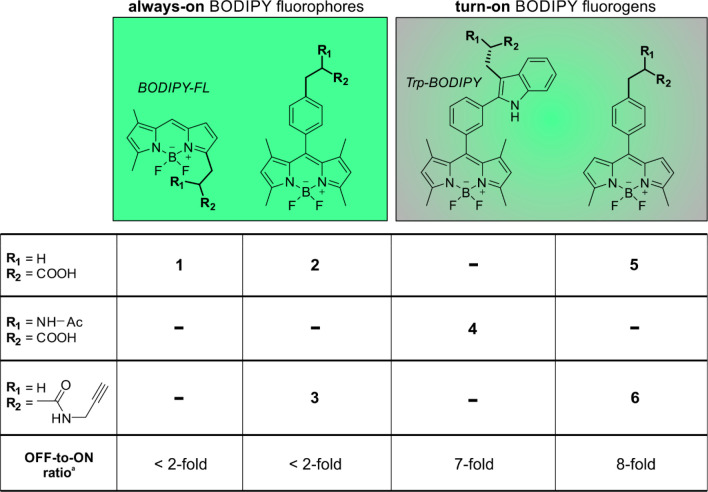
Chemical structures and fluorogenicity of BODIPY building blocks used in this study. For full synthetic details and characterization, see the Supporting Information. *^a^*Fluorogenicity determined by comparison of the fluorescence emission intensity of the compounds in phosphate buffer solution (PBS) with or without phosphatidylcholine liposomes (fluorescence emission plots in Figure S1).

Alternatively, genetic code reprogramming using the amber suppression technology provides a means to reassign the amber stop codon to an unnatural amino acid and introduce an exogenous chemical moiety (e.g., fluorophore) at a single, specific position of the protein structure.^46,47^ Some fluorescent amino acids (e.g., CouA, ANAP)^48−50^ have been incorporated into proteins using this methodology, but none have been applied to labeling cytokines, and very few examples considered the use of BODIPY fluorophores. For example, the group of Sisido first reported the incorporation of BODIPY-FL aminophenylalanine (aPhe) derivatives into large proteins (e.g., maltose-binding protein),^51,52^ and more recently, Alexandrov and co-workers described calmodulin analogues containing the BODIPY-FL dye.^53^ BODIPY-FL is a small-sized fluorophore, but it does not exhibit environmentally sensitive properties for wash-free imaging of signaling events. To overcome this shortcoming, we designed a collection of BODIPY-based fluorophores and fluorogens that could be adapted to protein labeling. In addition to the always-on BODIPY-FL (**1**, Figure 1) and the previously reported fluorogenic Trp-BODIPY amino acid (**4**, Figure 1), we synthesized tetramethyl (**2**–**3**, Figure 1) and dimethyl (**5**–**6**, Figure 1) BODIPY analogues including suitable groups for tRNA aminoacylation (e.g., carboxylic acids in compounds **2** and **5**) and bioorthogonal chemistry (e.g., propargyl groups in compounds **3** and **6**).

The synthesis of compounds **2**, **3**, **5**, and **6** was performed by adaptation of previously reported procedures^54,55^ (for full synthetic details and characterization, see the Supporting Information). Briefly, 2,4-dimethylpyrrole (for compounds **2**–**3**) or 2-methylpyrrole (for compounds **5**–**6**) was subjected to acid-catalyzed condensation with 4-formyl-phenylpropionic acid followed by oxidation of the dipyrromethane intermediates and insertion of the bridging boron atom by treatment with BF_3_·Et_2_O. Using this one-pot procedure, the BODIPY carboxylic acids **2** and **5** were isolated in overall moderate yields (i.e., 18% and 13%, respectively) and subsequently derivatized with *N*-propargylamine via amide bond formation using standard conditions (e.g., OxymaPure and *N*,*N*′-diisopropylcarbodiimide (DIC)) to yield the alkyne-containing compounds **3** and **6**, respectively (Figure S2).

Upon chemical synthesis, we analyzed the optical properties of the BODIPY building blocks (**1**–**6**) under the same experimental conditions. As expected, the BODIPY compounds showed similar excitation/emission wavelength maxima and extinction coefficients (e.g., 500/520 nm, Figure S3). In order to evaluate their suitability as OFF-to-ON fluorophores for the preparation of fluorogenic cytokines, we measured their brightness in aqueous media and in liposome suspensions mimicking hydrophobic microenvironments (e.g., plasma membrane) (Figure S1). In these experiments, we observed that compounds **1**–**3** based on BODIPY-FL or tetramethyl BODIPY scaffolds behaved as always-on fluorophores and displayed similar emission intensity in both environments, with BODIPY FL showing slightly higher brightness than the tetramethyl BODIPY dyes (Figure S1). On the other hand, compounds **4**–**6** displayed notable environmental sensitivity and enhanced fluorescence emission in hydrophobic media, proving their utility as fluorogenic building blocks. These results are in line with previous observations from our group and others,^56,57^ where the dimethyl BODIPY derivatives were found to have much higher turn on emission than their tetramethyl analogues. Previous TD-DFT computational studies from our group have also shown that environmental sensitivity in such systems is directly related to the crossing of a transition state (TS*) on the first excited state leading to nonradiative decay, with dimethyl BODIPY derivatives being more fluorogenic due to their smaller TS* barriers.^38^

### Chemical Activation of BODIPY Fluorophores as Precursors for Protein Labeling

Having synthesized a collection of BODIPY building blocks with variable optical properties, we next proceeded to their derivatization as precursors to enable site-specific incorporation into proteins. In order to use cell-free translation to site-specifically incorporate BODIPY fluorophores into proteins, we first needed to prepare tRNA acylated with the fluorophore. Among the different strategies described for the chemical acylation of tRNA, we first attempted flexizyme-based strategies reported by Suga and co-workers^58,59^ for the ligation of unnatural amino acids^60,61^ but with no successful results (Supplementary Discussion in the Supporting Information).

**Figure 2:**
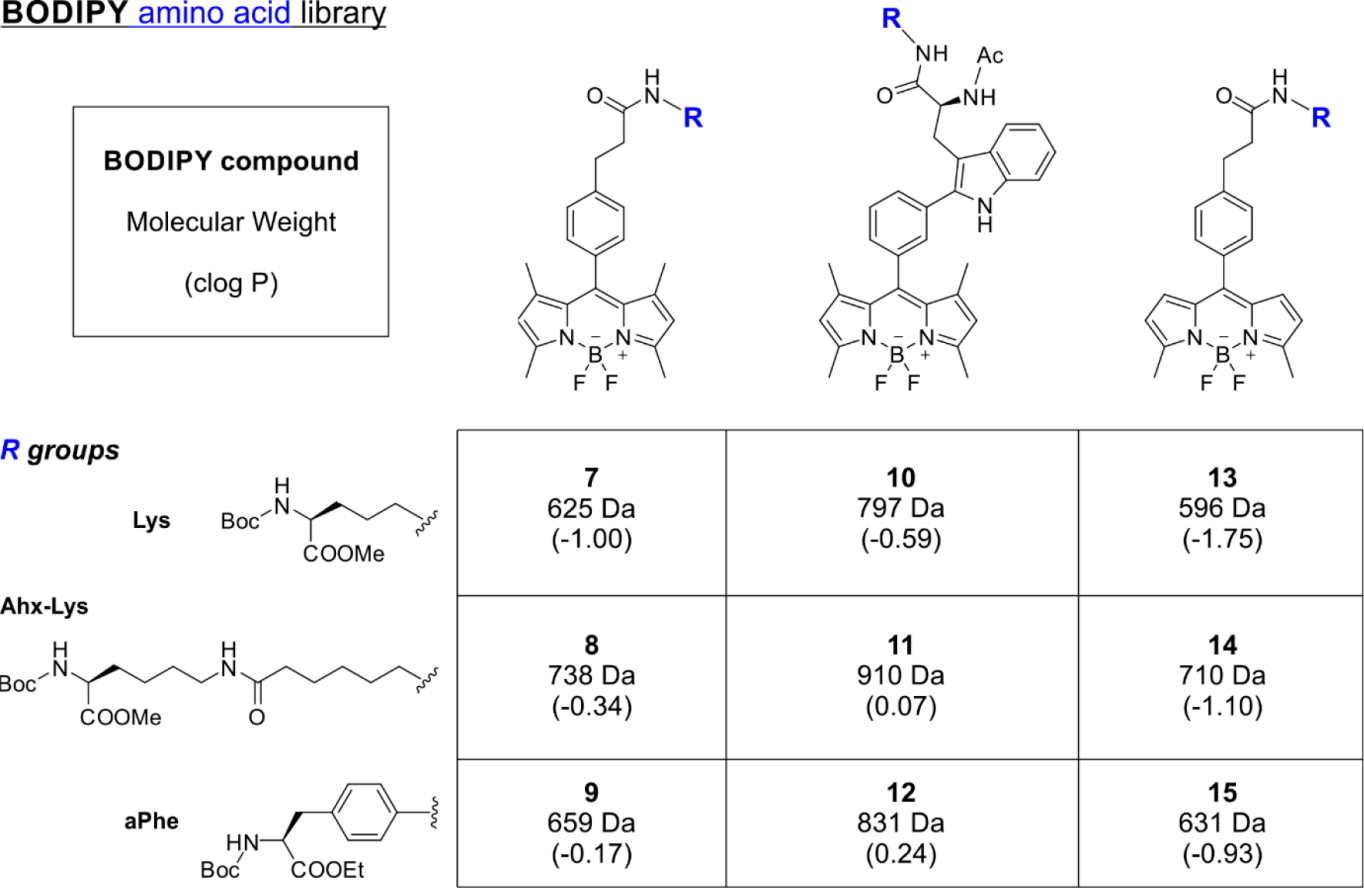
BODIPY amino acid library. Chemical structures and physicochemical properties of the BODIPY amino acids employed in this study.

In order to optimize the coupling of BODIPY tags to tRNA molecules, we decided to prepare reactive esters of BODIPY fluorophores that would be suitable for chemical aminoacylation. Briefly, this procedure involves the esterification of the 3′-hydroxyl group of 5′-phospho-2′-deoxyribocytidylylriboadenosine dinucleotide (pdCpA) followed by amine deprotection and enzymatic ligation to truncated tRNA molecules. The group of Sisido previously described the successful incorporation of BODIPY-FL (**1**, Figure 1) into streptavidin using tRNA aminoacylation;^52^ however, there are no reports with fluorogenic or environmentally sensitive BODIPY fluorophores. Therefore, we synthesized a collection of BODIPY derivatives with different spacers that resembled natural amino acids to minimize the potential steric hindrance between pdCpA and BODIPY fluorophores (Figure 2). Among the spacers, we selected aPhe, which has been reported for tRNA aminoacylation with other fluorophores, the natural amino acid l-lysine (Lys), and a longer spacer resulting from the coupling of Lys and aminohexanoic acid (Ahx).^52,62,63^ Whereas both aPhe and Lys are commercially available as Boc-protected precursors, the spacer Lys-Ahx was prepared by coupling of Boc-Lys-OMe and Fmoc-Ahx-OH followed by Fmoc deprotection with diethylamine (Figure S4).

We synthesized a total of nine BODIPY amino acids (**7**–**15**, Figure 2), where each of the fluorophores **2**, **4**, and **5** (Figure 1) was coupled to the three different spacers (i.e., aPhe, Lys, Lys-Ahx) using solution-phase chemistry. Lys and Lys-Ahx reacted with all fluorophores under standard OxymaPure and DIC conditions; however, the poorly nucleophilic aPhe required the coupling agent PyOxim in the presence of triethylamine to render the corresponding BODIPY adducts (compounds **9**, **12** , and **15**, Figure 2). All nine compounds were purified as methyl or ethyl esters with variable yields (e.g., from ∼20% compound **9** to ∼80% for compound **12**; full synthetic details and characterization are given in the Supporting Information) and hydrolyzed to the corresponding carboxylic acids with a diluted solution of NaOH immediately prior to pdCpA esterification. Importantly, this library featured a diverse collection of BODIPY amino acids as potential building blocks for tRNA derivatization, including both fluorogenic and always-on fluorophores of different sizes (e.g., molecular weights from 625 to 910 Da) and variable clog P values (e.g., in decreasing order for aPhe > Lys-Ahx > Lys, from 0.24 for compound **12** to −1.75 for compound **13**).

### BODIPY Amino Acids Can Be Effectively Charged to tRNA

**Figure 3:**
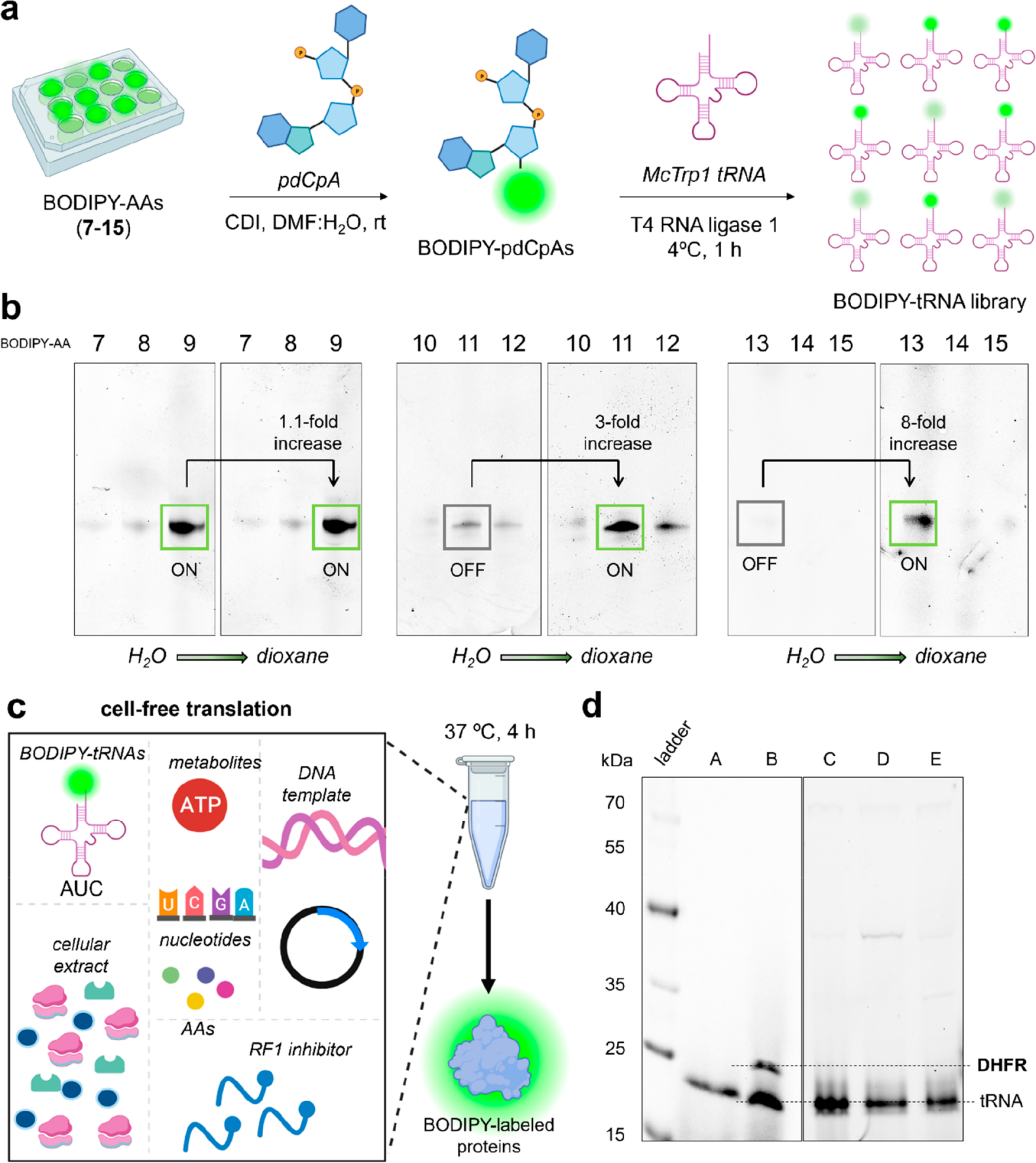
tRNA charging with BODIPY amino acids and in vitro translation to a model protein. (a) Schematic illustration of the chemical aminoacylation of the BODIPY amino acids (**7**–**15**) to truncated tRNA species to render a BODIPY-tagged tRNA library. (b) Representative TBE-urea gels showing the efficiency of aminoacylation by in-gel fluorescence analysis (λ_exc_: 473 nm) in water and dioxane. Fluorescence fold increases were measured by densitometry analysis. (c) Components of the cell-free extract utilized to produce BODIPY-tagged proteins. (d) Representative SDS-PAGE gel displaying the presence and absence of fluorescent bands corresponding to BODIPY-labeled DHFR proteins. A: negative control with no plasmid DNA; B: DHFR-**1**; C: DHFR-**9**; D: DHFR-**11**; E: DHFR-**13**.

After completing the synthesis of Boc-protected BODIPY amino acids with suitable groups for tRNA acylation, we optimized a procedure to remove Boc protecting groups from the amino terminal end of the BODIPY amino acids that would retain some integrity of the fluorophore, given its lability to acid media.^64,65^ This step is important because BODIPY amino acids ligated to tRNA molecules must contain a free amino group for their site-specific incorporation into proteins. After testing several deprotection conditions, we found that the treatment of Boc-protected BODIPY amino acids with a solution of 5:95 (trifluoroacetic acid (TFA):acetonitrile (ACN)) at 0 °C for 10 min showed the lowest extent of degradation (Figure S5) and proceeded to synthesize a library of fluorescent pdCpA conjugates including our BODIPY amino acids to identify the most suitable spacer for every fluorophore. For these experiments, we employed a standard chemical aminoacylation approach whereby the BODIPY amino acids (**7**–**15**, Figure 2) were hydrolyzed and activated using 1,1′-carbonyldiimidazole (CDI) and then reacted with pdCpA dinucleotide in a mixture of DMF:H_2_O (Figure S6). Recently, the group of Kool has reported imidazole-based reagents for selective RNA acylation.^66,67^ Unlike the pdCpA approach, these reagents can acylate tRNA directly; however, their application to protein labeling is not straightforward because the amino acids must be incorporated using a two-step labeling approach (e.g., click chemistry), and a guiding “inducer” DNA strand is needed for site-specific derivatization. Subsequently, all BODIPY-pdCpA conjugates were treated with TFA:ACN (5:95) and enzymatically ligated to McTrp1^62^ (i.e., a suppressor tRNA to decode UAG) by incubating with T4 RNA ligase 1 for 1 h at 4 °C. In order to compare the extent of bioconjugation for the different BODIPY amino acids, TBE-urea gels were scanned for their fluorescence emission after excitation at 500 nm (Figure 3). Furthermore, we assessed whether the charged tRNA molecules retained the always-on or fluorogenic properties of the native BODIPY amino acids by comparing the intensity of the fluorescent bands in aqueous media and in dioxane, given that the latter favors the turn-on effect in environmentally sensitive BODIPY conjugates but not in always-on fluorophores.^38^ The results of our analysis highlighted that tetramethyl and dimethyl BODIPY dyes were best ligated to tRNA when using aPhe and Lys spacers, respectively, whereas Trp-BODIPY was efficiently incorporated into tRNA when the Lys-Ahx spacer was employed (Figure 3 and Figure S7), which highlights the increased bulkiness of the latter fluorophore. Moreover, the gel analysis confirmed that the BODIPY-charged tRNAs retained the optical properties of their precursor amino acids; namely, tRNAs containing the environmentally sensitive BODIPY fluorophores **11** and **13** showed threefold and eight fold fluorescence increase in dioxane. On the other hand, tRNAs labeled with the always-on BODIPY fluorophore **9** showed minimal differences in fluorescence emission when comparing the two different microenvironments (Figure 3).

Having prepared several BODIPY-charged tRNA molecules, we next assessed their capacity for ribosomal integration by attempting their incorporation at the N-terminal end (e.g., position 2, where steric hindrance is marginal) of the model protein dihydrofolate reductase (DHFR).^68^ For this, we used site-directed mutagenesis to introduce an UAG codon at the position 2 of DHFR and included the release factor 1 inhibitor Api137^69^ to ensure full reassignment of UAG (Figure 3). Under these conditions, none of the three BODIPY amino acids (**9**, **11**, and **13**, Figure 2) were incorporated into the model protein to a large extent, showing limited compatibility as substrates for ribosome-mediated peptide bond formation (Figure 3). As a positive control, we employed the previously reported amino acid aPhe-BODIPY FL resulting from the coupling of aPhe and BODIPY-FL^63^ (Figure S8), which could be effectively incorporated into DHFR under the same experimental conditions (Figure 3). Altogether, these results indicate that (1) BODIPY fluorophores can be efficiently ligated to tRNA using chemical aminoacylation, (2) screening and selection of amino acid spacers are needed to efficiently ligate different BODIPY fluorophores to tRNA constructs, and (3) fluorophores larger than BODIPY-FL might not be readily accepted by the ribosomal machinery and are incompatible with cell-free in vitro translation upon tRNA aminoacylation.

### Site-Specific Incorporation of BODIPY Fluorogens into Proteins Using Bioorthogonal Ligation

**Figure 4:**
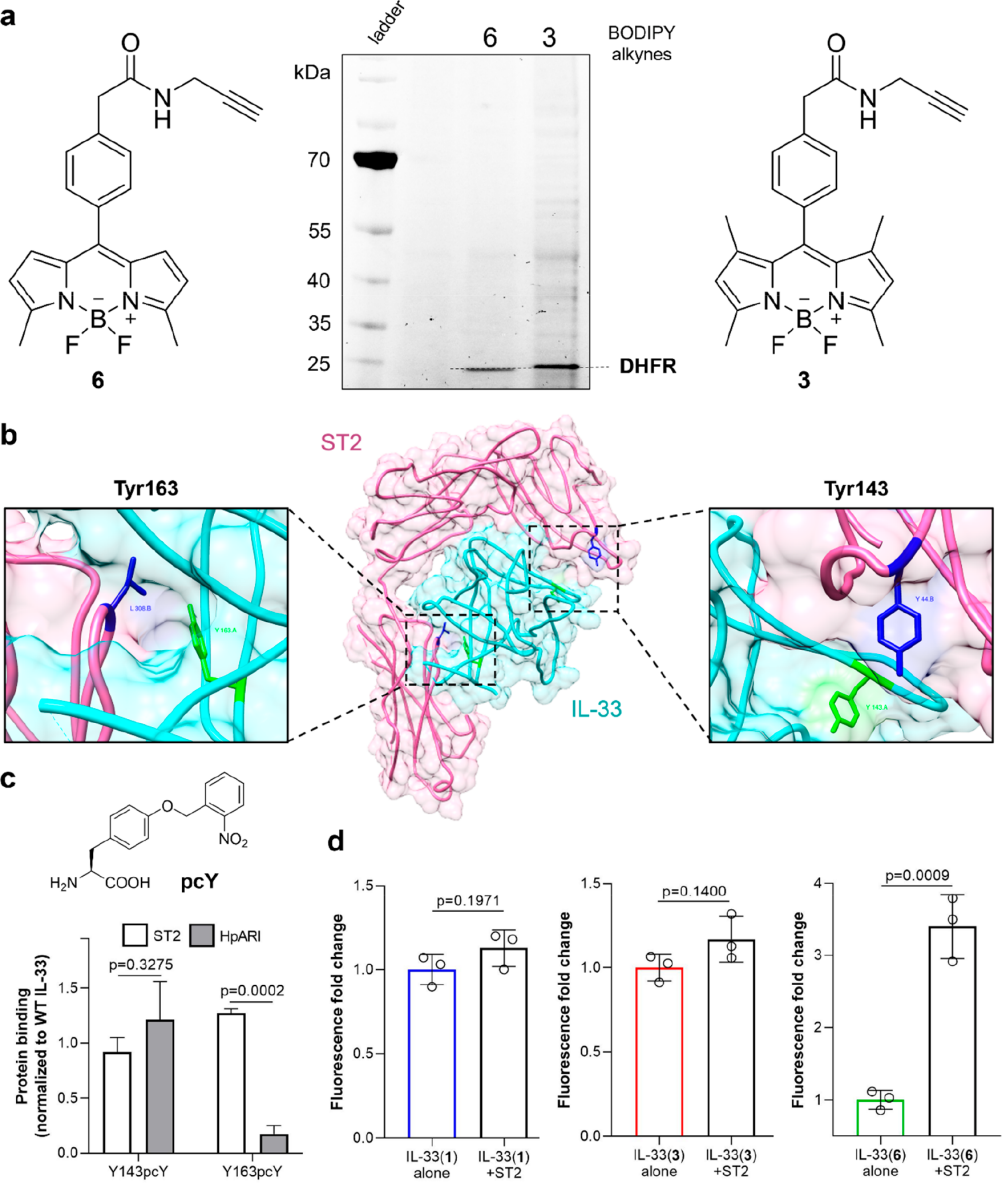
Site-specific incorporation of BODIPY fluorophores into the proteins DHFR and interleukin IL-33. (a) Chemical structures of BODIPY-alkynes **3** and **6** and representative SDS-PAGE gel displaying fluorescent bands corresponding to BODIPY-labeled DHFR proteins (λ_exc_: 473 nm). (b) Illustration created using Chimera of the 3D structure of the complex formed by IL-33 (cyan) and its receptor ST2 (pink) (PDB: 4KC3). The illustration highlights the residues Tyr143 and Tyr163 (both in green) at the binding interface and their proximity to ST2 residues (Leu306 and Tyr144, both in dark blue). (c) Chemical structure of pcY. ELISA binding assays of IL-33 Y143pcY and IL-33 Y163pcY to ST2 or HpARI. Values normalized to the binding of wild-type IL-33 and presented as means ± SEM (*n* = 3). *P* values obtained from two-tailed *t* tests. (d) Fluorescence emission of IL-33(**1**), IL-33(**3**), and IL-33(**6**) (50 μg mL^–1^) before and after incubation with the receptor ST2 (200 μg mL^–1^). Values presented as means ± SD (*n* = 3). *P* values obtained from two-tailed *t* tests.

In order to introduce BODIPY fluorogens into protein structures, we next evaluated the bioorthogonal coupling between alkyne-modified BODIPY fluorophores and suitably functionalized proteins. For this, we decided to assess copper-catalyzed azide–alkyne cycloaddition (CuAAC)^70^ as this strategy is compatible with the site-specific incorporation of azidoPhe, an unnatural amino acid that has been previously reported for protein derivatization.^71,72^ With this in mind, we first optimized the bioorthogonal coupling between the alkyne-modified BODIPY compound **3** (Figure 4) and azidoPhe. Briefly, we tested two water-soluble reducing agents (i.e., sodium ascorbate and tris(2-carboxyethyl)phosphine (TCEP)), two chelating agents (i.e., tris(hydroxypropyl-triazolylmethyl)amine (THPTA) and 2-[4-{(bis[(1-*tert*-butyltriazolyl)methyl]amino)methyl}-triazolyl]acetic acid (BTTAA)) and different ratios of compound **3** vs azidoPhe. Among these reaction conditions, we found that the sodium ascorbate and THPTA were the most suitable reducing and chelating agents respectively, alongside a 10-fold excess of compound **3** over azidoPhe (Figures S9 and S10). Finally, we applied these reaction conditions to conjugate the BODIPY fluorophores **3** and **6** to the model protein DHFR. First, we used chemical aminoacylation to charge azidoPhe to McTrp1 tRNA via pdCpA ligation (Figure S11), which was followed by the successful in vitro protein expression and CuAAC-mediated conjugation of the fluorophores **3** and **6** to DHFR (Figure 4).

Having optimized the site-specific incorporation of the BODIPY fluorophores **3** and **6** into a model protein, we next decided to employ this protocol to prepare new bioactive analogues of IL-33. One major limitation of using fused FPs for fluorescently labeling small cytokines such as IL-33 is that the resulting constructs can exhibit impaired activity when compared to the native cytokines. To address this point, we compared endogenous IL-33 to IL-33-GFP in a cytokine-release assay (Figure S12) and found that IL-33-GFP had no measurable activity unlike the endogenous interleukin. These results indicate that a C-terminal fusion of GFP to IL-33 impairs the biological activity of the cytokine.

Therefore, in order to rationalize the derivatization of IL-33 with the BODIPY fluorophores **3** and **6** and identify optimal labeling sites within the protein sequence, we examined the structure of the IL-33/ST2 binary complex and selected two hydrophobic residues (Tyr143 and Tyr163, Figure 4) at the binding interface between IL-33 and its receptor ST2. Notably, these residues are reported not to be involved in essential interactions between IL-33 and ST2,^31^ but they protrude across the binding interface to result in microenvironmental changes that could activate BODIPY fluorogens upon receptor binding. We analyzed the effect of introducing bulky groups at the positions 143 and 163 of IL-33 by preparing two mutants where we replaced the native Tyr residues with the reported photocaged Tyr (pcY, Figure 4),^73^ which can be readily introduced into proteins in *Escherichia coli* by means of the specific aaRS/suppressor tRNA *Methanocaldococcus jannaschi* pair (*Mj*pcYRS/ *Mj*tRNA_CUA_).^74^ For these experiments, the *Mj*pcYRS/*Mj*tRNA_CUA_ pair was cloned into a pULTRA plasmid and transformed into BL21(DE3)pLysS cells with a pSANG10 plasmid encoding the IL-33 protein. The IL-33 coding sequences included an in-frame amber stop codons at positions 143 or 163 for pcY incorporation and His_6_ tag for purification. The two proteins (IL-33 Y143pcY and IL-33 Y163pcY) were extracted from the periplasm, purified, and characterized by gel electrophoresis to confirm site-specific incorporation of pcY (Figure S13).

We compared the binding affinity of IL-33 Y143pcY and IL-33 Y163pcY against ST2 and HpARI^28^ by ELISA assays. Both mutants exhibited comparable affinities for ST2 when compared to the wild-type IL-33 (Figure 4); however, IL-33 Y163pcY displayed significantly reduced binding to the inhibitory protein HpARI, suggesting the potential interference of bulky moieties in close proximity to the Tyr163 residue. In view of the similar molecular recognition properties between IL-33 Y143pcY and wild-type IL-33, we selected the position 143 as a preferred site for nonperturbative incorporation of BODIPY fluorophores. Next, we employed the pSANG10-IL-33 plasmid with the in-frame amber stop codon at position 143 to site specifically introduce the BODIPY fluorophores **3** and **6** into IL-33 (i.e., first via azidoPhe incorporation and then CuAAC-mediated conjugation followed by purification). As a positive control, we also synthesized the fluorescent IL-33 analogue whereby we directly introduced the aPhe-BODIPY FL amino acid using cell-free translation. All three IL-33 analogues (e.g., IL-33(**1**) for aPhe-BODIPY FL, IL-33(**3**), and IL-33(**6**)) were characterized by in-gel fluorescence and Western blot analysis (Figure S14). For reaction volumes around 250 μL, final protein yields ranged from 12 to 18 μg mL^–1^ for BODIPY-labeled IL-33 constructs while yields were close to 50 μg mL^–1^ for unlabeled IL-33 (Figure S15). We also compared the fluorescence response of all IL-33 mutants before and after binding to their receptor ST2. For these experiments, we measured the fluorescence emission of IL-33 analogues (50 μg mL^–1^) before and after incubation with ST2 (200 μg mL^–1^) and observed a significant turn-on effect in IL-33(**6**) upon receptor binding (∼fourfold increase) whereas IL-33(**1**) and IL-33(**3**) displayed nonsignificant changes in their emission intensity (Figure 4). These results confirmed that the always-on behavior for fluorophores **1** and **3** and the fluorogenic behavior for fluorophore **6** (i.e., turn-on emission upon ST2 binding) were retained after incorporation into the IL-33 structure and highlight IL-33(**1**), IL-33(**3**), and IL-33(**6**) as the first reported fluorescent and fluorogenic analogues of IL-33 with nativelike recognition properties. Finally, we scaled up the production of IL-33(**3**) and IL-33(**6**) in *E. coli*, whereby we first transformed BL21(pLysS) cells with a pULTRA-CNF plasmid containing a *Mj*RS(CNF))/*Mj*tRNA pair and the pSANG10-IL33-143TAG plasmid. The protein IL-33 Y143azidoPhe was extracted from the periplasm and purified to obtain 1.6 mg from protein expression in 1 litre culture. IL-33(**3**) and IL-33(**6**) were isolated after conjugation to the BODIPY fluorophores **3** and **6**, respectively, and characterized by Coomassie staining and in-gel fluorescence analysis (Figure S16).

### Fluorogenic IL-33 Mutants Retain Nativelike Bioactivity Profiles and Can Be Employed as Wash-free Imaging Agents in Live Cells

**Figure 5:**
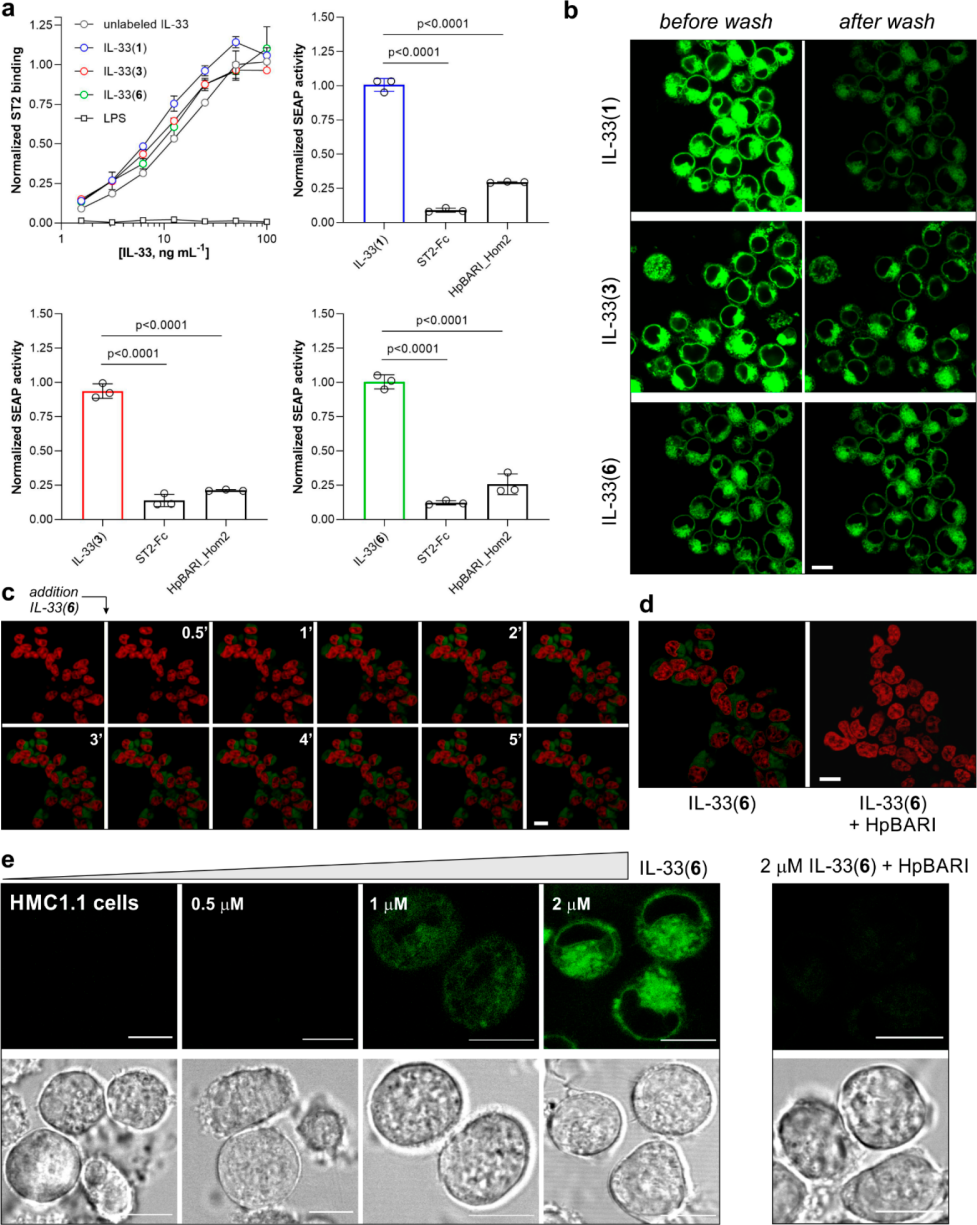
IL-33 derivatives retain nativelike binding properties and are compatible with wash-free fluorescence microscopy. (a) Dose-dependent SEAP response of IL-33 HEK-Blue reporter cells to increasing concentrations of unlabeled IL-33, IL-33(**1**), IL-33(**3**), and IL-33(**6**). Normalized responses of unlabeled IL-33, IL-33(**1**), IL-33(**3**), and IL-33(**6**) in the presence or absence of IL-33 and ST2 inhibitors (both at 10 μg mL^–1^). Values normalized to the binding of wild-type IL-33 and presented as means ± SD (*n* = 3). *P* values obtained by one-way ANOVA with multiple comparisons. (b) Representative fluorescence confocal microscopy images of transfected HEK-Blue cells after incubation with IL-33(**1**), IL-33(**3**), or IL-33(**6**) (all in green) before and after washing (once with 100 μL of PBS). Scale bar: 10 μm. (c) Time-course fluorescence confocal microscopy images of ST2-expressing cells after incubation with IL-33(**6**) green) and nuclear counterstain DRAQ5 (red) (full movie in Supplementary Movie 1). Scale bar: 10 μm. Excitation lasers: 488 nm (for IL-33 analogues), 561 nm (for DRAQ5). (d) Fluorescence confocal microscopy images of ST2-expressing cells after incubation with IL-33(**6**) (green) and nuclear counterstain DRAQ5 (red) with and without HpBARI (1 μg mL^–1^) (full movie in Supplementary Movie 2). Scale bar: 8 μm. (e) Representative bright-field and fluorescence confocal microscopy images of HMC1.1 human mast cells after incubation with increasing concentrations of IL-33(**6**) (green) before and after blocking with HpBARI (1 μg mL^–1^). Scale bar: 10 μm. Excitation lasers: 488 nm (for IL-33 analogues), 561 nm (for DRAQ5).

Having synthesized IL-33(**1**), IL-33(**3**), and IL-33(**6**) as derivatives of IL-33, we examined their bioactivity profiles using the IL-33 HEK-Blue reporter cell line, where IL-33 binding and downstream signaling through activation of the NF-κB and AP-1 pathways can be readily monitored by the secretion of embryonic alkaline phosphatase (SEAP).^75^ Briefly, we compared the biological activity of unlabeled IL-33 and the three fluorescent derivatives IL-33(**1**), IL-33(**3**), and IL-33(**6**) at different concentrations within the 1.5–100 ng mL^–1^ range. Lipopolysaccharide (LPS) was employed as a negative control because it may be present in proteins generated with the cell-free translation system and to verify that, unlike IL-33, the stimulation of Toll-like receptors 4 (TLR-4) did not increase SEAP activity through a MyD88-dependent response. As shown in Figure 5, all derivatives retained the ability to cause downstream signaling to a similar extent as unlabeled IL-33. Next, we confirmed that the observed signals were due to the specific recognition between IL-33 and ST2 receptors by incubating the cells with ST2-Fc as an IL-33 inhibitor and HpBARI_Hom2 as an ST2 inhibitor. Under all these conditions, we observed almost full reduction of SEAP activity (Figure 5), confirming that the signals from IL-33(**1**), IL-33(**3**), and IL-33(**6**) were produced in response to an active interleukin.

After confirming the suitability of IL-33(**1**), IL-33(**3**), and IL-33(**6**) as fluorescent analogues of IL-33, we decided to evaluate their application as imaging agents of ST2-mediated internalization. The group of Zhao recently reported that IL-33 can induce the internalization of ST2 receptors; however, there is very limited information on the intracellular trafficking and dynamics of IL-33/ST2 complexes in live cells.^76,77^ To run these experiments, we plated HEK-Blue reporter cells expressing ST2 receptors and incubated them separately with IL-33(**1**), IL-33(**3**), and IL-33(**6**) for 5 min before imaging them using fluorescence confocal microscopy. Given the variable optical behavior of the different proteins (e.g., IL-33(**1**) and IL-33(**3**) contained always-on fluorophores whereas IL-33(**6**) contained a turn-on fluorophore), we also compared their fluorescence signals before and after washing. As suggested from the results of the functional assays which indicated ST2-mediated signaling, we observed that all three IL-33 analogues were internalized into cells. Furthermore, we observed that IL-33(**1**) and IL-33(**3**) showed reduced fluorescence signals after washing once with PBS, which may indicate nonspecific cell internalization. On the contrary, IL-33(**6**) may represent a more accurate reporter of ST2-mediated internalization as it showed similar fluorescence intensities in cells before and after washing, dose-dependent intracellular staining (Figure S17), and lack of fluorescence signals in nontransfected HEK293 cells (Figure S17). High-magnification fluorescence microscopy images showed subcellular localization in lysosomal compartments (Figure S18) and partial colocalization with Lysotracker Red (Figure S19), suggesting internalization through the endosomal pathway.

Given the fluorogenic character of IL-33(**6**), we employed this protein to perform a time-course analysis of the rate of ST2-mediated internalization by wash-free fluorescence confocal microscopy. Reporter cells were counterstained with the nuclear counterstain DRAQ5 and monitored by time-lapse imaging (Figure 5). Our results showed that ST2-mediated internalization could be observed after minutes, and we also confirmed that the fluorescence signals from IL-33(**6**) inside cells were exclusively due to the interaction with ST2 receptors, as proven by blockade experiments with the ST2 antagonist HpBARI_Hom2, which left cells completely devoid of green fluorescence signals (Figure 5). Finally, we performed experiments in live HMC1.1 human mast cells as a representative immune cell line that can be stimulated by IL-33.^78^ Specifically, we titrated IL-33(**6**) using fluorescence microscopy and observed ST2-specific and dose-dependent fluorescence staining of the cells, which was blocked by competition with HpBARI_Hom2 (Figure 5). We demonstrated that the labeling of HMC1.1 cells with IL-33(**6**) could be also monitored by flow cytometry, proving the compatibility of our fluorogenic analogues with different fluorescence modalities (Figure S20). Altogether, these results demonstrate that IL-33(**1**), IL-33(**3**), and IL-33(**6**) retain the binding affinity of unlabeled IL-33 for their native receptors and that IL-33(**6**) can be used as a fluorogenic reporter to image IL-33/ST2 signaling events in real time.

## Conclusions

In summary, we have optimized a novel chemical platform to introduce BODIPY fluorophores into small cytokines and generated the first fluorescent analogues of the interleukin IL-33 with nativelike bioactivity profiles. We have synthesized a collection of 15 BODIPY fluorophores with variable physicochemical and optical properties, including chemically diverse linkers and functionalized amino acid groups. We demonstrated that BODIPY fluorophores can be efficiently charged to tRNA using chemical aminoacylation—but not flexizymes—and optimized the linkers to efficiently ligate different BODIPY fluorophores to tRNA constructs. Our experiments indicate that fluorophores larger than BODIPY-FL may not be readily accepted by the ribosomal machinery; however, they are compatible with chemical aminoacylation followed by bioorthogonal derivatization, as demonstrated by the incorporation of the BODIPY fluorophores **3** and **6** into the model protein DHFR and the interleukin IL-33. Using a combination of structural analysis and protein engineering, we optimized the fluorophore incorporation site within the IL-33 sequence (i.e., position 143) for enhanced turn-on fluorogenic response and minimal disruption of biological activity. Subsequently, we synthesized three labeled IL-33 analogues—all with BODIPY tags at position 143—with always-on fluorescence emission (i.e., probes IL-33(**1**) and IL-33(**3**)) or turn-on behavior (i.e., probe IL-33(**6**)). Unlike IL-33-GFP constructs, the three IL-33 derivatives exhibited ST2-specific binding and downstream activity comparable to the wild-type IL-33, thus corroborating the suitability of our chemical approach for minimally invasive tagging of cytokines. Finally, we have employed the fluorogenic probe IL-33(**6**) for no wash, real-time fluorescence microscopy in live cells expressing ST2 receptors and confirmed the selective receptor-mediated internalization of IL-33 and its intracellular trafficking through the endosomal pathway. The versatility and modularity of this BODIPY labeling platform will accelerate the design of future fluorescent cytokine-based biosensors for immunological studies.

## Supporting Information Available

Detailed synthetic procedures and full chemical characterization data, supplementary spectroscopic and imaging data, and supplementary discussion (PDF)
Supplementary movie of time-lapse fluorescence confocal microscopy images of transfected HEK-Blue cells after incubation with IL-33(6) (green) and nuclear counterstain DRAQ5 (red) (AVI)
Supplementary movie of time-lapse fluorescence confocal microscopy images of transfected HEK-Blue cells that had been pre-incubated with HpBARI (1 μg mL^–1^) for 1 h (AVI)
Transparent Peer Review report available (PDF)

## Supplementary Material

oc3c01125_si_001.pdf

oc3c01125_si_003.avi

oc3c01125_si_004.avi
