# Supplementary material for: Inserting “OFF-to-ON” BODIPY Tags into Cytokines: A Fluorogenic Interleukin IL-33 for Real-Time Imaging of Immune Cells
Source: ACS Cent Sci. 2023 Dec 20;10(1):143–54. doi: 10.1021/acscentsci.3c01125 (PMC10823590; doi:10.1021/acscentsci.3c01125)
Supplement: Supplementary file 1 — oc3c01125_si_001.pdf [file oc3c01125_si_001.pdf]

Electronic Supporting Information

Inserting 'OFF-to-ON' BODIPY tags into cytokines: a fluorogenic interleukin IL-33 for real-time imaging of immune cells

Abigail E Reese,[1,2] Fabio de Moliner,[1,2] Lorena Mendive-Tapia,[1,2] Sam Benson,[1,2] Erkin Kuru,[3,4] Thomas Bridge,[5] Josh Richards,[6] Jonathan Rittichier,[3] Takanori Kitamura,[7] Amit Sachdeva,[5] Henry J McSorley,[6] Marc Vendrell[1,2,*]

[1] Centre for Inflammation Research, The University of Edinburgh, EH16 4UU, Edinburgh, UK.

[2] IRR Chemistry Hub, Institute for Regeneration and Repair, The University of Edinburgh, EH16 4UU Edinburgh, UK.

[3] Department of Genetics, Harvard Medical School, Boston, USA.

[4] Wyss Institute for Biologically Inspired Engineering, Harvard University, Boston, USA.

[5] School of Chemistry, University of East Anglia, Norwich, UK.

[6] Division of Cell Signaling and Immunology, School of Life Sciences, University of Dundee, Dundee, UK.

[7] Centre for Reproductive Health, The University of Edinburgh, EH16 4UU, Edinburgh, UK.

Corresponding email address: marc.vendrell@ed.ac.uk.

Number of pages: 71

Number of figures: 25

Electronic Supporting Information

Table of contents

1. Materials and methods
2. Experimental procedures
3. Supplementary figures
4. Supplementary movies
5. Supplementary discussion
6. NMR spectra
7. References

Materials and methods

Commercially available reagents were used without further purification. The BODIPY fluorophores BODIPY FL (compound **1** in manuscript) and Fmoc-Trp(BODIPY)-OH (precursor of compound **4** in manuscript) were purchased from Thermo Fisher Scientific and Sigma-Aldrich, respectively. Thin layer chromatography was conducted on Merck silica gel 60 F254 sheets and visualized by UV (254 and 365 nm). Silica gel (particle size 35–70 µm) was used for column chromatography. ^{1}H and ^{13}C spectra were recorded in a Bruker Avance 500 spectrometer (at 500 and 125 MHz, respectively). Data for ^{1}H NMR spectra are reported as chemical shift δ (ppm), multiplicity, coupling constant (Hz) and integration. Data for ^{13}C NMR spectra reported as chemical shifts relative to the solvent peak. HPLC-MS analysis was performed on a Waters Alliance 2695 separation module connected to a Waters PDA2996 photodiode array detector and a ZQ Micromass mass spectrometer (ESI-MS) with a Phenomenex® column (C_{18}, 5 µm, 4.6 × 150 mm). HRMS (ESI positive) were obtained with a Bruker ESI Micro-TOF mass spectrometer.

Experimental procedures

Chemical synthesis.

10-(4-(2-Carboxyethyl)phenyl)-5,5-difluoro-1,3,7,9-tetramethyl-5*H*-dipyrrolo[1,2-*c*:2',1'-*f*][1,3,2]diazaborinin-4-ium-5-uide (2)

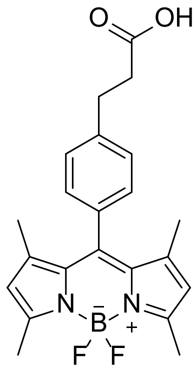

3-(4-Formylphenyl)propionic acid (200 mg, 1.1 mmol, 1 eq) and 2,4-dimethylpyrrole (240 mg, 2.5 mmol, 2.2 eq) were dissolved in DCM (30 mL) under N_2 atmosphere. TFA (1 drop) in DCM (5 mL) was then added via a syringe and the reaction was allowed to stir overnight at r.t. in the dark. Then, DDQ (254 mg, 1.1 mmol, 1 eq) in DCM (15 mL) was added, followed after 15 minutes by Et_3N (2.3 mL, 25.2 mmol, 22.5 eq) and $BF_3 \cdot Et_2O$ (2.3 mL, 16.8 mmol, 15 eq). After 1 h, the reaction was stopped, diluted with DCM (50 mL) and washed with saturated $NaHCO_3$ (2 × 100 mL). The organic layer was dried over $MgSO_4$ and concentrated to dryness under reduced pressure. The crude was purified by column chromatography (DCM:MeOH, 95:5) to give compound **2** as a dark red solid (70 mg, 18% yield).

^{1}H NMR (500 MHz, $CDCl_3$) δ 7.35 (d, *J* = 8.2 Hz, 2H), 7.23 (d, *J* = 8.2 Hz, 2H), 5.99 (s, 2H), 3.07 (t, *J* = 7.6 Hz, 2H), 2.75 (t, *J* = 7.6 Hz, 2H), 2.57 (s, 6H), 1.39 (s, 6H).

^{13}C NMR (125 MHz, $CDCl_3$) δ 177.4, 155.4, 143.1, 141.6, 141.2, 133.1, 131.5, 129.0, 128.2, 121.2, 35.5, 30.5, 14.4, 14.3.

HRMS (m/z, ESI): calcd for $C_{22}H_{24}BF_2N_2O_2^+$ $[M+H]^+$: 397.1893, found: 397.1898.

5,5-Difluoro-1,3,7,9-tetramethyl-10-(4-(3-oxo-3-(prop-2-yn-1-ylamino)propyl)phenyl)-5*H*-dipyrrolo[1,2-*c*:2',1'-*f*][1,3,2]diazaborinin-4-ium-5-uide (3)

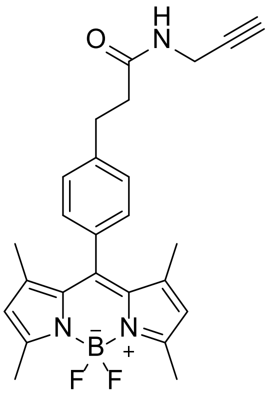

To a mixture of **2** (25 mg, 0.06 mmol, 1 eq), DIC (11 mg, 0.09 mmol, 1.5 eq) and OxymaPure (13 mg, 0.09 mmol, 1.5 eq) in DCM (1 mL) was added propargylamine (8 μL, 0.1 mmol, 2 eq), and reaction was stirred at r.t. for 5 h. Upon reaction completion, the solvent was removed under reduced pressure and the crude was purified by column chromatography (DCM:MeOH, 99.5:0.5) to give compound **3** as a red solid (15 mg, 58% yield).

^{1}H NMR (500 MHz, $CDCl_3$) δ 7.34 (d, *J* = 8.2 Hz, 2H), 7.21 (d, *J* = 8.1 Hz, 2H), 5.99 (s, 2H), 5.54 (s, 1H), 4.04 (dd, *J* = 5.2, 2.6 Hz, 2H), 3.08 (t, *J* = 7.6 Hz, 2H), 2.72 – 2.44 (m, 2H), 2.57 (s, 6H), 2.24 (t, *J* = 2.6 Hz, 1H), 1.39 (s, 6H).

^{13}C NMR (125 MHz, $CDCl_3$) δ 171.2, 155.4, 143.0, 141.71, 141.69, 133.0, 131.5, 129.1, 128.2, 121.2, 79.3, 71.8, 38.1, 31.3, 29.2, 14.6, 14.4.

HRMS (m/z, ESI): calcd for $C_{25}H_{26}BF_2N_3ONa^+$ $[M+Na]^+$: 456.2029, found: 456.2029.

(*R*)-10-(3-(3-(2-acetamido-2-carboxyethyl)-1*H*-indol-2-yl)phenyl)-5,5-difluoro-1,3,7,9-tetramethyl-5*H*-dipyrrolo[1,2-*c*:2',1'-*f*][1,3,2]diazaborinin-4-ium-5-uide (4)

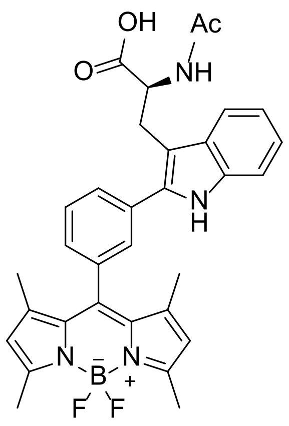

Fmoc-Trp(BODIPY)-OH (90 mg, 0.13 mmol, 1 eq) was dissolved in 20% diethylamine in DCM (5 mL) and allowed to stir at r.t. for 30 min. Solvents were removed under reduced pressure and the residue was triturated in cold Et_2O to give a red solid that was recovered by filtration and redissolved in MeCN (8 mL). Acetyl chloride (14 μL, 0.14 mmol, 1.1 eq) and Et_3N (28 μL, 0.14 mmol, 1.1 eq) were added and reaction was stirred at r.t. for 2 h. Then, the solvent was removed under reduced pressure and the crude was purified by column chromatography (DCM:MeOH, 95:5 → 85:15) to give compound **4** as a red solid (53 mg, 72% yield).

^{1}H NMR (500 MHz, $CDCl_3$) δ 8.21 (s, 1H), 7.79 – 7.66 (m, 3H), 7.58 (t, *J* = 7.7 Hz, 1H), 7.52 (t, *J* = 1.5 Hz, 1H), 7.50 – 7.45 (m, 1H), 7.42 – 7.37 (m, 2H), 7.35 – 7.32 (m, 1H), 7.28 – 7.23 (m, 1H), 7.20 – 7.15 (m, 1H), 6.00 (s, 1H), 5.97 (s, 1H), 5.19 (d, *J* = 7.9 Hz, 1H), 4.65 (dd, *J* = 13.4, 6.9 Hz, 1H), 4.31 – 4.22 (m, 2H), 4.12 – 4.08 (m, 1H), 3.59 – 3.38 (m, 2H), 2.57 (s, 3H), 2.56 (s, 3H), 1.48 (s, 3H), 1.45 (s, 3H).

^{13}C NMR (125 MHz, $CDCl_3$) δ 174.6, 155.9, 143.7, 143.0, 141.3, 140.5, 136.1, 135.9, 134.9, 131.3, 130.0, 128.6, 127.8, 127.7, 127.0, 125.0, 123.2, 121.4, 120.5, 120.00, 119.1, 111.1, 107.9, 67.1, 54.4, 47.0, 14.6.

HRMS (m/z, ESI): calcd for $C_{32}H_{31}BF_2N_4O_3Na^+$ $[M+Na]^+$: 591.2349, found: 591.2351.

10-(4-(2-Carboxyethyl)phenyl)-5,5-difluoro-3,7-dimethyl-5*H*-dipyrrolo[1,2-*c*:2',1'-*f*][1,3,2]diazaborinin-4-ium-5-uide (5)

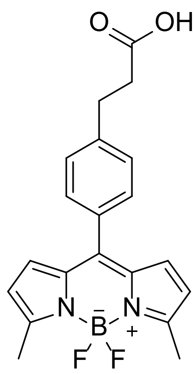

3-(4-Formylphenyl)propionic acid (200 mg, 1.1 mmol, 1 eq) and 2-methylpyrrole (205 mg, 2.5 mmol, 2.25 eq) were dissolved in DCM (30 mL) under N_2 atmosphere. TFA (1 drop) in DCM (5 mL) was then added via a syringe and the reaction was allowed to stir overnight at r.t. in the dark. Then, DDQ (254 mg, 1.1 mmol, 1 eq) in DCM (15 mL) was added, followed after 15 minutes by Et_3N (2.3 mL, 25.2 mmol, 22.5 eq) and $BF_3 \cdot Et_2O$ (2.3 mL, 16.8 mmol, 15 eq). After 1 h, the reaction was stopped, diluted with DCM (50 mL) and washed with saturated $NaHCO_3$ (2 × 100 mL). The organic layer was dried over $MgSO_4$ and concentrated to dryness under reduced pressure. The crude was purified by column chromatography (DCM:MeOH, 95:5 → 9:1) to give compound **5** as a red solid (55 mg, 13% yield).

^{1}H NMR (500 MHz, $CDCl_3$) δ 7.45 (d, *J* = 8.0 Hz, 2H), 7.35 (d, *J* = 8.0 Hz, 2H), 6.74 (d, *J* = 4.1 Hz, 2H), 6.28 (d, *J* = 4.1 Hz, 2H), 3.09 (t, *J* = 7.7 Hz, 2H), 2.79 (t, *J* = 7.7 Hz, 2H), 2.67 (s, 6H).

^{13}C NMR (125 MHz, $CDCl_3$) δ 177.5, 157.5, 142.4, 134.5, 132.3, 130.7, 130.4, 128.2, 119.4, 35.0, 30.3, 14.9.

HRMS (m/z, ESI): calcd for $C_{20}H_{20}BF_2N_2O_2^+$ $[M+H]^+$: 369.1580, found: 369.1577.

5,5-Difluoro-3,7-dimethyl-10-(4-(3-oxo-3-(prop-2-yn-1-ylamino)propyl)phenyl)-5*H*-dipyrrolo[1,2-*c*:2',1'-*f*][1,3,2]diazaborinin-4-ium-5-uide (6)

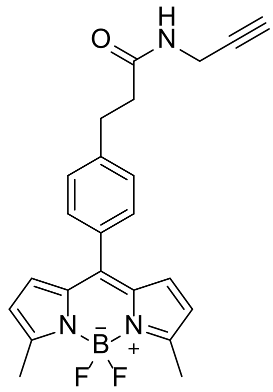

To a mixture of **5** (25 mg, 0.06 mmol, 1 eq), DIC (13 mg, 0.09 mmol, 1.5 eq) and OxymaPure (14 mg, 0.09 mmol, 1.5 eq) in DCM (1 mL) was added propargylamine (9 μL, 0.1 mmol, 2 eq), and reaction was stirred at r.t. for 5 h. Upon completion, the solvent was removed under reduced pressure and the crude was purified by column chromatography (DCM:MeOH, 99:1 → 98:2) to give compound **6** as a red solid (6 mg, 25% yield).

^{1}H NMR (500 MHz, $CDCl_3$) δ 7.45 (d, J = 8.3 Hz, 2H), 7.33 (d, J = 8.3 Hz, 2H), 6.74 (d, J = 4.1 Hz, 2H), 6.28 (d, J = 4.1 Hz, 2H), 5.58 (s, 1H), 4.09 (dd, J = 5.2, 2.6 Hz, 2H), 3.10 (t, J = 7.6 Hz, 2H), 2.67 (s, 6H), 2.58 (t, J = 7.6 Hz, 2H), 2.24 (t, J = 2.6 Hz, 1H).

^{13}C NMR (125 MHz, $CDCl_3$) δ 171.2, 157.5, 142.9, 142.5, 134.5, 132.2, 130.7, 130.4, 128.2, 119.29, 79.4, 71.7, 37.7, 31.2, 29.3, 14.9.

HRMS (m/z, ESI): calcd for $C_{23}H_{23}BF_2N_3O^+$ $[M+H]^+$: 406.1896, found: 406.1890.

(*S*)-10-(4-(3-((5-((*Tert*-butoxycarbonyl)amino)-6-methoxy-6-oxohexyl)amino)-3-oxopropyl)phenyl)-5,5-difluoro-1,3,7,9-tetramethyl-5*H*-dipyrrolo[1,2-*c*:2',1'-*f*][1,3,2]diazaborinin-4-ium-5-uide (7)

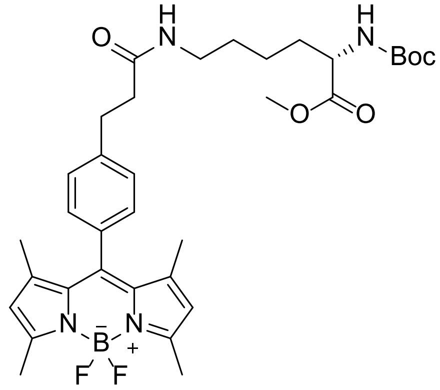

To a mixture of **2** (25 mg, 0.06 mmol, 1 eq), DIC (13 mg, 0.09 mmol, 1.5 eq) and OxymaPure (14 mg, 0.09 mmol, 1.5 eq) in DMF (2 mL) was added Boc-Lys-OMe (25 mg, 0.09 mmol, 1.5 eq), and reaction was stirred at r.t. overnight. Then, the solvent was removed under reduced pressure and the crude was purified by column chromatography (Hex:EtOAc, 3:7 → 1:9) to give compound **7** as an orange solid (18 mg, 45% yield).

^{1}H NMR (500 MHz, $CDCl_3$) δ 7.34 (d, J = 8.1 Hz, 2H), 7.20 (d, J = 8.1 Hz, 2H), 5.99 (s, 2H), 5.72 (s, 1H), 5.13 (s, 1H), 4.42 (q, J = 7.1 Hz, 1H), 4.29 (d, J = 4.3 Hz, 1H), 3.82 (dd, J = 12.9, 6.5 Hz, 1H), 3.76 (s, 3H), 3.24 (dt, J = 13.9, 7.1 Hz, 2H), 3.06 (t, J = 7.7 Hz, 2H), 2.56 (d, J = 6.3 Hz, 6H), 2.53 (d, J = 7.4 Hz, 2H), 1.85 – 1.79 (m, 1H), 1.70 – 1.62 (m, 1H), 1.57 – 1.49 (m, 2H), 1.45 (s, 9H), 1.18 (s, 3H), 1.17 (s, 3H).

^{13}C NMR (125 MHz, $CDCl_3$) δ 173.2, 172.1, 158.7, 155.6, 155.4, 143.0, 142.0, 141.8, 132.9, 131.5, 129.1, 128.1, 125.8, 121.2, 108.3, 80.0, 63.0, 53.1, 52.4, 42.5, 39.2, 38.4, 32.6, 31.5, 28.8, 28.3, 23.4, 22.6, 14.6, 14.4, 14.1.

HRMS (m/z, ESI): calcd for $C_{34}H_{45}BF_2N_4O_5Na^+$ $[M+Na]^+$: 661.3343, found: 661.3346.

(*S*)-5,5-Difluoro-10-(4-(6-(methoxycarbonyl)-2,2-dimethyl-4,12,19-trioxo-3-oxa-5,11,18-triazahenicosan-21-yl)phenyl)-1,3,7,9-tetramethyl-5*H*-dipyrrolo[1,2-*c*:2',1'-*f*][1,3,2]diazaborinin-4-ium-5-uide (8)

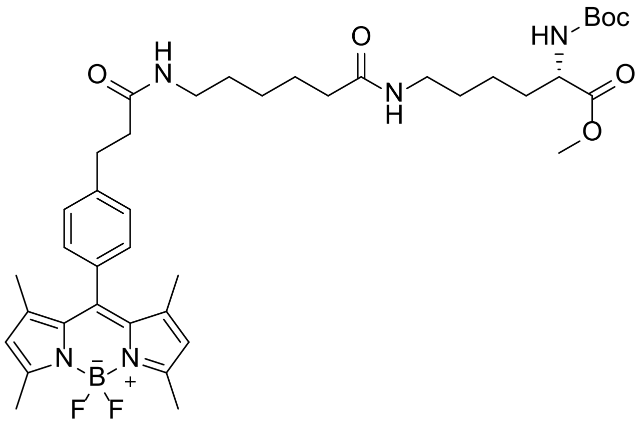

To a mixture of **2** (25 mg, 0.06 mmol, 1 eq), DIC (13 mg, 0.09 mmol, 1.5 eq) and OxymaPure (14 mg, 0.09 mmol, 1.5 eq) in DMF (2 mL) was added Boc-Lys(Ahx)-OMe (34 mg, 0.09 mmol, 1.5 eq), and the reaction was stirred at r.t. overnight. Then, the solvent was removed under reduced pressure and the crude was purified by column chromatography (EtOAc → DCM:MeOH, 95:5) to give compound **8** as an orange solid (13 mg, 29% yield).

^{1}H NMR (500 MHz, $CDCl_3$) δ 7.34 (d, *J* = 8.1 Hz, 2H), 7.20 (d, *J* = 8.1 Hz, 2H), 5.99 (s, 2H), 5.82 (s, 2H), 5.15 (s, 1H), 4.32 – 4.26 (d, *J* = 4.4 Hz, 1H), 3.75 (s, 3H), 3.30 – 3.21 (m, 4H), 3.07 (t, *J* = 7.6 Hz, 2H), 2.60 – 2.49 (m, 2H), 2.57 (s, 6H), 2.20 (t, *J* = 7.3 Hz, 2H), 1.86 – 1.77 (m, 2H), 1.73 – 1.61 (m, 4H), 1.59 – 1.47 (m, 4H), 1.46 (s, 9H), 1.43 – 1.30 (m, 3H), 1.38 (s, 6H).

^{13}C NMR (125 MHz, $CDCl_3$) δ 173.2, 171.9, 162.6, 155.4, 143.0, 142.1, 141.8, 132.8, 131.5, 129.1, 128.1, 121.2, 80.0, 53.1, 52.3, 39.2, 39.1, 38.3, 36.5, 36.1, 32.5, 31.5, 29.7, 29.1, 28.9, 28.3, 26.3, 24.9, 22.6, 14.6, 14.4.

HRMS (m/z, ESI): calcd for $C_{40}H_{56}BF_2N_5O_6Na^+$ $[M+Na]^+$: 774.4183, found 774.4193.

(*S*)-10-(4-(3-((4-(2-((*Tert*-butoxycarbonyl)amino)-3-ethoxy-3-oxopropyl)phenyl)amino)-3-oxopropyl)phenyl)-5,5-difluoro-1,3,7,9-tetramethyl-5*H*-dipyrrolo[1,2-*c*:2',1'-*f*][1,3,2]diazaborinin-4-ium-5-uide (9)

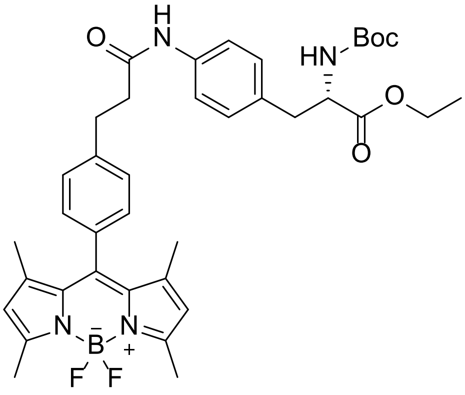

A mixture of **2** (25 mg, 0.06 mmol, 1 eq), PyOxim (36 mg, 0.07 mmol, 1.1 eq) and Boc-aPhe-OEt (20 mg, 0.06 mmol, 1 eq) in DMF (2 mL) was stirred at r.t. for 2 h in the presence of DIPEA (16 µL, 0.09 mmol, 1.5 eq). Then, the solvent was removed under reduced pressure and the crude was purified by column chromatography (Hex:EtOAc, 6:4) to give compound **9** as an orange solid (8 mg, 19% yield).

^{1}H NMR (500 MHz, $CDCl_3$) δ 7.43 – 7.33 (m, 4H), 7.20 (d, *J* = 8.1 Hz, 2H), 7.17 (s, 1H), 7.09 (d, *J* = 8.4 Hz, 2H), 5.98 (s, 2H), 4.99 (s, 1H), 4.57 – 4.50 (m, 1H), 4.18 (q, *J* = 7.1 Hz, 2H), 3.15 (t, *J* = 7.5 Hz, 2H), 3.12 – 2.99 (m, 2H), 2.71 (t, *J* = 7.5 Hz, 2H), 2.56 (s, 6H), 1.44 (s, 9H), 1.33 (s, 6H), 1.26 (t, *J* = 7.1 Hz, 3H).

^{13}C NMR (125 MHz, $CDCl_3$) δ 171.8, 169.8, 155.4, 155.1, 143.1, 141.7, 136.6, 133.0, 132.2, 131.5, 130.6, 129.9, 129.2, 128.2, 121.2, 119.8, 79.9, 61.4, 54.5, 39.3, 37.7, 31.3, 29.7, 28.3, 14.6, 14.3, 14.2.

HRMS (m/z, ESI): calcd for $C_{38}H_{45}BF_2N_4O_5Na^+$ $[M+Na]^+$: 709.3343, found 709.3334.

10-(3-(3-((*S*)-2-Acetamido-3-(((*S*)-5-((*tert*-butoxycarbonyl)amino)-6-methoxy-6-oxohexyl)amino)-3-oxopropyl)-1*H*-indol-2-yl)phenyl)-5,5-difluoro-1,3,7,9-tetramethyl-5*H*-dipyrrolo[1,2-*c*:2',1'-*f*][1,3,2]diazaborinin-4-ium-5-uide (10)

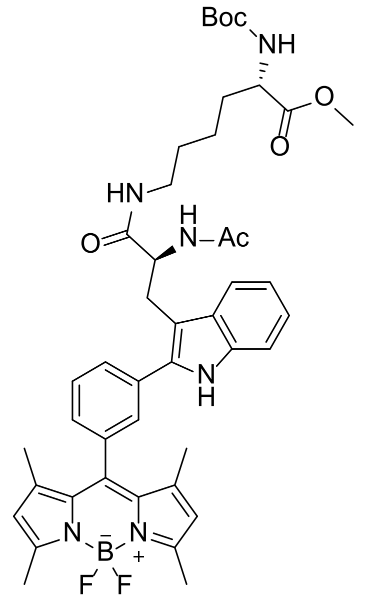

To a mixture of compound **4** (10 mg, 0.02 mmol, 1 eq), DIC (5 mg, 0.03 mmol, 1.5 eq) and OxymaPure (4 mg, 0.03 mmol, 1.5 eq) in DMF (1 mL) was added Boc-Lys-OMe (7 mg, 0.03 mmol, 1.5 eq), and the reaction was stirred at r.t. overnight. Then, the solvent was removed under reduced pressure and the crude was purified by column chromatography (Hex:EtOAc, 1:1 → EtOAc) to give compound **10** as an orange solid (8 mg, 58% yield).

^{1}H NMR (500 MHz, $CDCl_3$) δ 9.11 (s, 1H), 7.88 (t, *J* = 8.6 Hz, 2H), 7.68 (t, *J* = 7.7 Hz, 1H), 7.53 (s, 1H), 7.40 (d, *J* = 7.9 Hz, 1H), 7.33 (d, *J* = 7.7 Hz, 1H), 7.25 (t, *J* = 7.2 Hz, 1H), 7.20 (t, *J* = 7.4 Hz, 1H), 6.51 (s, 1H), 6.01 (s, 2H), 5.17 (br, s, 1H), 5.05 – 4.98 (m, 1H), 4.93 – 4.84 (m, 1H), 4.17 – 3.98 (m, 1H), 3.68 (s, 3H), 3.46 (dd, *J* = 14.0, 5.1 Hz, 1H), 3.32 – 3.18 (m, 1H), 3.08 (br, s, 1H), 2.82 – 2.75 (m, 1H), 2.58 (s, 6H), 2.04 (s, 3H), 1.97 – 1.88 (m, 1H), 1.46 (s, 15H), 1.37 – 1.24 (m, 4H), 0.98 – 0.81 (m, 4H).

^{13}C NMR (125 MHz, $CDCl_3$) δ 173.1, 172.9, 170.9, 169.8, 155.8, 155.4, 143.0, 143.0, 140.8, 136.1, 135.9, 134.7, 133.6, 131.4, 131.3, 130.0, 128.8, 128.6, 127.4, 123.2,

123.1, 121.4, 121.3, 120.5, 119.5, 111.2, 108.6, 80.4, 60.4, 53.6, 53.1, 52.3, 38.9, 32.7, 29.7, 29.0, 28.3, 28.2, 23.2, 22.7, 22.2, 21.1, 14.6, 14.5.

HRMS (m/z, ESI): calcd for $C_{44}H_{54}BF_2N_6O_6^+$ [M+H]$^+$: 811.4160, found 811.4154.

10-(3-(3-((6*S*,20*S*)-20-Acetamido-6-(methoxycarbonyl)-2,2-dimethyl-4,12,19-trioxo-3-oxa-5,11,18-triazahenicosan-21-yl)-1*H*-indol-2-yl)phenyl)-5,5-difluoro-1,3,7,9-tetramethyl-5*H*-dipyrrolo[1,2-*c*:2',1'-*f*][1,3,2]diazaborinin-4-ium-5-uide (11)

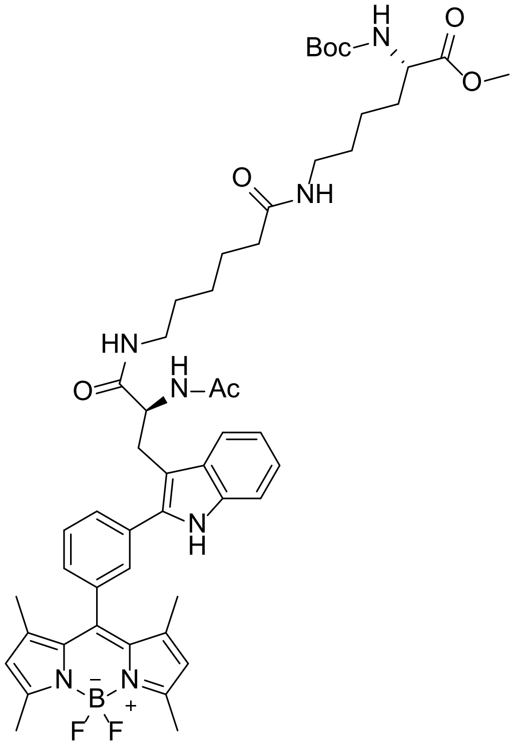

To a mixture of compound **4** (20 mg, 0.03 mmol, 1 eq), DIC (8 mg, 0.05 mmol, 1.5 eq) and OxymaPure (8 mg, 0.05 mmol, 1.5 eq) in DMF (1.5 mL) was added Boc-Lys(Ahx)-OMe (38 mg, 0.10 mmol, 3 eq), and the reaction was stirred at r.t. for 6 h. Then, the solvent was removed under reduced pressure and the crude was purified by column chromatography (EtOAc → DCM:MeOH, 95:5) to give compound **11** as an orange solid (12 mg, 38% yield).

^{1}H NMR (500 MHz, $CDCl_3$) δ 9.42 (s, 1H), 7.84 (dd, *J* = 19.0, 7.9 Hz, 2H), 7.65 (t, *J* = 7.7 Hz, 1H), 7.56 (t, *J* = 1.5 Hz, 1H), 7.40 (d, *J* = 8.0 Hz, 1H), 7.35 – 7.28 (m, 1H), 7.26 – 7.19 (m, 1H), 7.16 (t, *J* = 7.5 Hz, 1H), 6.74 (br, d, *J* = 7.5 Hz, 1H), 6.02 (s, 1H), 6.01 (s, 1H), 5.89 (br, s, 1H), 5.44 (s, 1H), 5.17 (br, d, *J* = 8.0 Hz, 1H), 4.84 (ddd, *J* = 10.8, 7.8, 5.1 Hz, 1H), 4.38 (q, *J* = 7.1 Hz, 1H), 4.32 – 4.24 (m, 1H), 4.14 (q, *J* = 7.1 Hz, 1H),

3.74 (s, 3H), 3.46 (dd, J = 14.0, 4.9 Hz, 1H), 3.37 – 3.09 (m, 3H), 3.01 – 2.95 (m, 1H), 2.89 – 2.73 (m, 1H), 2.57 (s, 6H), 2.03 (s, 3H), 2.01 – 1.93 (m, 1H), 1.86 – 1.56 (m, 2H), 1.49 (s, 3H), 1.48 (s, 3H), 1.45 (s, 9H), 1.38 (t, J = 7.1 Hz, 3H), 1.28 (t, J = 7.1 Hz, 3H), 1.07 – 0.93 (m, 2H), 0.91 – 0.74 (m, 2H).

^{13}C NMR (125 MHz, $CDCl_3$) δ 173.9, 173.9, 173.3, 171.3, 170.9, 170.4, 162.8, 158.7, 155.7, 143.2, 143.1, 141.0, 136.2, 135.7, 134.6, 133.7, 131.4, 130.0, 128.6, 128.6, 127.3, 127.1, 125.9, 123.0, 121.4, 121.3, 120.2, 119.5, 111.4, 108.3, 108.2, 80.1, 63.1, 60.5, 53.7, 53.2, 52.4, 39.2, 36.2, 32.5, 29.0, 28.5, 28.3, 26.1, 25.3, 23.1, 22.6, 21.1, 14.6, 14.0.

HRMS (m/z, ESI): calcd for $C_{50}H_{65}BF_2N_7O_7^+$ $[M+H]^+$: 924.5001, found 924.4990.

10-(3-(3-((*S*)-2-Acetamido-3-((4-((*S*)-2-((*tert*-butoxycarbonyl)amino)-3-ethoxy-3-oxopropyl)phenyl)amino)-3-oxopropyl)-1*H*-indol-2-yl)phenyl)-5,5-difluoro-1,3,7,9-tetramethyl-5*H*-dipyrrolo[1,2-*c*:2',1'-*f*][1,3,2]diazaborinin-4-ium-5-uide (12)

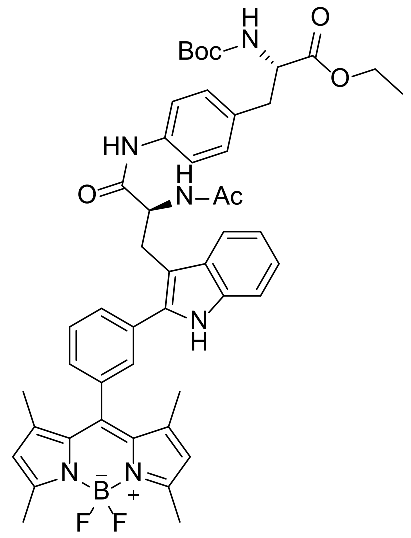

A mixture of compound **4** (15 mg, 0.03 mmol, 1 eq), PyOxim (15 mg, 0.03 mmol, 1.1 eq) and Boc-aPhe-OEt (8 mg, 0.03 mmol, 1 eq) in DMF (2 mL) was stirred at r.t. for 2 h in the presence of DIPEA (11 μL, 0.06 mmol, 2 eq). Then, the solvent was removed under reduced pressure and the crude was purified by column chromatography (Hex:EtOAc, 6:4) to give **12** as an orange solid (8 mg, 85% yield).

^{1}H NMR (500 MHz, MeOD) δ 7.85 (d, *J* = 7.9 Hz, 1H), 7.76 (t, *J* = 7.1 Hz, 1H), 7.71 (d, *J* = 7.9 Hz, 1H), 7.67 – 7.62 (m, 1H), 7.60 – 7.55 (m, 1H), 7.52 (dd, *J* = 7.1, 4.6 Hz, 1H), 7.41 – 7.21 (m, 4H), 7.15 – 7.07 (m, 2H), 7.06 – 6.98 (m, 3H), 6.76 (d, *J* = 8.4 Hz, 2H), 6.02 (d, *J* = 16.5 Hz, 1H), 4.74 (dd, *J* = 8.0, 5.1 Hz, 1H), 4.56 – 4.23 (m, 1H), 4.20 – 4.02 (m, 3H), 3.54 (dd, *J* = 14.6, 6.1 Hz, 1H), 3.39 – 3.33 (m, 2H), 3.21 – 3.11 (m, 1H), 2.98 (dd, *J* = 13.8, 6.0 Hz, 1H), 2.84 (dd, *J* = 13.8, 8.4 Hz, 1H), 2.49 (s, 3H), 2.46 (s, 3H), 1.92 (s, 3H), 1.52 (s, 3H), 1.41 (s, 9H), 1.22 (t, *J* = 7.1 Hz, 3H).

^{13}C NMR (125 MHz, MeOD) δ 173.9, 172.6, 171.7, 156.7, 156.4, 155.3, 143.8, 143.40, 141.6, 141.1, 136.6, 136.5, 135.5, 134.6, 131.2, 129.7, 129.6, 129.1, 128.8, 127.9, 127.5, 127.4, 127.3, 126.9, 126.7, 124.94, 124.88, 122.9, 121.7, 121.0, 120.9, 119.4, 119.0, 118.7, 118.4, 117.8, 116.3, 110.8, 110.7, 109.7, 108.0, 79.2, 66.6, 60.8, 55.5, 55.1, 53.4, 36.6, 27.3, 27.1, 21.0, 13.6, 13.5, 13.2, 13.0.

HRMS (m/z, ESI): calcd for $C_{48}H_{54}BF_2N_6O_6^+$ $[M+H]^+$: 859.4088, found 859.4090.

(*S*)-10-(4-(3-((5-((*Tert*-butoxycarbonyl)amino)-6-methoxy-6-oxohexyl)amino)-3-oxopropyl)phenyl)-5,5-difluoro-3,7-dimethyl-5*H*-dipyrrolo[1,2-*c*:2',1'-*f*][1,3,2]diazaborinin-4-ium-5-uide (13)

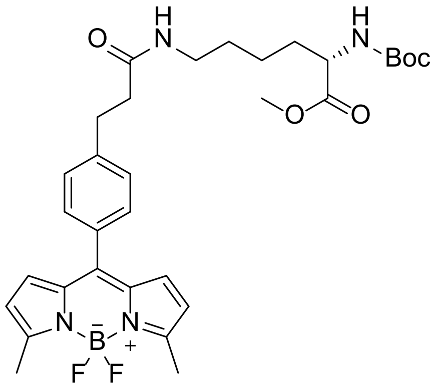

To a mixture of **5** (25 mg, 0.07 mmol, 1 eq), DIC (13 mg, 0.1 mmol, 1.5 eq) and OxymaPure (14 mg, 0.10 mmol, 1.5 eq) in DMF (2 mL) was added Boc-Lys-OMe (26 mg, 0.10 mmol, 1.5 eq), and the reaction was stirred at r.t. overnight. Then, the solvent was removed under reduced pressure and the crude was purified by column chromatography (Hex:EtOAc, 3:7 → 1:9) to give compound **13** as an orange solid (25 mg, 61% yield).

^{1}H NMR (500 MHz, $CDCl_3$) δ 7.43 (d, *J* = 8.1 Hz, 2H), 7.33 (d, *J* = 8.1 Hz, 2H), 6.73 (d, *J* = 4.1 Hz, 2H), 6.28 (d, *J* = 4.1 Hz, 2H), 5.71 (br, s, 1H), 5.13 br, (d, *J* = 7.8 Hz, 1H), 4.43 – 4.14 (m, 1H), 3.75 (s, 3H), 3.27 (dt, *J* = 13.1, 6.9 Hz, 2H), 3.08 (t, *J* = 7.7 Hz, 2H), 2.66 (s, 6H), 2.60 – 2.52 (m, 2H), 1.81 – 1.61 (m, 2H), 1.57 – 1.50 (m, 2H), 1.46 (s, 9H), 1.42 – 1.35 (m, 2H).

^{13}C NMR (125 MHz, $CDCl_3$) δ 173.2, 172.0, 157.4, 155.6, 143.2, 142.5, 134.5, 132.1, 130.6, 130.3, 128.2, 119.3, 80.0, 63.1, 53.1, 52.4, 39.2, 38.0, 32.6, 31.4, 29.7, 28.9, 28.3, 22.6, 14.9, 14.1.

HRMS (m/z, ESI): calcd for $C_{32}H_{41}BF_2N_4O_5Na^+$ $[M+Na]^+$: 633.3030, found 633.3026.

(*S*)-5,5-Difluoro-10-(4-(6-(methoxycarbonyl)-2,2-dimethyl-4,12,19-trioxo-3-oxa-5,11,18-triazahenicosan-21-yl)phenyl)-3,7-dimethyl-5*H*-dipyrrolo[1,2-*c*:2',1'-*f*][1,3,2]diazaborinin-4-ium-5-uide (14)

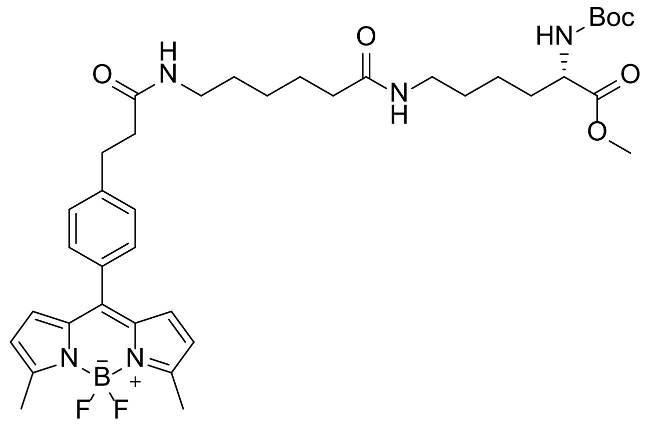

To a mixture of **2** (10 mg, 0.03 mmol, 1 eq), DIC (6 mg, 0.04 mmol, 1.5 eq) and OxymaPure (6 mg, 0.04 mmol, 1.5 eq) in DMF (1 mL) was added Boc-Lys(Ahx)-OMe (15 mg, 0.04 mmol, 1.5 eq), and the reaction was stirred at r.t. overnight. Then, the solvent was removed under reduced pressure and the crude was purified by column chromatography (EtOAc → DCM:MeOH, 95:5) to give compound **14** as an orange solid (7 mg, 36% yield).

^{1}H NMR (500 MHz, $CDCl_3$) δ 7.43 (d, *J* = 8.2 Hz, 2H), 7.34 (d, *J* = 8.2 Hz, 2H), 6.73 (d, *J* = 4.1 Hz, 2H), 6.29 (d, *J* = 4.1 Hz, 2H), 5.78 (s, 1H), 5.68 (s, 1H), 5.14 (d, *J* = 7.6 Hz, 1H), 4.42 – 4.28 (m, 1H), 3.75 (s, 3H), 3.32 – 3.23 (m, 4H), 3.08 (t, *J* = 7.7 Hz, 2H), 2.67 (s, 6H), 2.56 (t, *J* = 7.7 Hz, 2H), 2.16 (t, *J* = 7.3 Hz, 2H), 1.89 – 1.75 (m, 2H), 1.67 – 1.62 (m, 3H), 1.56 – 1.50 (m, 4H), 1.46 (s, 9H), 1.40 – 1.33 (m, 4H).

^{13}C NMR (125 MHz, $CDCl_3$) δ 173.2, 173.1, 171.9, 157.4, 155.6, 143.3, 142.6, 134.5, 132.1, 130.6, 130.4, 128.3, 119.3, 80.0, 63.1, 53.2, 52.3, 39.2, 39.1, 38.0, 36.2, 32.5, 31.9, 31.5, 29.70, 29.66, 29.4, 29.1, 28.9, 28.3, 26.3, 24.9, 22.7, 22.6, 14.9, 14.12, 14.06.

HRMS (m/z, ESI): calcd for $C_{38}H_{53}BF_2N_5O_6^+$ $[M+H]^+$: 724.4051, found 724.4065.

(*S*)-10-(4-(3-((4-(2-((*Tert*-butoxycarbonyl)amino)-3-ethoxy-3-oxopropyl)phenyl)amino)-3-oxopropyl)phenyl)-5,5-difluoro-3,7-dimethyl-5*H*-dipyrrolo[1,2-*c*:2',1'-*f*][1,3,2]diazaborinin-4-ium-5-uide (15)

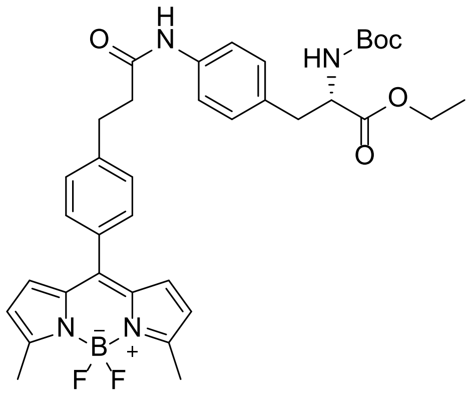

A mixture of **2** (29 mg, 0.08 mmol, 1 eq), PyOxim (52 mg, 0.1 mmol, 1.2 eq) and Boc-aPhe-OEt (31 mg, 0.10 mmol, 1.2 eq) in DMF (2 mL) was stirred at r.t. for 3 h in the presence of DIPEA (21 μL, 0.12 mmol, 1.5 eq). Then, the solvent was removed under reduced pressure and the crude was purified by column chromatography (Hex:EtOAc, 6:4) to give compound **15** as an orange solid (16 mg, 31% yield).

^{1}H NMR (500 MHz, $CDCl_3$) δ 7.37 – 7.27 (m, 6H), 7.04 – 6.96 (m, 3H), 6.63 (d, *J* = 4.1 Hz, 2H), 6.18 (d, *J* = 4.1 Hz, 2H), 4.90 (br, d, *J* = 7.5 Hz, 1H), 4.46 (br, d, *J* = 6.5 Hz, 1H), 4.08 (t, *J* = 7.1 Hz, 2H), 3.11 – 2.96 (m, 4H), 2.66 (t, *J* = 7.5 Hz, 2H), 2.58 (s, 6H), 1.35 (s, 9H), 1.16 (d, *J* = 7.1 Hz, 3H).

^{13}C NMR (125 MHz, $CDCl_3$) δ 170.7, 168.8, 156.5, 154.1, 141.9, 141.4, 135.5, 133.5, 131.3, 129.7, 129.3, 129.0, 127.3, 118.9, 118.3, 78.9, 60.4, 53.4, 37.9, 36.7, 30.9, 30.1, 28.7, 28.3, 27.3, 21.7, 13.9, 13.2, 13.1.

HRMS (m/z, ESI): calcd for $C_{36}H_{41}BF_2N_4O_5Na^+$ $[M+Na]^+$: 681.3030, found 681.3027.

Coupling of unnatural amino acids (UAAs) to pdCpA. CDI (7 mg) was dissolved in anhydrous DMF (45 µL) and added to the Boc-protected UAA (average:10-12 mg) in a 1.5 mL microcentrifuge tube. The reaction was vortexed and incubated at r.t. for 3 min. pdCpA (2.5 mg) was dissolved in 55 µL of water (pH 8.3) and mixed with a solution containing CDI and the Boc-protected UAA using a pipette and vortexed vigorously until the solution became clear. The resulting reaction mixture was separated into four 1.5 mL microcentrifuge tubes and diluted with 800 µL THF to remove unreacted Boc-UAA. The solution was centrifuged at max speed for 5 min and the pellet was dried under N_2 and then dissolved in a mixture of H_2O:ACN (75:25). The product was then purified using semi-preparative HPLC and lyophilized. The Boc-protected pdCpA-UAA conjugate was transferred to a 1.5 mL microcentrifuge tube with 300 µL of an ice-cold mixture of TFA: ACN (5:95). The reaction was stirred on ice for 10 min and the volume was reduced to 5 µL by using N2, and the product was subsequently precipitated in 900 µL Et_2O. The solution was then centrifuged for 5 min at max speed and the pellet was dried at r.t. for 10 min. The pellet was then dissolved in DMSO to yield a 3 mM solution for ligation experiments or stored at -80°C for future use.

RNA ligation and analysis. For ligation reactions, 300 µg of truncated (lacking the CA terminal motif) McTrp tRNA was dissolved in 375 µL 10 mM HEPES. The solution was supplemented with 2.5 mM $MgCl_2$ and folded by heating to 95 °C for 3 min followed by incubation at r.t. for 20 min. A ligation master mix was made by mixing 29 µL water, 8.3 µL T4 RNA ligase buffer (from 10×), 1 µL ATP (10 mM) and 5 µL T4 RNA ligase 1. The reaction was then assembled on ice in a 1.5 mL microcentrifuge tube by adding 8 µL pdCpA-UAA (3 mM), 42 µL ligation master mix and 30 µL folded

tRNA solution. The reaction was incubated at 4°C for 1 h. The UAA-tRNA was extracted with 500 µL phenol:chloroform (5:1) supplemented with sodium acetate (0.1 M pH 5.2), precipitated with 900 µL EtOH, washed with 500 µL 70% EOH and air dried. To determine the aminoacylation efficiencies, 500 ng of UAA-$tRNA_{CUA}$ were diluted in Novex^{TM} TBE-urea Sample Buffer (2×) and loaded onto a TBE urea gel (15%). Electrophoresis was carried out at 120 V for 4 h in TBE. The gel was then scanned for fluorescence using a Fuji FLA-5100 fluorescence image analyzer and subsequently stained with SYBR Gold Nucleic Acid Gel Stain and visualised under UV. The yields were calculated by quantifying the intensity of the bands using FIJI.

Cloning of IL-33 mutants into pSANG10 vector. Our group has earlier used pSANG10-7D12 plasmid for periplasmic expression of a nanobody, 7D12.[1] This plasmid was digested with NdeI and HindIII to remove the coding DNA sequence (CDS) for the 7D12 fragment. After digestion, the reaction mixture was run on a 1% agarose gel. The band corresponding to pSANG10 backbone was excized and extracted using QIAquick Gel Extraction Kit. The codon optimized CDS for wild-type IL-33 with an N-terminal PelB leader peptide, C-terminal 6×His tag and Gibson overhangs (Figure S21) was subsequently cloned into the pSANG10 backbone using Gibson cloning. After cloning, the sequence of pSANG10_IL-33 was confirmed by Sanger sequencing. Note that IL-33 mutants used in our investigations were modified by substituting all cysteine residues with serine residues (i.e., C208S, C227S, C232S and C259S). This change would prevent the formation of disulfide bonds that leads to inactivation of the cytokine. The amber mutants of IL-33, viz. pSANG10_IL-33-143TAG and pSANG_IL33-163TAG, were prepared by using quick change site-directed mutagenesis and pSANG10_IL-33 plasmid as a template. For preparing

pSANG10_IL-33-143TAG plasmid, primers hIL33_ser_Y143tag_r and hIL33_ser_Y143tag_f were employed, and for preparing pSANG_IL33-163TAG plasmid, primers hIL33_ser_Y163tag_r and hIL33_ser_Y162tag_r (Figure S22).

Cloning of DHFR-TAG2. The NEBExpress® Control DHFR-His Plasmid supplied with the NEBExpress® cell-free *E. coli* Protein Synthesis System was subjected to site directed mutagenesis using the Q5® Site-Directed Mutagenesis Kit. Primers were designed following the manufacturer's instructions to insert a TAG codon at position 2.

Cell free protein expression and characterization. Cell-free translation reactions were carried out using the NEB Express cell-free *E. coli* Protein Synthesis System kit following the manufacturer's instructions. The kit was supplemented with the DNA template for the desired protein (20 ng μL^{-1}), UAA-tRNA (8 μM), 1.5 units μL^{-1} RNase Inhibitor Murine and Api 137 (50 $\mu g\ mL^{-1}$). Reactions (5-250 μL) were incubated in either 1.5 mL microcentrifuge tubes or 0.2 ml PCR tubes at 37°C for 4 h. Reactions were analyzed by running 2 μL of the translation mix in parallel to 2 μL of PageRuler™ Prestained Protein Ladder (for Western blotting) and 0.5 μL of BenchMark™ Fluorescent Protein Standard (for in-gel fluorescence) in 4-12% NuPAGE Bis-Tris protein gels following the manufacturer's instructions. In-gel fluorescence was measured using Fuji FLA-5100 fluorescence image analyzer (λ_{exc}: 473 nm, LPB emission filter). Following fluorescence analysis, gels were transferred to a nitrocellulose membrane using an iBlotTM 2 Gel Transfer Device for Western Blot analysis. Membranes were blocked in 10% w/v non-fat dry milk in TBS supplemented with 0.1% Tween-20 for 1 h at r.t. Next, an anti-human IL-33 antibody was diluted in

1% w/v non-fat dry milk in TBS-T in a 50 mL tube. The membranes were transferred to the tube and incubated overnight at 4°C with gentle shaking. The following day, the membranes were washed 3 times with 25 mL TBS-T for 10 min each before incubation with an anti-goat HRP-conjugated secondary antibody for 1 h at r.t. The membranes were subsequently washed 3 times with 25 mL TBS-T for 10 min each before adding Enhanced Chemiluminescence and scanning in a C-DiGit ® Blot Scanner. Images were analyzed in ImageLab.

Protein labeling using CuAAC. 4-azido-L-phenylalanine was incorporated into the IL-33 sequence (position 143) as detailed above. 10 μL NEBexpress reaction was added to 10 μL $CuSO_4$, THPTA, sodium ascorbate (1 mM in H_2O), 1 μL BODIPY-alkynes (DMSO), 9 μL ACN and 50 μL H_2O. Reactions were left stirring at 4°C for 1 h in a 1.5 mL microcentrifuge tube. Subsequently, reactions were stopped by the removal of $CuSO_4$, THPTA and sodium ascorbate using Zeba™ spin desalting columns (7K MWCO 0.5 mL) that had been equilibrated in PBS. Reactions were analyzed by running 4-12% NuPAGE Bis-Tris protein gels and in-gel fluorescence was measured using Fuji FLA-5100 fluorescence image analyzer (λ_{exc}: 473 nm, LPB emission filter).

Expression and purification of amber mutants of IL-33 with site-specifically incorporated photocaged tyrosine (pcY). Chemically competent BL21(DE3)pLysS cells containing pULTRA-pcY plasmid were transformed with pSANG10_IL-33, pSANG10_IL-33-143TAG and pSANG_IL33-163TAG plasmids. pULTRA-pcY plasmid has been prepared earlier.[1] After transformation, cells were recovered in 0.5 mL SOB medium for 1 h at 37 °C. 50 μL of recovered cells were transferred onto LB-

agar plates supplemented with 50 µg mL^{-1} kanamycin and 100 µg mL^{-1} spectinomycin. The plates were incubated overnight (37 °C, 16 h). A single colony from each plate was used to inoculate 10 mL of 2×TY-GKS media (2×TY media with 4% glucose, 50 µg mL^{-1} kanamycin and 100 µg mL^{-1} spectinomycin) and incubated overnight (37 °C, 220 rpm, 16 h). The next day, this culture was used to inoculate fresh 5 mL 2×TY-GKS media to an OD_{600} = 0.1. This was then incubated until OD_{600} reached 0.4-0.6 (37 °C, 220 rpm, 2-3 h), at which point IPTG (1 mM final concentration) was added to induce the expression of IL-33 and the culture was split into two, one half supplemented with 2 mM pcY (positive samples in Figure S13) and to the other half nothing was added (negative samples in Figure S13). The cultures were incubated overnight (30 °C, 160 rpm, 16 h). The following day, cells were pelleted (3,200*g*, 4 °C, 10 min), the supernatant was discarded, and the cells were resuspended in 250 mL periplasmic extraction buffer-1 (20% sucrose, 100 mM Tris-HCl, 1 mM EDTA, pH 8.0). The resuspended cells were incubated on ice for 30 min and then centrifuged (10,000*g*, 4°C, 10 min). The supernatant was removed and stored at 4 °C (periplasmic fraction-1). The resulting pellet was resuspended in 250 mL periplasmic extraction buffer-2 (5 mM $MgCl_2$) and incubated on ice for 20 min. The samples were centrifuged (10,000*g*, 4°C, 10 min) and the supernatant was collected (periplasmic fraction-2). Both periplasmic fractions were combined, passed through a 0.2 µm filter, and analyzed by Western blot. 20 µL periplasmic fraction was used for analysis using Western blot. Nu-PAGE LDS loading buffer was added to the samples (20 µL), heated at 95°C for 15 min, centrifuged (13,000*g*, 15 min, 4 ºC) and loaded on a 4-12% Bis-Tris gel. After running the gel, the proteins were transferred to a nitrocellulose membrane using iBLOT 2. After transfer, the membrane was transferred into a blocking buffer (10% milk, PBS+Tween 0.1%) and incubated for 1 h (gentle rocking, r.t.). After removing the

blocking buffer, the membrane was incubated with primary antibody (Mouse-anti-6×-HIS tag, Invitrogen) at 1:1000 dilution in a solution of 1% milk in PBST (PBS+Tween 0.1%) (1 h, r.t., gentle rocking). After incubation with primary antibody, the membrane was washed with PBST (5 min, gentle rocking). The washing step was repeated 3 times. Subsequently, the membrane was incubated with secondary antibody (Anti-mouse, IgG, HPR-linked, Cell Signaling Technology) at 1:3000 dilution in a solution of 1% milk in PBST (PBS+Tween 0.1%) (overnight at 4 °C, gentle rocking). After incubation with secondary antibody, the membrane was washed with PBST (5 min, gentle rocking). The washing step was repeated 3 times. The membrane was developed using SuperSignal chemiluminescent Substrate and imaged using BIORAD GelDoc XR+ (Figure S13). Subsequently, large scale expression (500 mL) of IL-33 was performed similarly. IL-33 and its mutants containing pcY were subsequently purified via the 6×-HIS tag. A similar protocol to that reported for the purification of the nanobody 7D12 was employed for these experiments.[2] After purification, the identity of IL-33 mutants was confirmed by mass spectrometry (Figure S23).

Fluorescence spectroscopy. A) Turn-on experiments. Phosphatidylcholine-cholesterol liposomes (purchased from Liposoma) were serially diluted in PBS, and 50 mL of each dilution was transferred into a 96-well flat bottom plate in triplicates. Subsequently, 10 mM of BODIPY compounds **1**-**6** were added to each well and the plate was incubated at r.t. for 5 min in the dark. Fluorescence intensity measurements were taken on a Cytation 3 spectrophotometer (λ_{exc}: 480 nm) and data were analyzed in GraphPad Prism 9. B) Fluorescence emission upon ST2 binding. 2 mL of each IL-33 protein (50 μg mL^{-1}) were mixed with 2 mL PBS (negative control) or 2 mL recombinant ST2-Fc (200 μg mL^{-1}) for 5 min. The fluorescence emission signals of the

resulting solutions were measured using a Cytation 3 spectrophotometer (λ_{exc}: 485 nm, λ_{em}: 528 nm).

IL-33-GFP functional assays. IL-33 was acquired from in vitro cell cultures and released by freeze-thaw necrosis, following the reported protocol.[3] Cell lines, including untransfected HEK293 and HEK293_IL-33_GFP (both with 24 h tetracycline treatment) were resuspended at a concentration of 5 × 10^6 cells mL^{-1}. The suspensions underwent 3 freeze-thaw cycles on dry ice. After thawing, the suspensions were centrifuged at 4 °C, 15,000 rpm for 15 min, and the resulting supernatants were collected. IL-33-GFP released during freeze-thaw necrosis was subsequently confirmed by ELISA and Western Blot analysis. Supernatants were then loaded onto freshly harvested bone marrow cells (5 × 10^6 cells $well^{-1}$) from BALB/c mice. All cells were cultured in the presence of IL-2 (10 ng mL^{-1}) and IL-7 (10 ng mL^{-1}) as well as the cell supernatants (1 in 4 dilution). The levels of IL-5 as a direct response to IL-33 downstream signaling was measured by ELISA after 5 days of culture at 37 °C.

pcY-IL-33 binding ELISA assays. 96-well high-binding ELISA plates were coated overnight at 4°C with 1 μg mL^{-1} of ST2-Fc or rHpARI. Plates were washed three times with ELISA wash buffer (PBS+0.05% Tween20), then incubated in ELISA block buffer (PBS+0.5% BSA) for 2 h at 37°C. Subsequently, the pcY-IL-33 mutants were added to the plate in ½ log dilutions (1-0.01 μg mL^{-1}) diluted in block buffer, followed by a 2 h incubation at r.t. and three washes in ELISA wash buffer. Detection of the IL-33 constructs employed a 1:3000 dilution of anti-IL-33 antibody in block buffer with 2 h incubation at r.t. and three washes in ELISA wash buffer. Finally, an anti-goat-HRP

antibody diluted 1:2000 in block buffer was applied to the plate, followed by 2 h incubation at r.t. and three washes in ELISA wash buffer. The enzymatic reaction was initiated by adding 50 µL TBM substrate (Pierce™ TMB Substrate Kit) and stopped with 50 µL 2 M H_2SO_4. Absorbance values at 450 nm and 570 nm were measured in a Cytation 3 spectrophotometer.

Functional assays in HEK cells. HEK-Blue™ IL-33 reporter cells (InvivoGen) were cultured in DMEM supplemented with 10% FBS, 100 U mL^{-1} penicillin, 0.1 mg mL^{-1} streptomycin, 100 µg mL^{-1} normocin and HEK-Blue selection antibiotics. Cells were grown until reaching 80% confluency, then centrifuged at 300*g* for 5 min and re-suspended in DMEM supplemented with 10% FBS, 100 U mL^{-1} penicillin, 0.1 mg mL^{-1} streptomycin at 2.8×10^5 cell mL^{-1}. In inhibitor experiments, ST2-Fc, HpBARI_Hom2[4] and HpARI[3] were added to wells in triplicates to a 96-well flat bottom plate (10 µg mL^{-1}). Next, the proteins IL-33(**1**), IL-33(**3**), IL-33(**6**) and wild-type IL-33 were added in triplicates to the plate in dilutions starting at 100 ng uL^{-1} and diluted serially in 2-fold. 100 µL cell suspensions were added to each well and cultured overnight at 37 °C and 5% CO_2. The plates were centrifuged at 300*g* for 5 min and 20 µL supernatant from each well was transferred to a fresh 96-well flat bottom plate. Subsequently, 180 µL Quanti-Blue solution was added to each well and incubated at 37 °C and 5% CO_2 until a color change was observed (around 30-60 min). Absorbance values at 635 nm were measured using a Cytation 3 spectrophotometer.

Confocal microscopy. HEK-Blue™ IL-33 reporter cells or HMC1.1 cells were plated into laminin-coated 24-well glass chamber slides at 25,000 cells per well and incubated overnight at 37 °C with 5% CO_2. Cells were gently washed with PBS and

DRAQ5 (1:1000) was added to each well before incubating for 15 mins at 37 °C with 5% CO_2. Just before imaging, 10 μL proteins IL-33(**1**), IL-33(**3**) or IL-33(**6**) were added. Cells were imaged using an inverted Leica SP8 confocal microscope (60×) using 488 nM or 561 nM lasers. Data was analyzed using FIJI.

Flow cytometry. HMC1.1 cells were collected via centrifugation, resuspended in FACS buffer (100,000 cells in 100 μL) and strained using a 40 μm cell strainer into 1.5 mL tubes. 50 μL IL-33-(**6**) was then added in different concentrations and cells were incubated at 37 °C with 5% CO_2 for 15 min. Subsequently, cells were washed twice with FACS buffer before analysis in a 6L Fortessa flow cytometer.

Supplementary figures

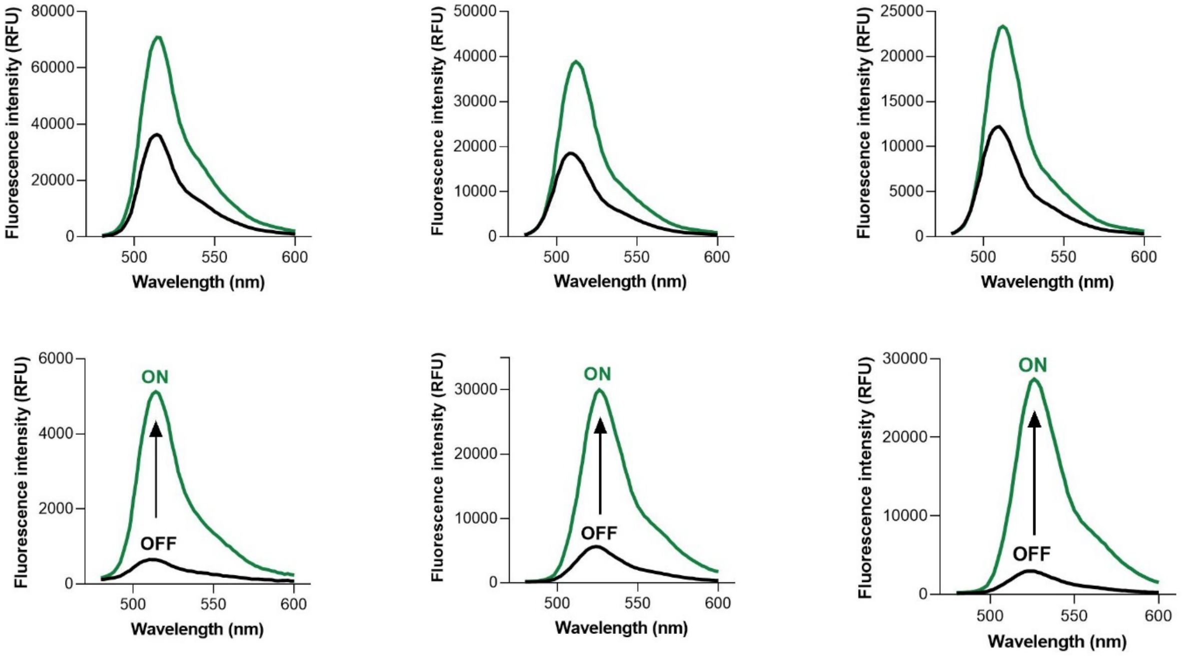

Figure S1. Fluorescence emission plots of compounds 1-6. Representative fluorescence spectra (from n=3) of BODIPY dyes (10 μM) that were either incubated in PBS (black) or in phosphatidylcholine-cholesterol liposomes for 5 min in the dark. Top row: compound **1**, **2** and **3**. Bottom row: compounds **4**, **5** and **6**. Fluorescence intensity measurements were taken on a Cytation 3 spectrophotometer (λ_{exc}: 450 nm). Data were analyzed in GraphPad Prism 9.

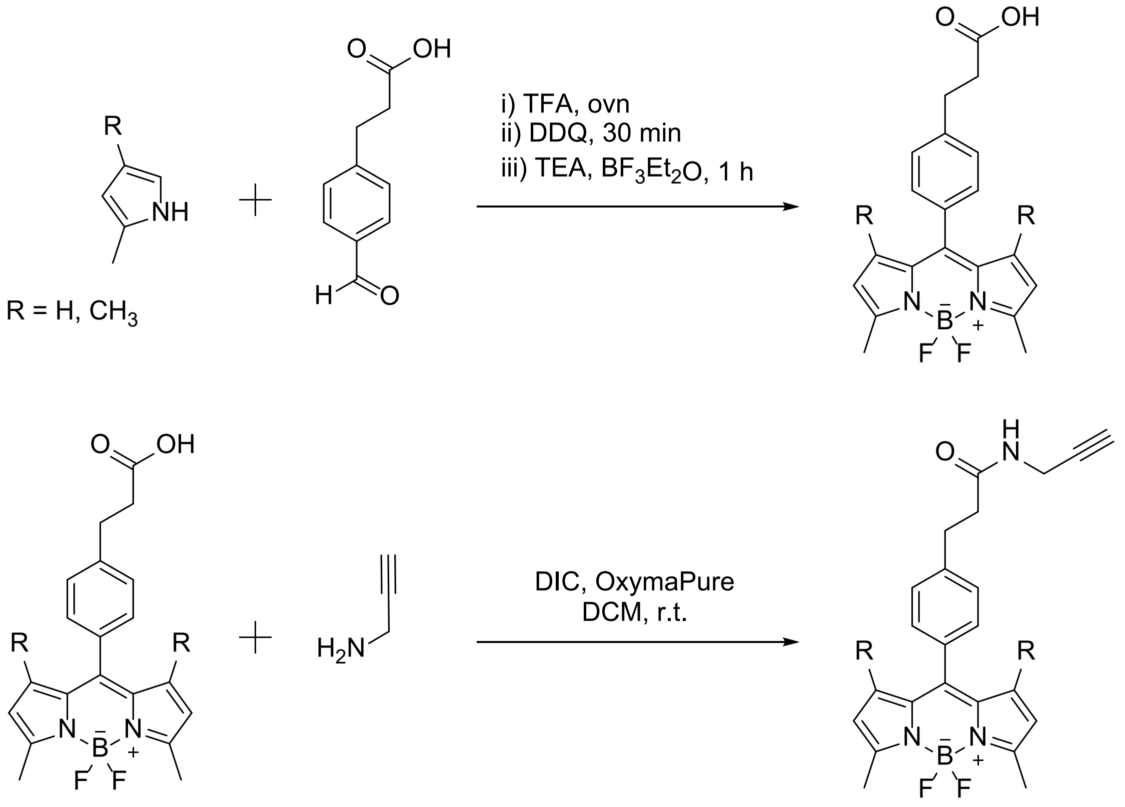

Figure S2. Synthetic route for the synthesis of BODIPY-carboxylic acids (top) and BODIPY-alkynes (bottom).

compound	λ_{abs} (nm)	λ_{abs} (nm)	ε (M^{-1} cm^{-1})
**1**	505	515	96,000
**2**	500	510	73,000
**3**	500	510	77,000
**5**	500	515	48,000
**6**	510	525	49,000

Figure S3. Spectral properties of compounds 1-6. Compounds **1**-**6** were diluted in the detailed solvents and absorbance/emission spectra were measured using a Cytation 3 spectrophotometer (λ_{exc}: 480 nm). Extinction coefficient measurements were obtained using a NanoDrop™ spectrophotometer. All measurements are presented as representative data (from n=3). The optical properties of compound **4** were previously reported in reference.[5]

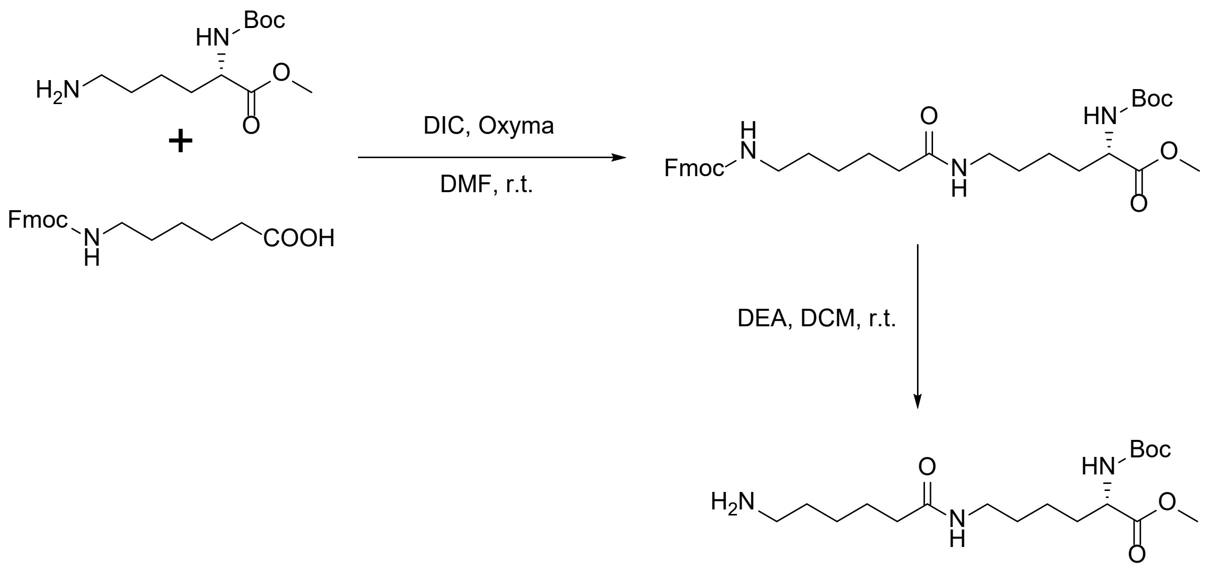

Figure S4. Synthesis of the Lys-Ahx spacer. The solution-phase coupling between Boc-Lys-OMe and Fmoc-Ahx-OH was followed by Fmoc removal with diethylamine in DCM.

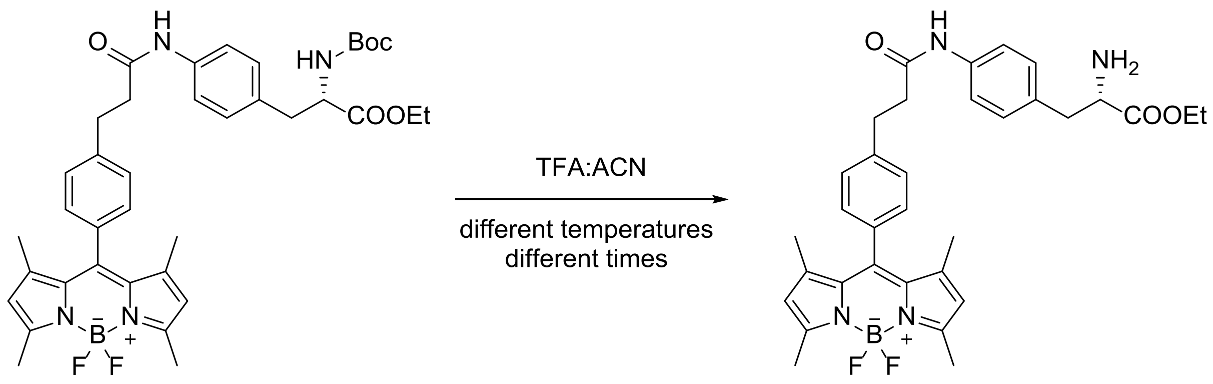

Entry	TFA:ACN	Temperature	Reaction time	% free amine BODIPY
1	50:50	r.t.	30 min	complete degradation
2	50:50	r.t.	10 min	complete degradation
3	50:50	0°C	10 min	complete degradation
4	20:80	r.t.	30 min	<5%
5	20:80	r.t.	10 min	<5%
6	20:80	0°C	10 min	<5%
7	5:95	r.t.	30 min	10%
8	5:95	r.t.	10 min	26%
**9**	**5:95**	**0°C**	**10 min**	**43%**
10	5:95	0°C	60 min	13%

Figure S5. Optimization of Boc deprotection in BODIPY conjugates. We performed Boc removal using compound **9** as model fluorophore and analyzed the degradation of the BODIPY core by HPLC-MS (254 nm).

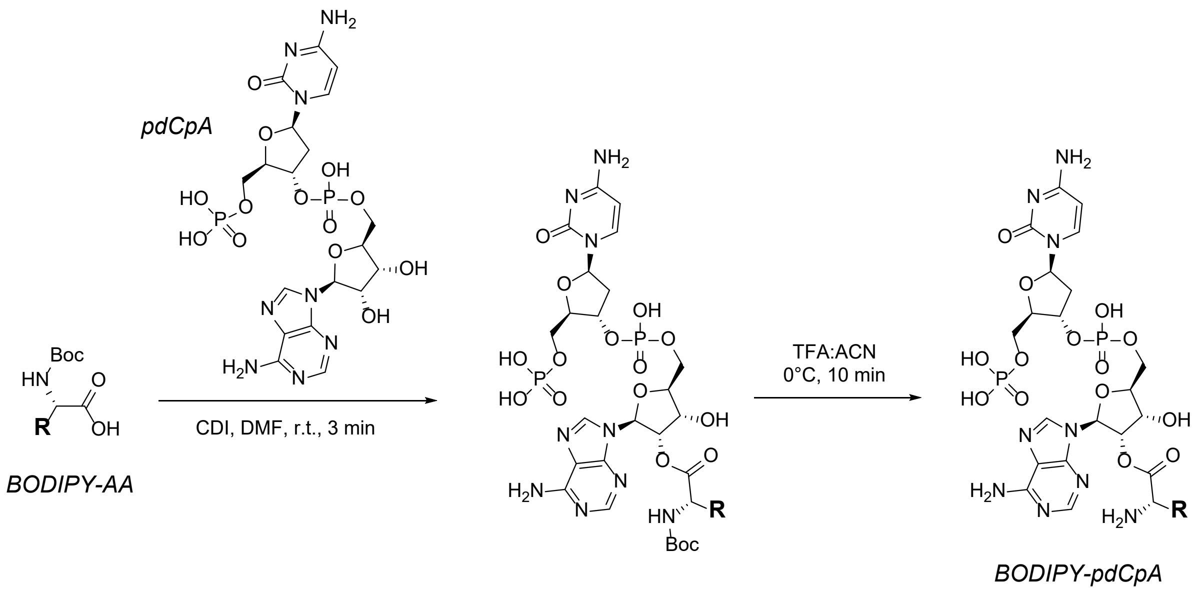

Figure S6. **Synthesis of BODIPY-pdCpA conjugates**. Protected BODIPY amino acids substrates were coupled to the pdCpA dinucleotide and the final products were isolated after Boc removal with the optimized conditions (Figure S5).

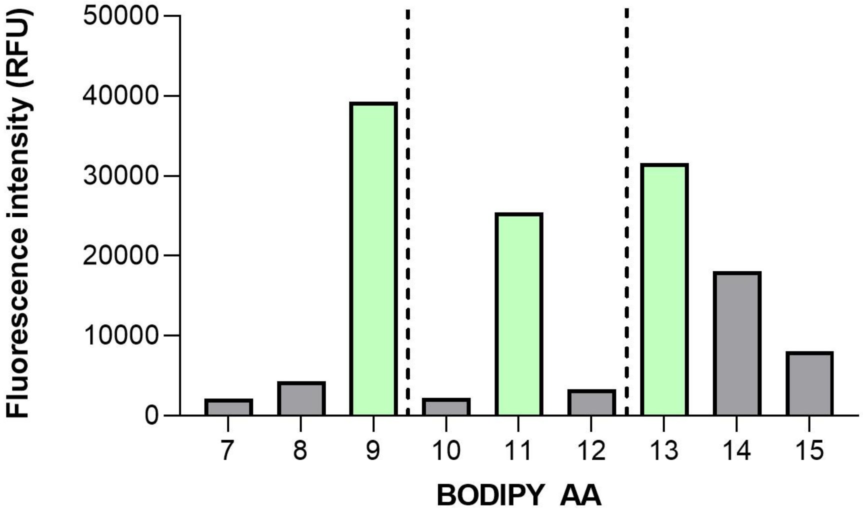

Figure S7. Densitometry analysis of the optimal linkers for chemical aminoacylation. BODIPY-pdCpAs were coupled to McTrp1 tRNA and analyzed by in-gel fluorescence scanning of TBE-urea gels and densitometry quantification. The values presented correspond to the representative gels shown in Figure 3.

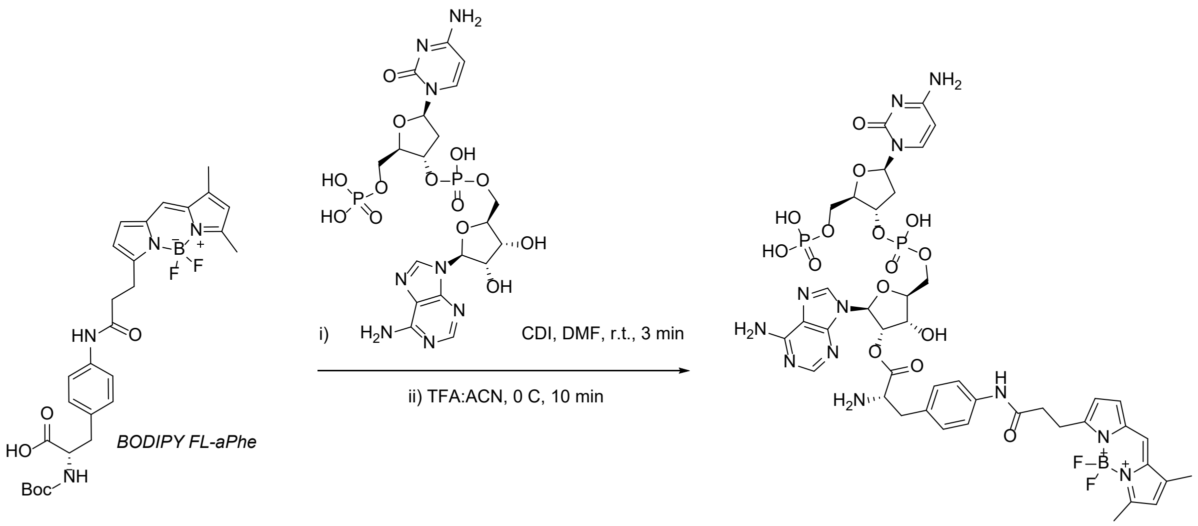

Figure S8. Synthesis of the pdCpA conjugate containing the BODIPY-aPhe amino acid.

Entry	$CuSO_4$	Ligand	Reducing agent	BODIPY 3	azidoPhe	Reaction conversion
**1**	1 mM	THPTA	sodium ascorbate	1 μM	1 μM	++
**2**	1 mM	THPTA	sodium ascorbate	5 μM	1 μM	+++
**3**	**1 mM**	**THPTA**	**sodium ascorbate**	**10 μM**	**1 μM**	**++++**
**4**	1 mM	THPTA	TCEP	1 μM	1 μM	+
**5**	1 mM	THPTA	TCEP	5 μM	1 μM	+
**6**	1 mM	THPTA	TCEP	10 μM	1 μM	+
**7**	1 mM	BTTAA	sodium ascorbate	1 μM	1 μM	+
**8**	1 mM	BTTAA	sodium ascorbate	5 μM	1 μM	++
**9**	1 mM	BTTAA	sodium ascorbate	10 μM	1 μM	+++
**10**	1 mM	BTTAA	TCEP	1 μM	1 μM	+
**11**	1 mM	BTTAA	TCEP	5 μM	1 μM	+
**12**	1 mM	BTTAA	TCEP	10 μM	1 μM	+

Figure S9. Optimization of the CuAAC coupling between the BODIPY fluorophore 3 and azidoPhe. Compound **3** and azidoPhe were incubated under the different conditions listed above, and reactions were monitored using HPLC-MS (254 nm).

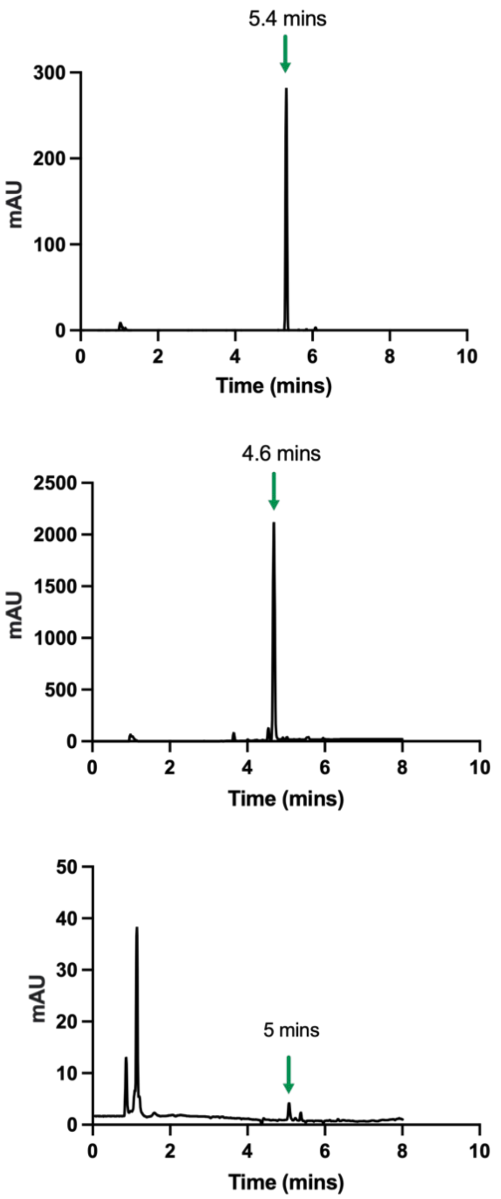

Figure S10**. HPLC-MS traces illustrating the coupling between compound 3 and azidoPhe**. Top) compound **3** (tR: 5.4 min, m/z: 434.2); middle) azidoPhe (tR: 4.6 min, m/z: 207.1); bottom) conjugation product (tR: 5.0 min, m/z: 639.5). UV detection at 254 nm.

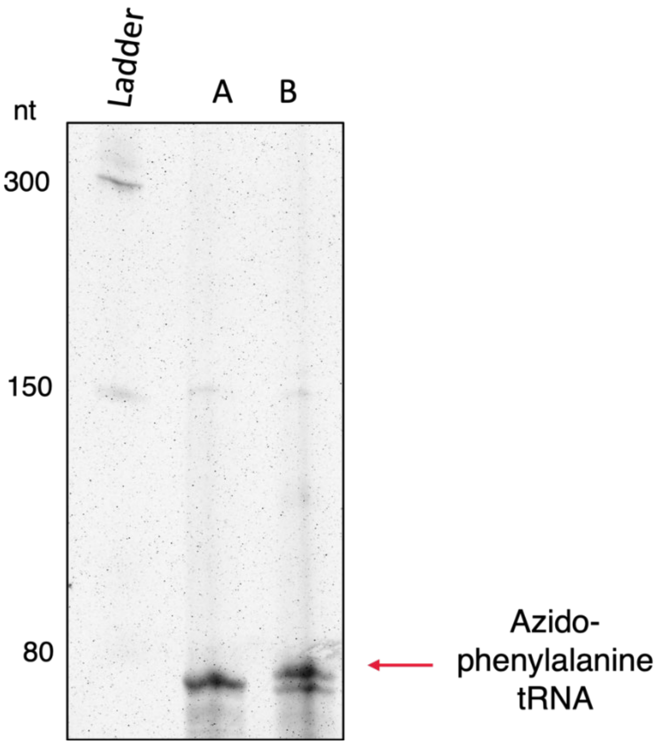

Figure S11. Chemical aminoacylation showing incorporation of azidoPhe. AzidoPhe was conjugated to McTrp1 and analyzed on a TBE-urea gel stained with SYBR gold and visualised under UV. A: McTrp1 tRNA only; B: McTrp1 charged with azidoPhe.

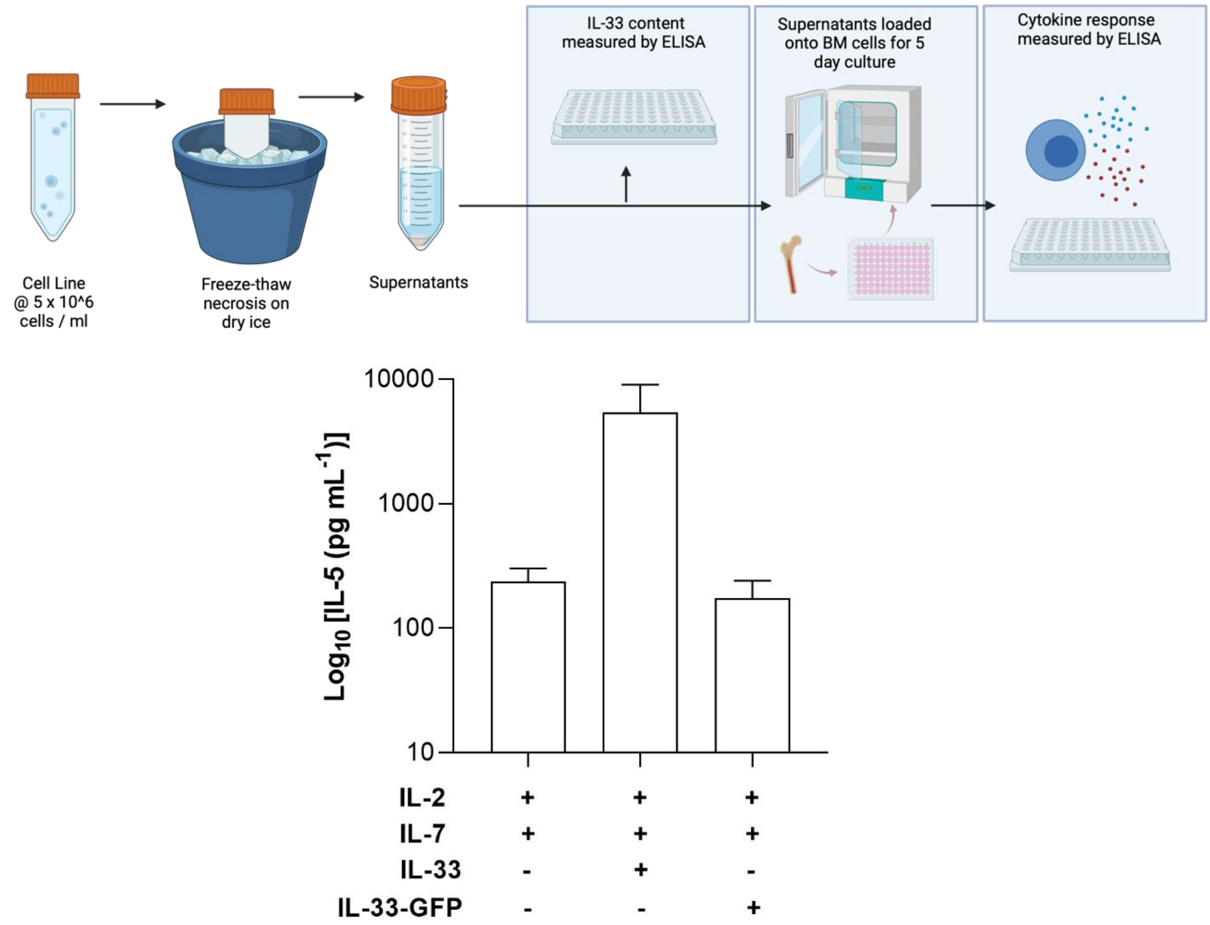

Figure S12. IL-33-GFP functional assays. Top) Cartoon illustrating how IL-33-GFP was released by freeze-thaw necrosis from transfected HEK293_IL-33_GFP cells and the resulting supernatants were collected. Bottom) ELISA assays measuring the release of the interleukin IL-5 from freshly harvested bone marrow cells (5 × 10^6 cells well^{-1}) from BALB/c mice in the presence or absence of IL-33 and IL-33-GFP. All cells were cultured in the presence of IL-2 (10 ng mL^{-1}) and IL-7 (10 ng mL^{-1}). Values presented as means±SD.

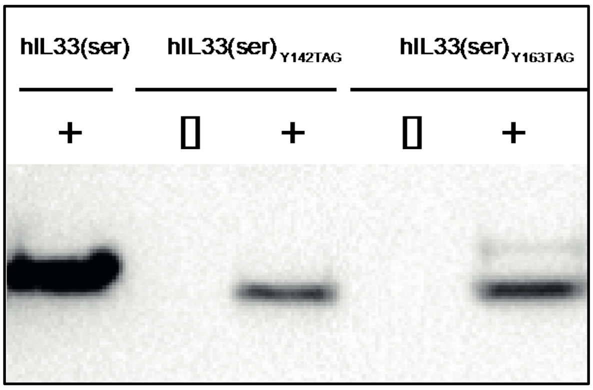

Figure S13. Western blot confirming the expression of wild-type IL-33 (left), IL-33 Y143pcY (middle) and IL-33 Y163pcY (right). IL-33 proteins were extracted and purified from the periplasm of BL21(DE3)pLysS cells and analyzed by Western blotting using an anti-HIS tag antibody. Each conjugate shows expression in the absence (□) or presence (+) of pcY.

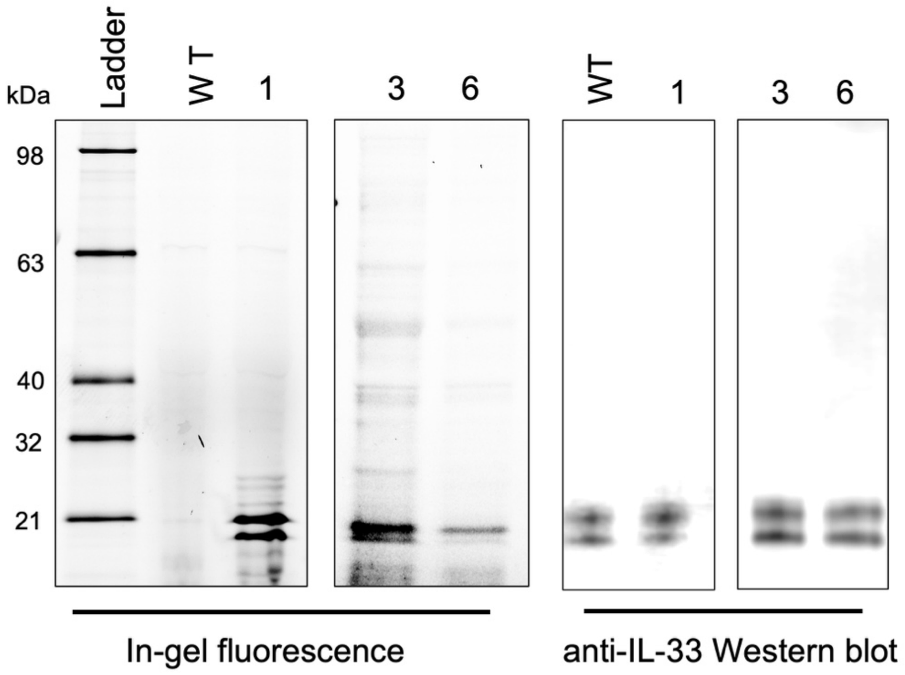

Figure S14. Site-specific incorporation of BODIPY fluorophores into position 143 of the IL-33 protein. Representative SDS-PAGE gels displaying fluorescent bands corresponding to BODIPY-labelled IL-33 proteins (fluorophores **1**, **3** and **6** indicated at the top of the lanes) by in-gel fluorescence analysis (λ_{exc}: 473 nm) and Western blot analysis using an anti-IL-33 antibody.

**protein**	**yield (μg mL^{-1})**
unlabeled IL-33	47.8
IL-33(**1**)	17.7
IL-33(**3**)	12.7
IL-33(**6**)	13.2

Figure S15. Summary of protein yields obtained using cell-free in vitro translation (reaction volumes for each protein: 250 μL).

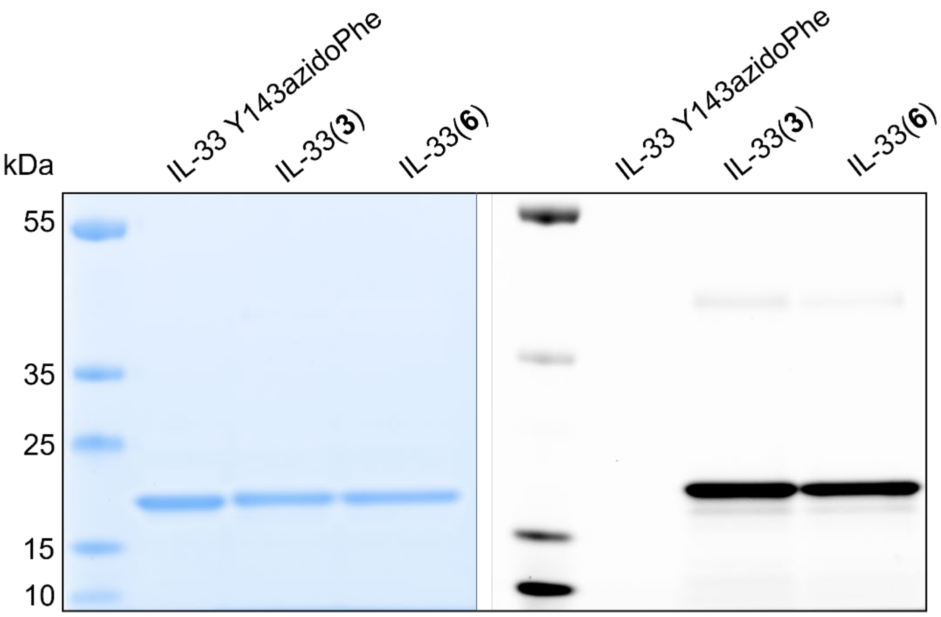

Figure S16. Production of IL-33 Y143azidoPhe in *E. coli* and chemical incorporation of BODIPY fluorophores 3 and 6. Representative SDS-PAGE gels for the unlabeled IL-33 protein and the BODIPY-labeled IL-33(**3**) and IL-33(**6**) proteins after Coomassie staining and in-gel fluorescence analysis (λ_{exc}: 473 nm).

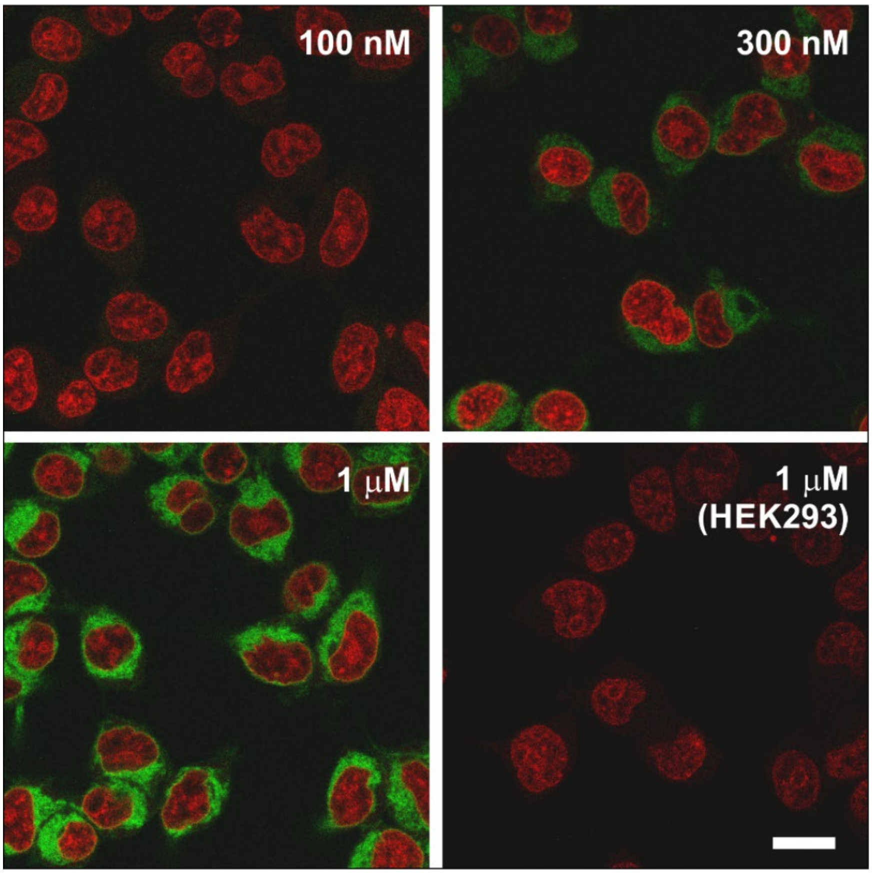

Figure S17. Representative fluorescence confocal microscopy images of ST2-transfected HEK cells and non-transfected HEK293 cells after incubation with IL-33(**6**) (green) at the indicated concentrations and nuclear counterstain DRAQ5 (red). Scale bar: 15 μm.

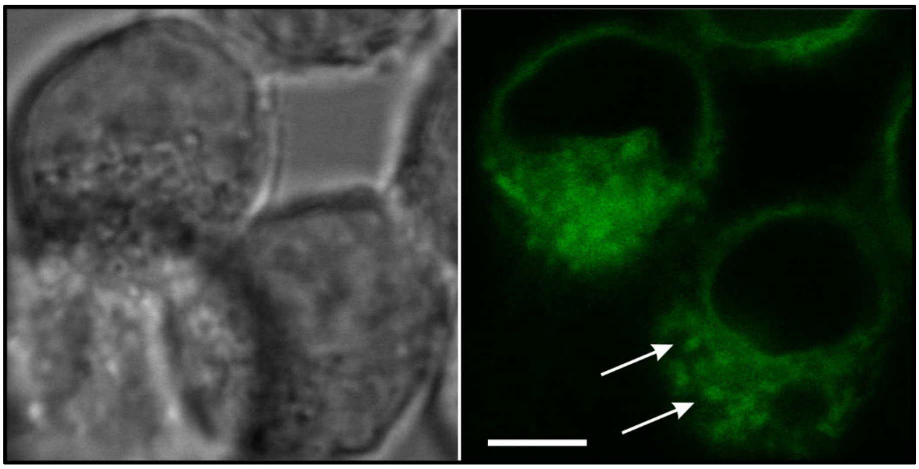

Figure S18. High magnification images of ST2-expressing cells after incubation with IL-33(6). Brightfield and fluorescence confocal microscopy images of transfected HEK-Blue cells after incubation with IL-33(**6**) (green). White arrows point at subcellular lysosomal organelles. Scale bar: 10 μm. Excitation laser for IL-33(**6**): 488 nm.

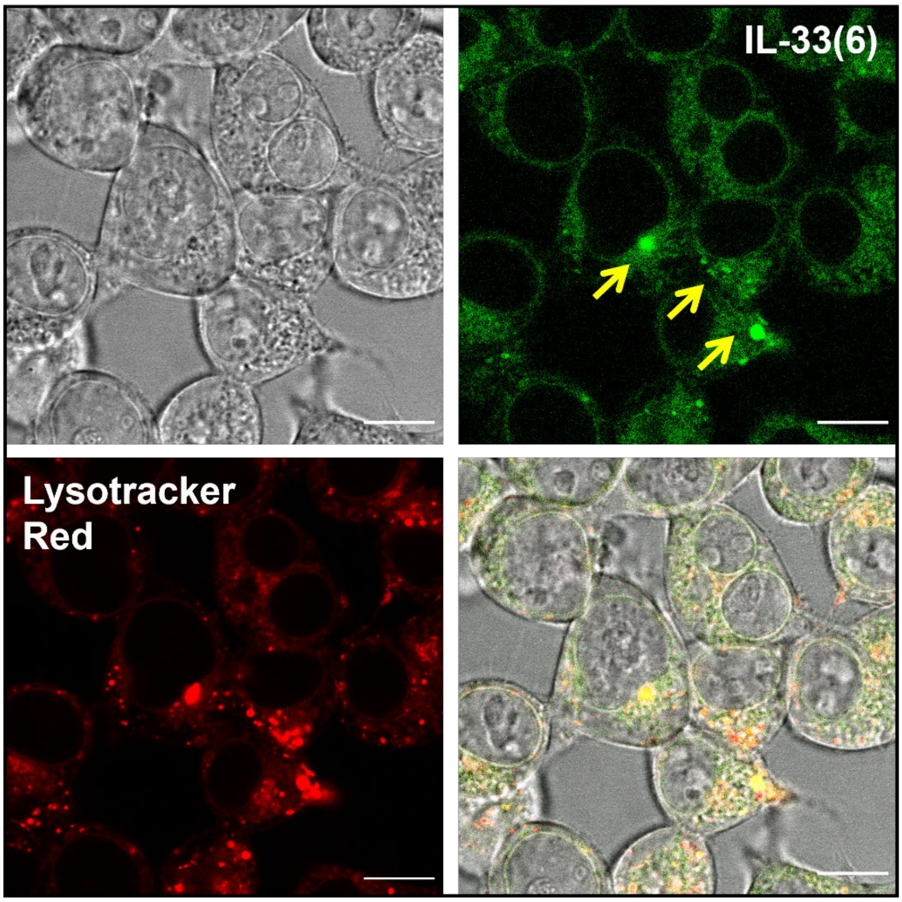

Figure S19. High magnification images of ST2-expressing cells after incubation with IL-33(6) and Lysotracker Red. Representative brightfield and fluorescence confocal microscopy images of HEK-Blue cells after incubation with IL-33(**6**) (1 μM, green) and Lysotracker Red (75 nM, red). Yellow arrows point at subcellular areas of co-localization. Scale bar: 15 μm. Excitation lasers: 488 nm for IL-33(**6**), 561 nm for Lysotracker Red.

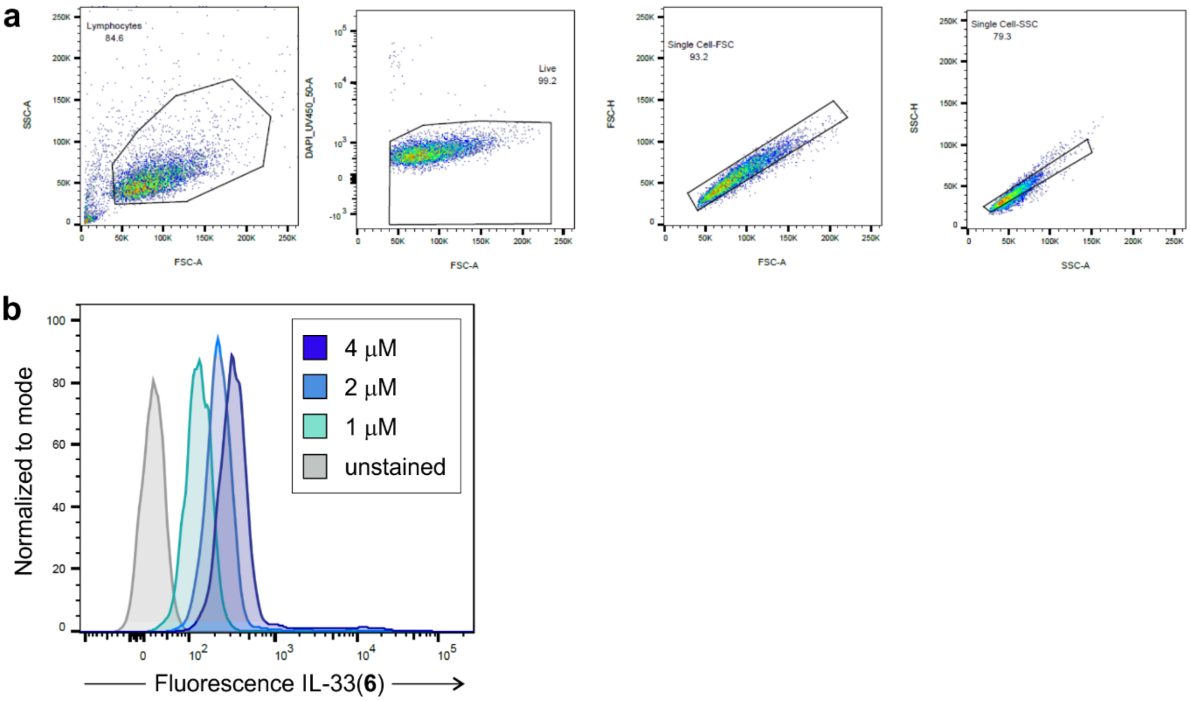

Figure S20. Flow cytometric analysis of human HMC1.1 cells upon incubation with IL-33(6). a) Summary of the gating strategy. b) Histogram of unstained and stained human HMC1.1 cells after incubation with the indicated concentrations of IL-33(**6**) for 15 min at 37 ^{o}C.

TTAACTTTAAGAAGGAGATATACATATGAAATCCCTGATTACGCCTATCGCTGCTGGGTTG
CTTCTGGCGTTCTCTCAATATTCGCTTGCATCGTCAATCACAGGAATCTCCCCGATTACGGA
ATATTTAGCGTCATTGTCCACCTATAACGATCAATCCATTACATTTGCGCTGGAGGATGAGT
CGTATGAGATTTATGTTGAGGACTTAAAGAAGGACGAGAAGAAAGACAAAGTGCTGTTGTCC
TACTACGAGTCTCAACACCCGTCGAATGAGTCGGGGGACGGGGTAGACGGCAAGATGTTAAT
GGTGACTCTGTCTCCGACTAAAGACTTTTGGTTGCACGCTAACAATAAAGAACACTCCGTTG
AGTTGCACAAGAGCGAAAAGCCTCTTCCCGATCAAGCGTTCTTCGTGTTACACAACATGCAC
TCTAATAGCGTCAGCTTCGAGAGCAAGACAGATCCGGGCGTCTTTATCGGAGTCAAAGATAA
CCACCTGGCATTGATCAAAGTTGACTCTTCGGAAAACCTTTCTACAGAGAATATCTTATTTA
AACTGAGCGAAACACATCACCATCATCACCATTGA**TAAAAGCTTTAATAAGTCGAGCACC**

Figure S21. Sequence of gene block inserted into pSANG10 plasmid for generation of pSANG10_(wt)IL-33 plasmid. Bold Black: Gibson overhangs, Underlined: Restriction sites used for cloning, Green: DNA sequence for PelB leader peptide, Black: DNA sequence for expression of wild-type IL-33, and Blue: 6xHis tag.

hIL33_ser_Y143tag_r: 5'-tcctcaacataaatctcctacgactcatcctccagc-3'
hIL33_ser_Y143tag_f: 5'-gctggaggatgagtcgtaggagatttatgttgagga-3’

hIL33_ser_Y163tag_r: 5'- ggtgttgagactcgtactaggacaacagcactttg -3'
hIL33_ser_Y163tag_f: 5'- caaagtgctgttgtcctagtacgagtctcaacacc -3'

Figure S22. Primers used for preparing pSANG10_IL-33-143TAG and pSANG_IL33-163TAG using quickchange site-directed mutagenesis and pSANG10_IL-33 plasmid as a template.

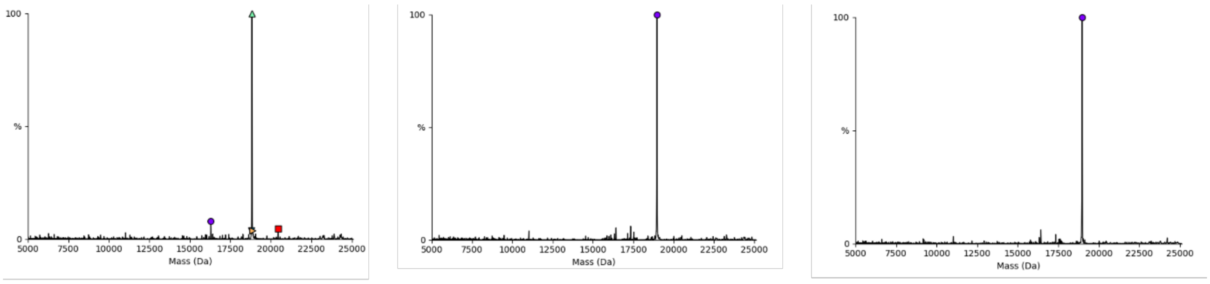

WT IL-33
Expected mass: 18,840 Da
Observed mass: 18,840 Da

IL-33 Y143pcY
Expected mass: 18,974 Da
Observed mass: 18,974 Da

IL-33 Y163pcY
Expected mass: 18,974 Da
Observed mass: 18,974 Da

Figure S23. Mass spectrometry analysis of IL-33 mutants containing pcY. ESI-MS spectra recorded in a Waters Synapt Q-TOF for wild-type IL-33 (left) and the pcY-containing mutants IL-33 Y143pcY (middle) and IL-33 Y163pcY (right).

Supplementary movies

Supplementary Movie 1. Time-lapse fluorescence confocal microscopy images of transfected HEK-Blue cells after incubation with IL-33(**6**) (green) and nuclear counterstain DRAQ5 (red). IL-33(**6**) was added as fluorescence images were recorded. Excitation lasers: 488 nm (for IL-33(**6**)), 561 nm (for DRAQ5). Movie recorded for 5 min and compressed (jpeg) at 10 fps.

Supplementary Movie 2. Time-lapse fluorescence confocal microscopy images of transfected HEK-Blue cells that had been pre-incubated with HpBARI (1 $\mu g\ mL^{-1}$) for 1 h. Cells were incubated with IL-33(**6**) (green) and nuclear counterstain DRAQ5 (red). IL-33(**6**) was added as fluorescence images were recorded. Excitation lasers: 488 nm (for IL-33(**6**)), 561 nm (for DRAQ5). Movie recorded for 5 min and compressed (jpeg) at 10 fps.

Supplementary discussion

Flexizymes (Fxs) are ribozymes that catalyze the specific attachment of 3'-end of tRNA to the activated esters of amino acids to form non-proteinogenic acyl-tRNAs for subsequent translation. After the pioneering work by Suga and co-workers, Fxs have been adapted to incorporate unnatural amino acids (e.g., *N*-methylated or D-amino acids), but there are no studies of their potential compatibility with BODIPY tags. To evaluate this, we synthesized three derivatives of Trp(BODIPY) including active esters (e.g., chlorobenzylthioester (CBT, **4a**), cyanomethylester (CME, **4b**) and dinitrobenzylester (DBE, **4c**), Figure S24) as substrates for eFx and dFx due to their ability to charge bulky, aromatic amino acids. The synthesis of compounds **4a-c** was performed in two steps from the common precursor Fmoc-Trp(BODIPY)-OH, first by esterification of the carboxylic acid followed by Fmoc deprotection under mild basic conditions to preserve the integrity of the reactive esters. The successful isolation of compounds **4a-c** confirms that BODIPY amino acids are compatible with the activating groups required for Fx-mediated bioconjugation. Next, we evaluated the conjugation of the activated esters **4a-c** to the suppressor tRNA *Methanocaldococcus jannaschi* (*Mj*tRNA) with the corresponding Fxs (i.e., dFx was used for compound **4c**; eFx was used for compounds **4a** and **4b**). Several reaction conditions were attempted, including high concentrations of DMSO given the hydrophobicity of Trp(BODIPY) derivatives, different buffers (e.g., HEPES at pH 7.5, bicine at pH 9.5), and variable concentrations of both substrates and Fxs; however, none of the tested conditions led to significant charging of *Mj*tRNA (representative gels shown in Figure S25). During the course of this work, the group of Jewett reported that unnatural amino acids including bulky side chains are not well tolerated by dFx and eFx,[6] which are in agreement with these observations for Trp(BODIPY).

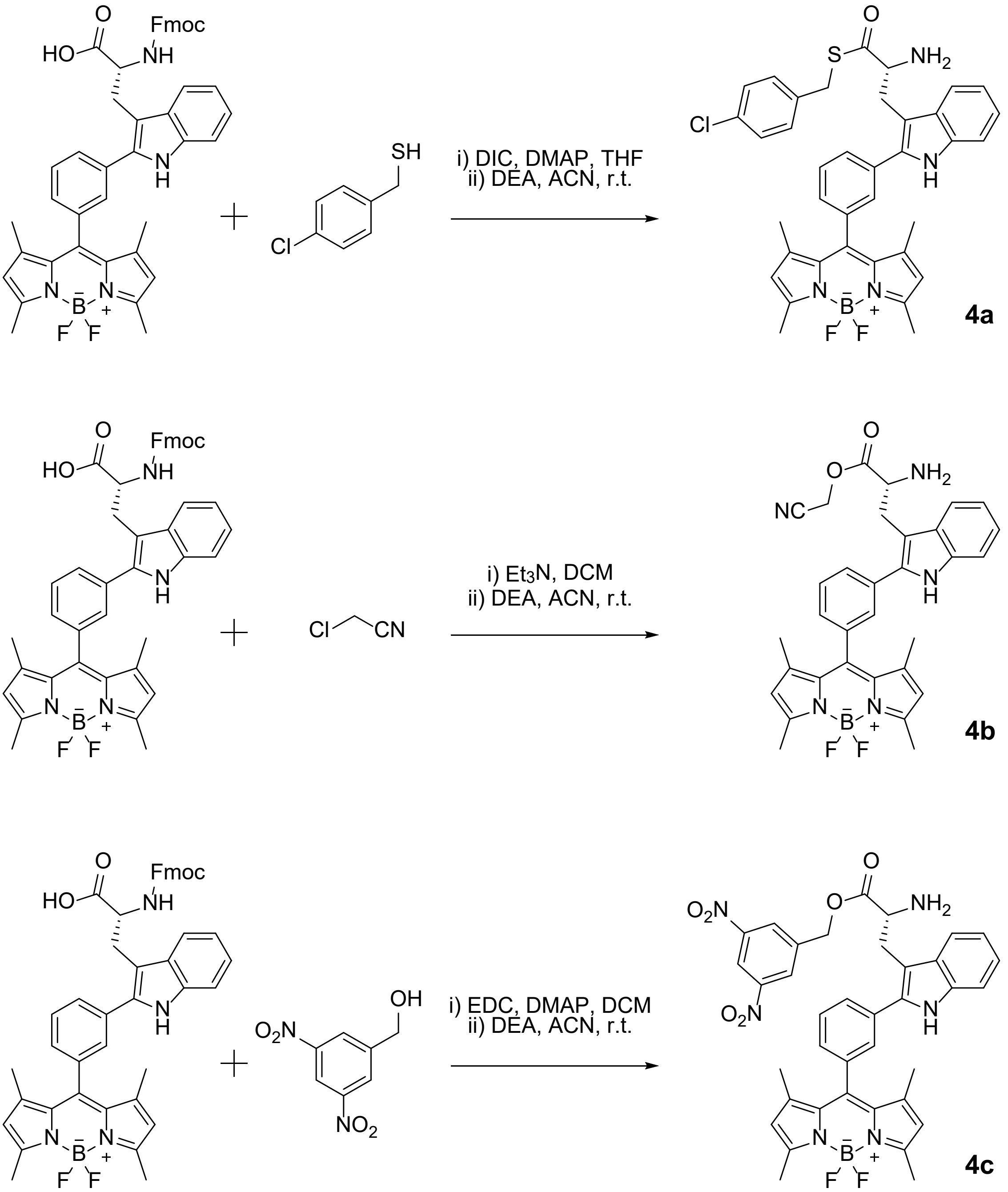

Figure S24. Synthetic route for the synthesis of Trp(BODIPY) reactive esters for flexizyme-mediated aminoacylation.

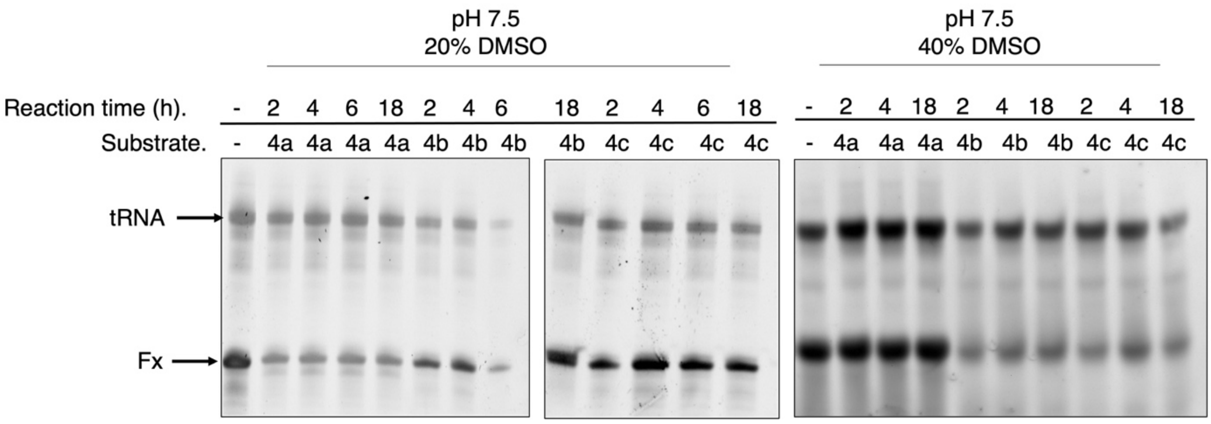

Figure S25. Flexizyme-mediated aminoacylation attempts. Representative TBE-urea gels after attempting flexizyme-catalyzed aminoacylation of MjtRNA by using the different reactive esters of Trp(BODIPY) (compounds **4a**-**4c**) under different conditions. Gel stained with SYBR gold and visualised under UV (λ_{exc}: 302 nm).

NMR spectra

Compound **2** ($CDCl_3$)

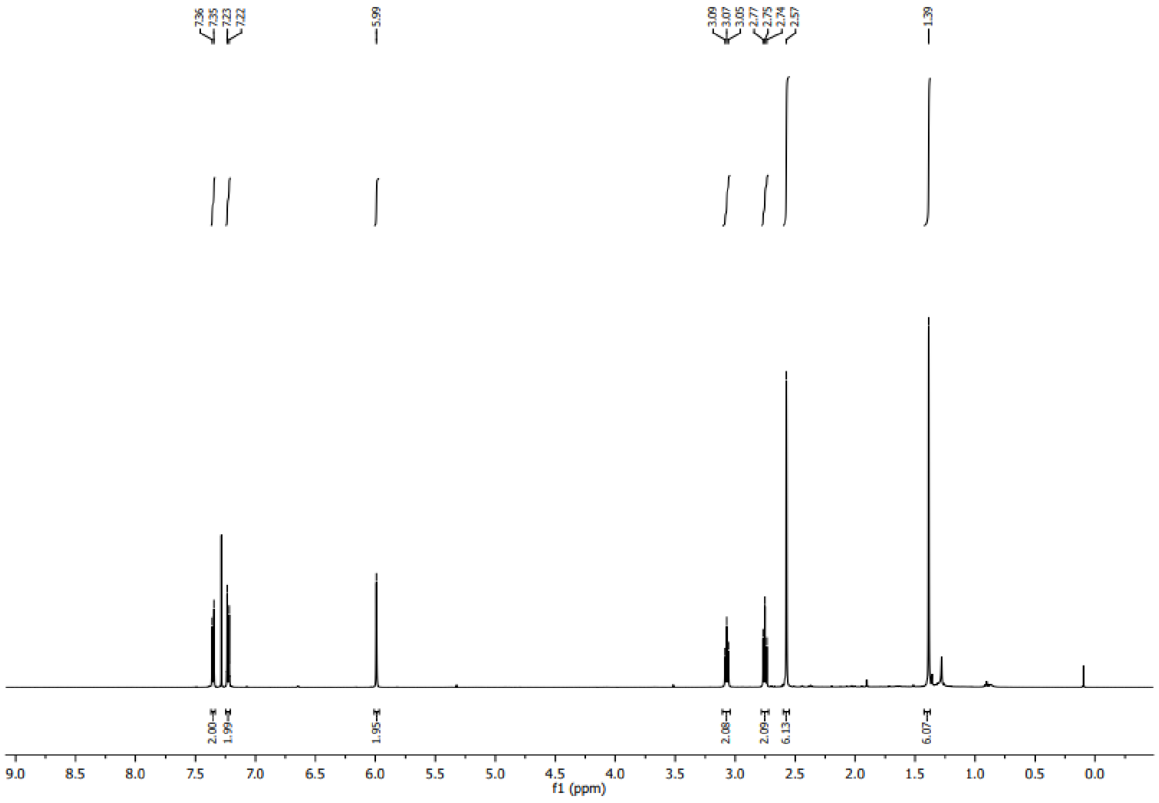

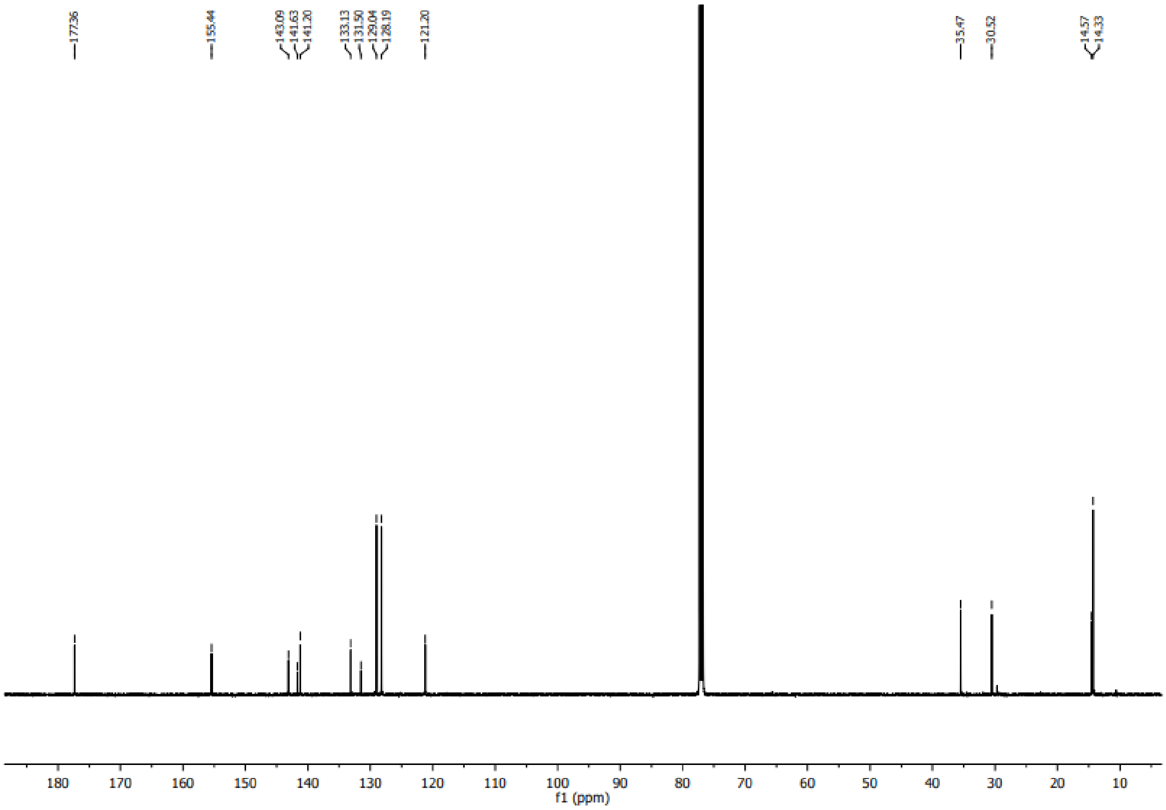

Compound **3** ($CDCl_3$)

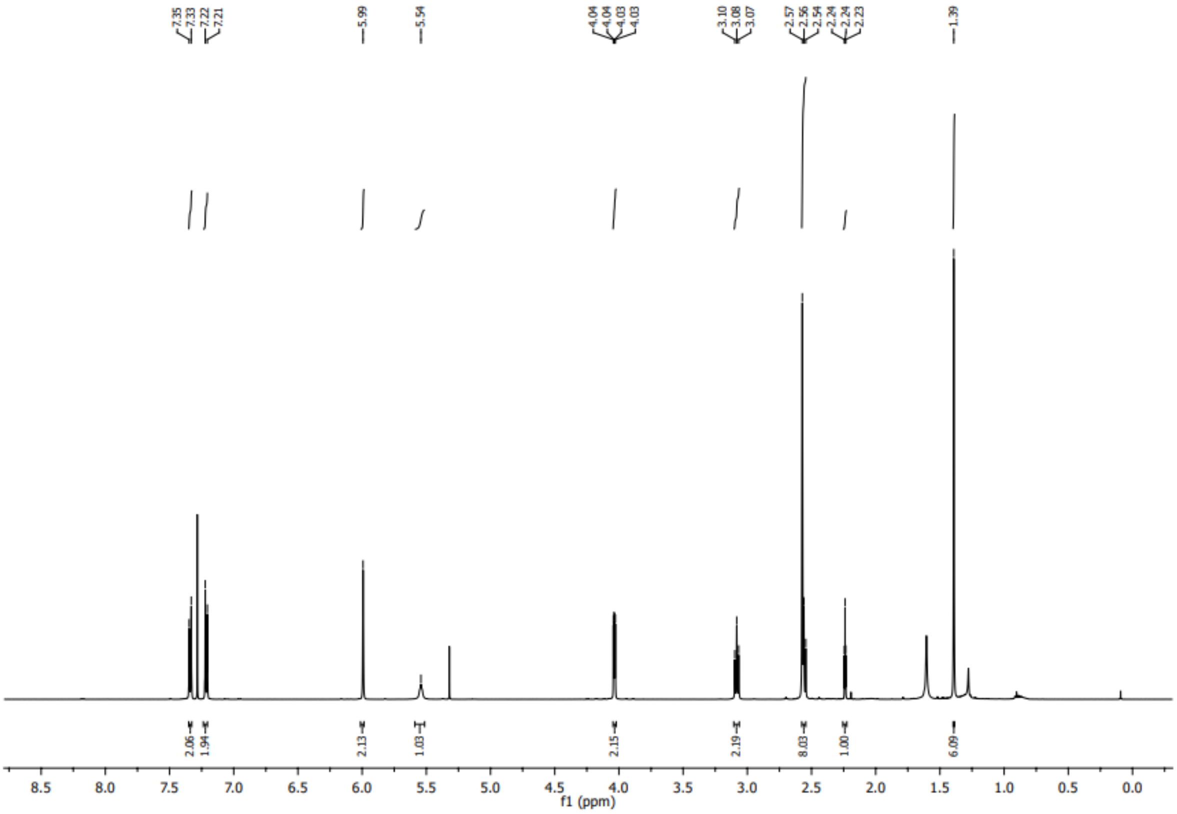

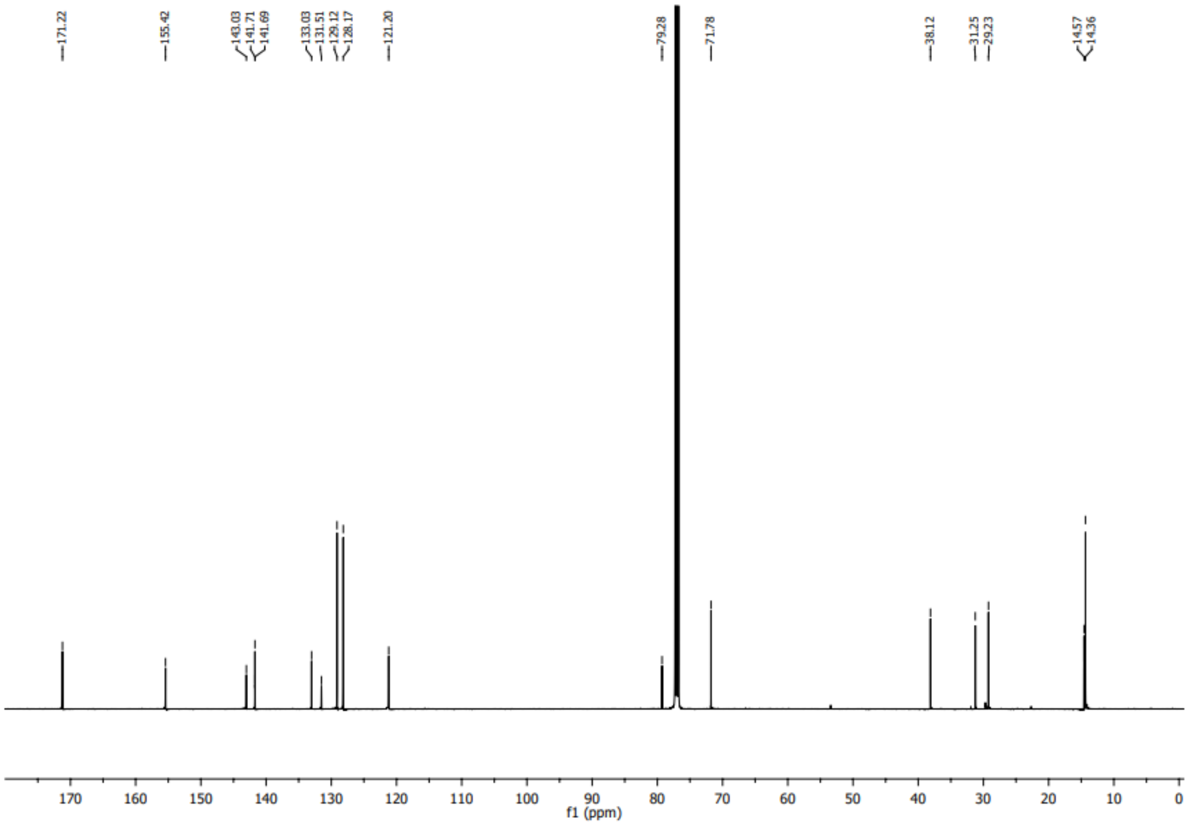

Compound **4** ($CDCl_3$)

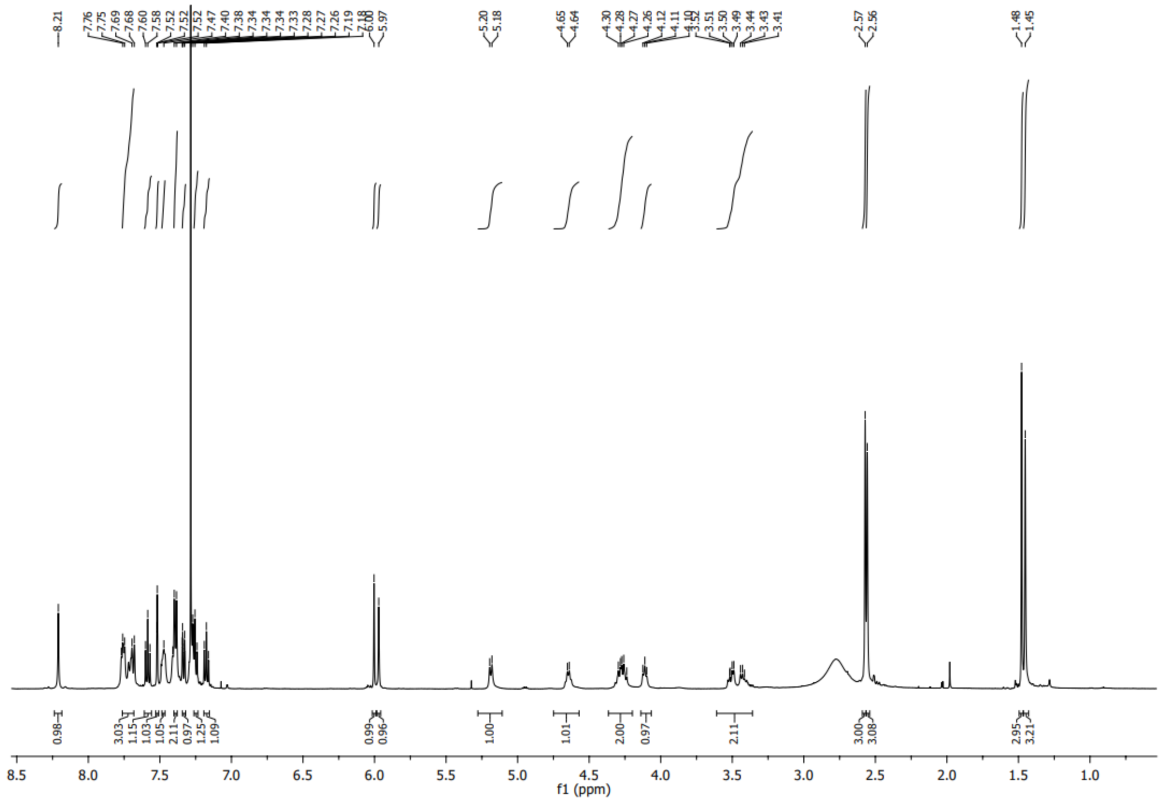

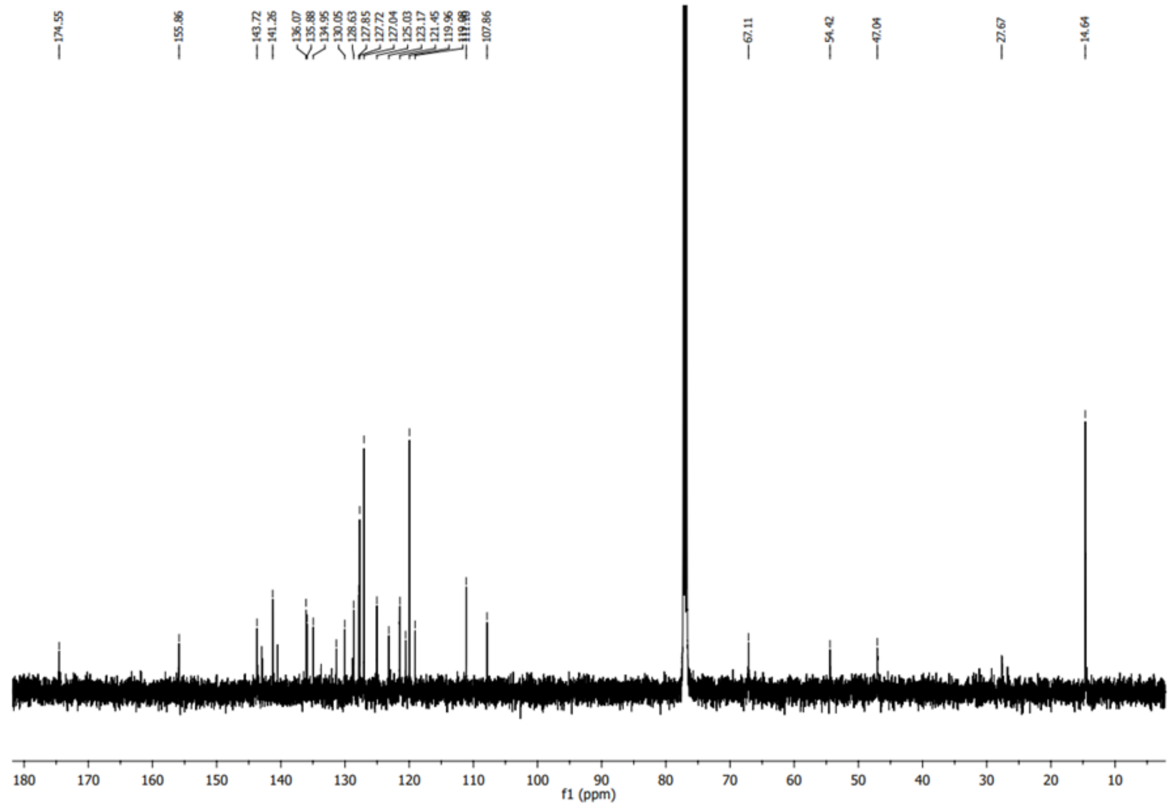

Compound **5** ($CDCl_3$)

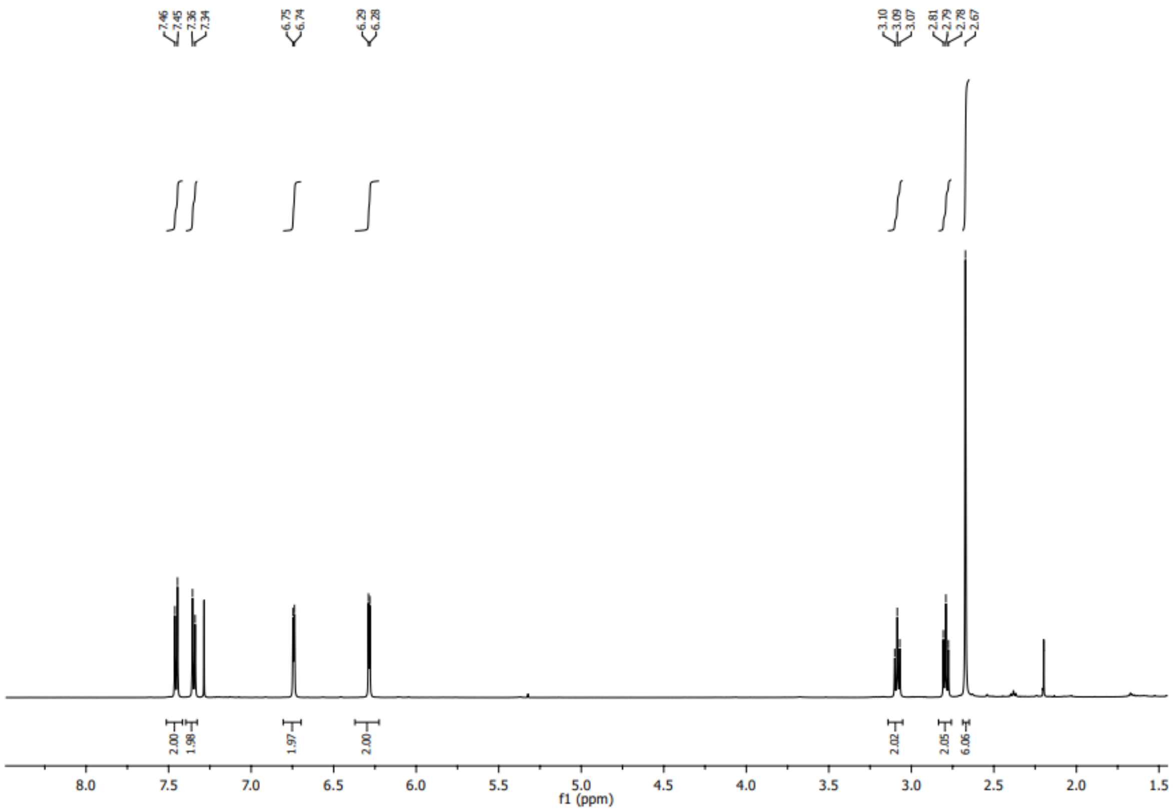

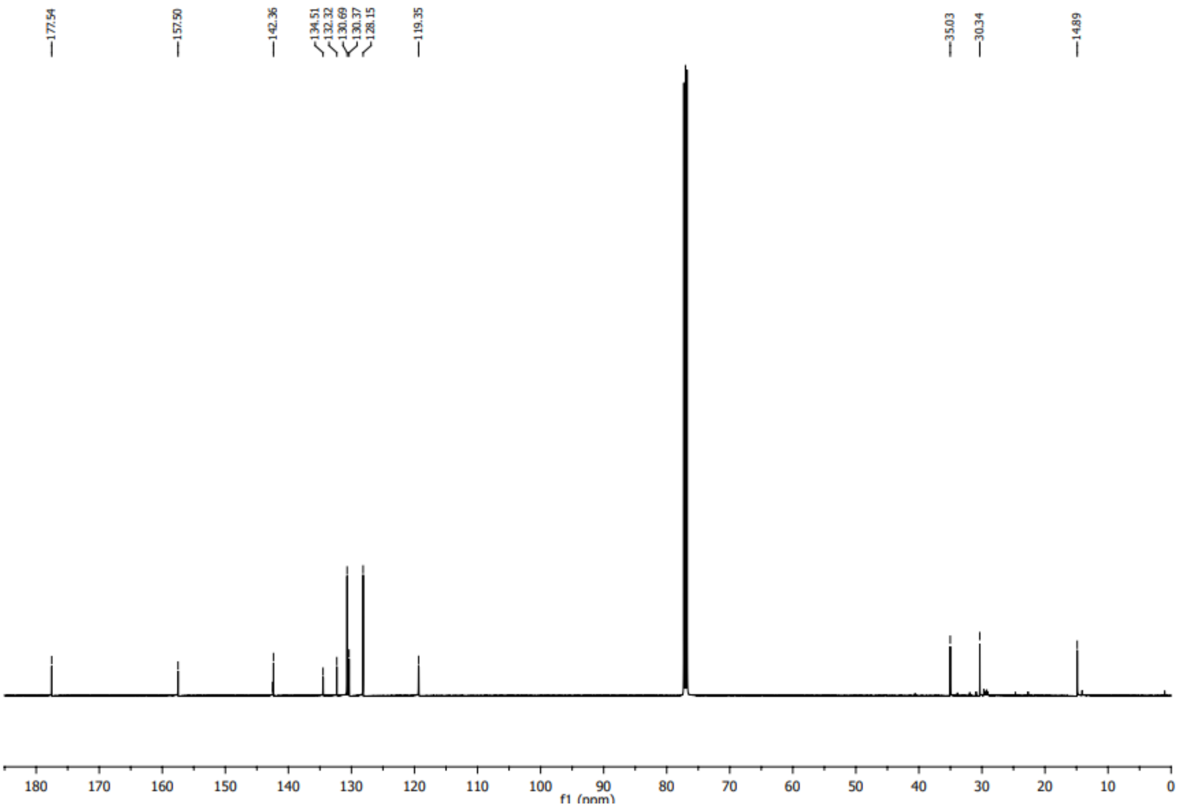

Compound **6** ($CDCl_3$)

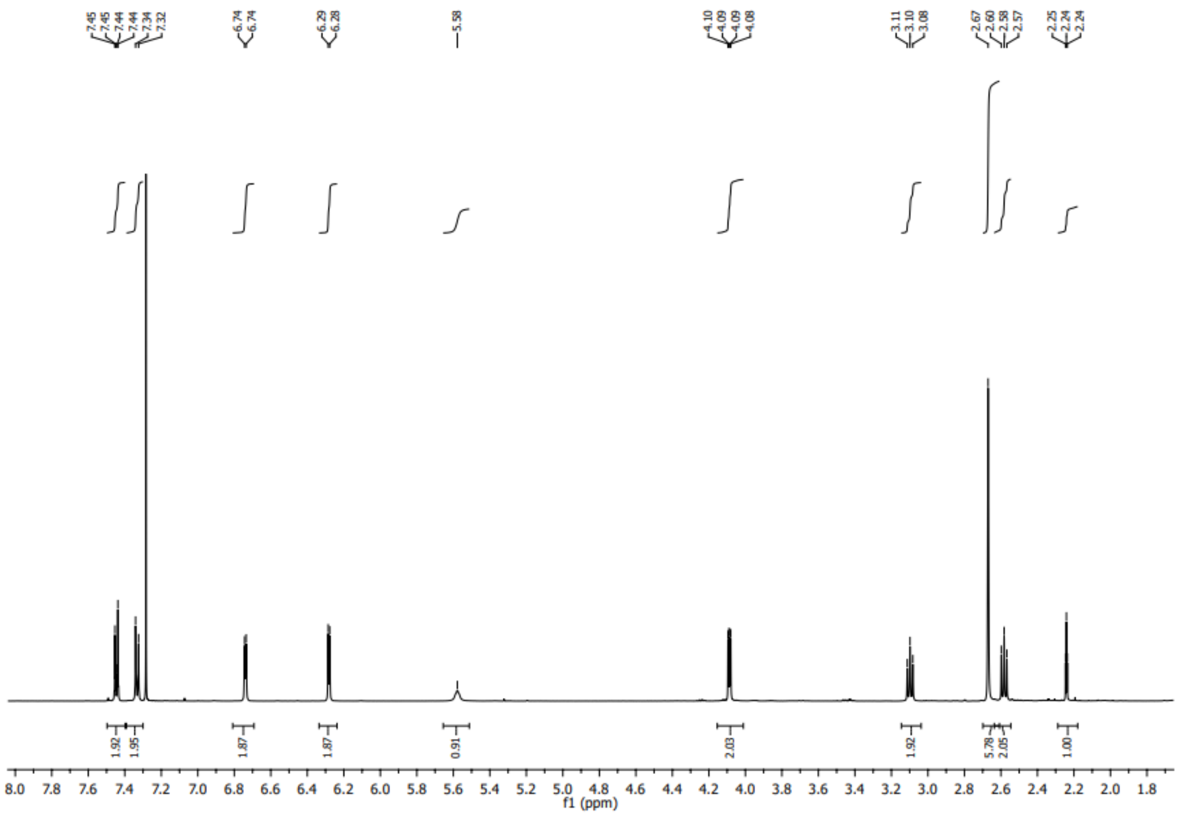

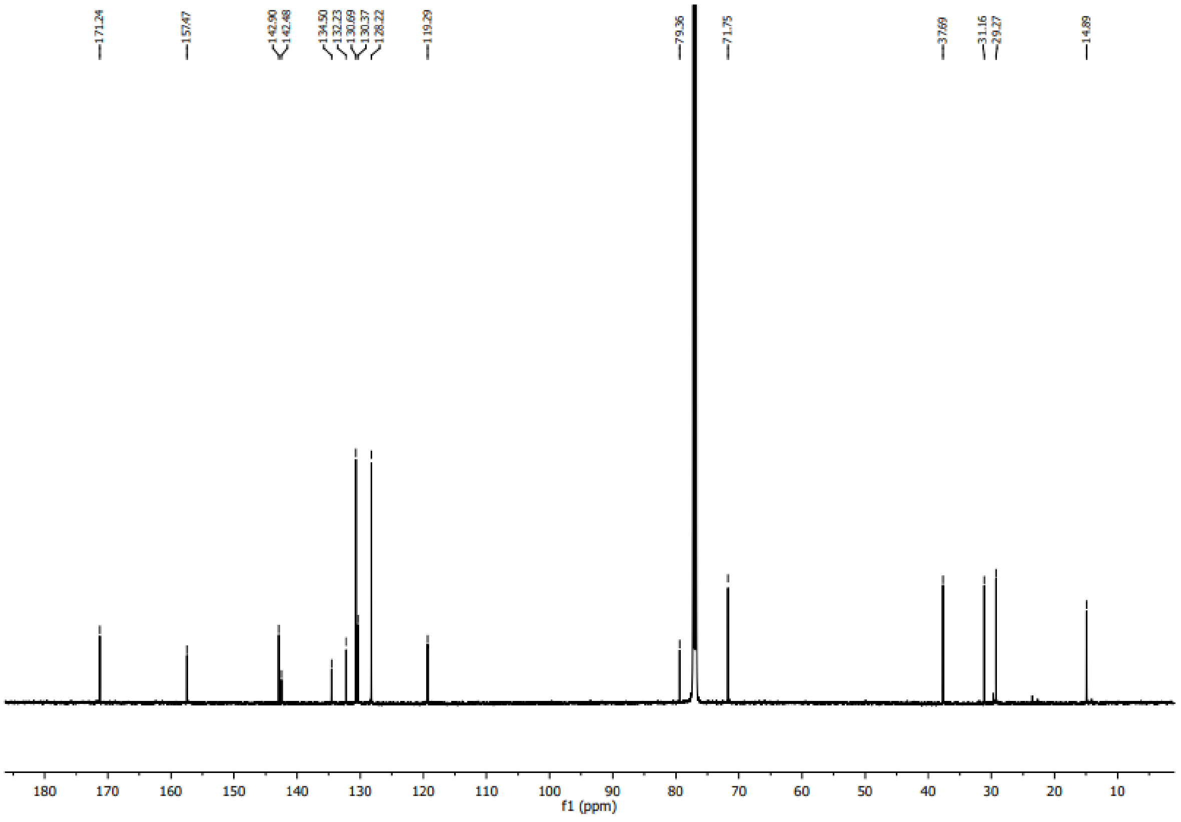

Compound **7** ($CDCl_3$)

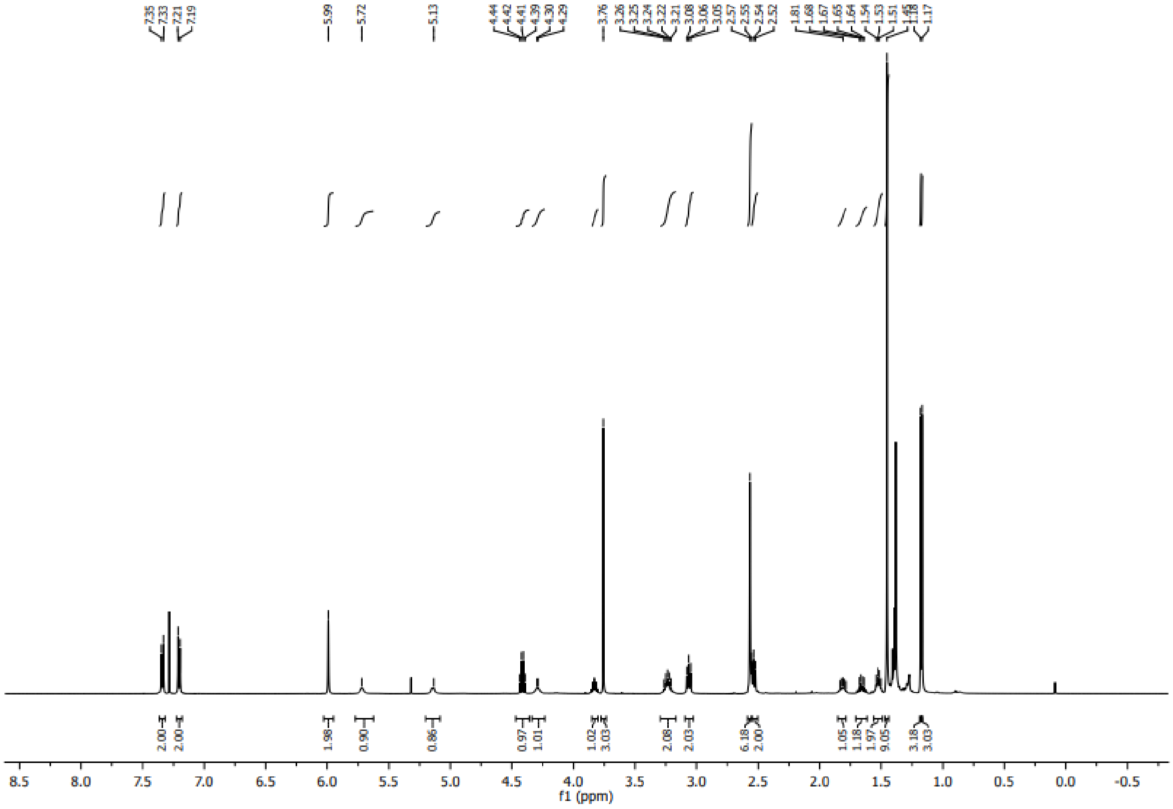

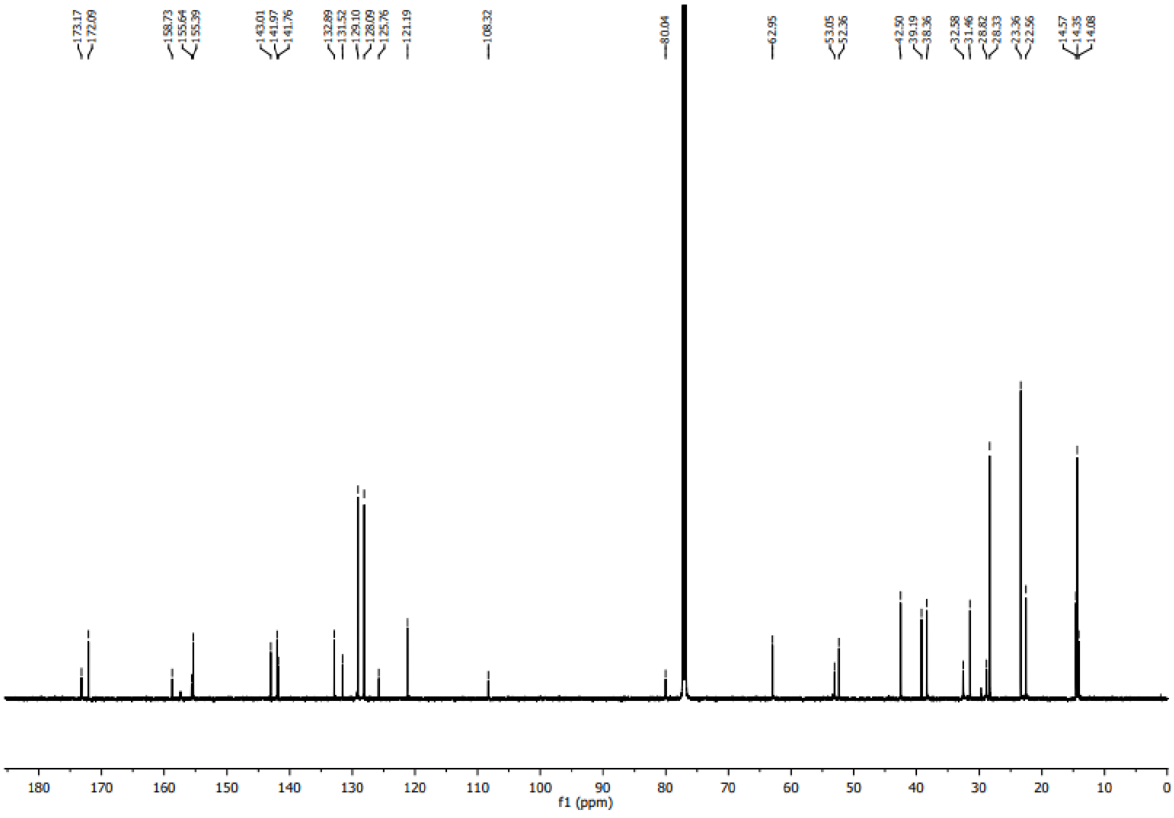

Compound **8** ($CDCl_3$)

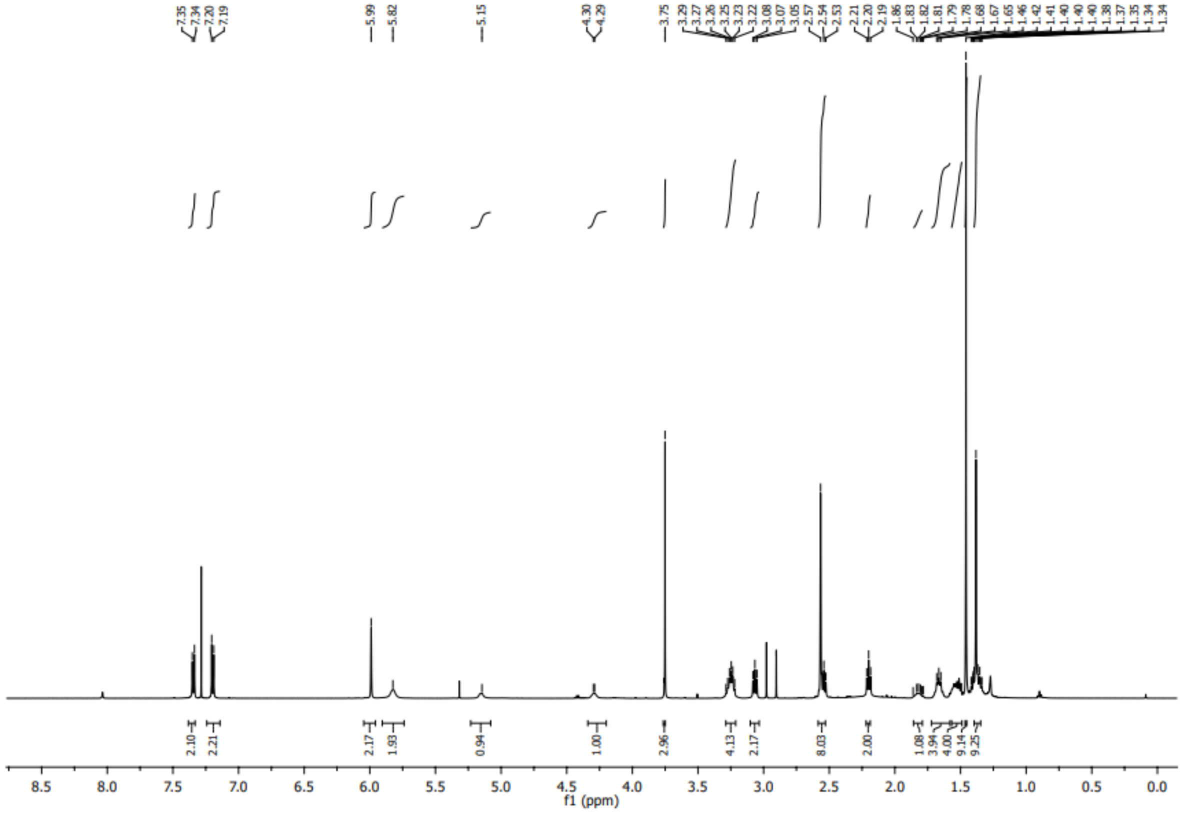

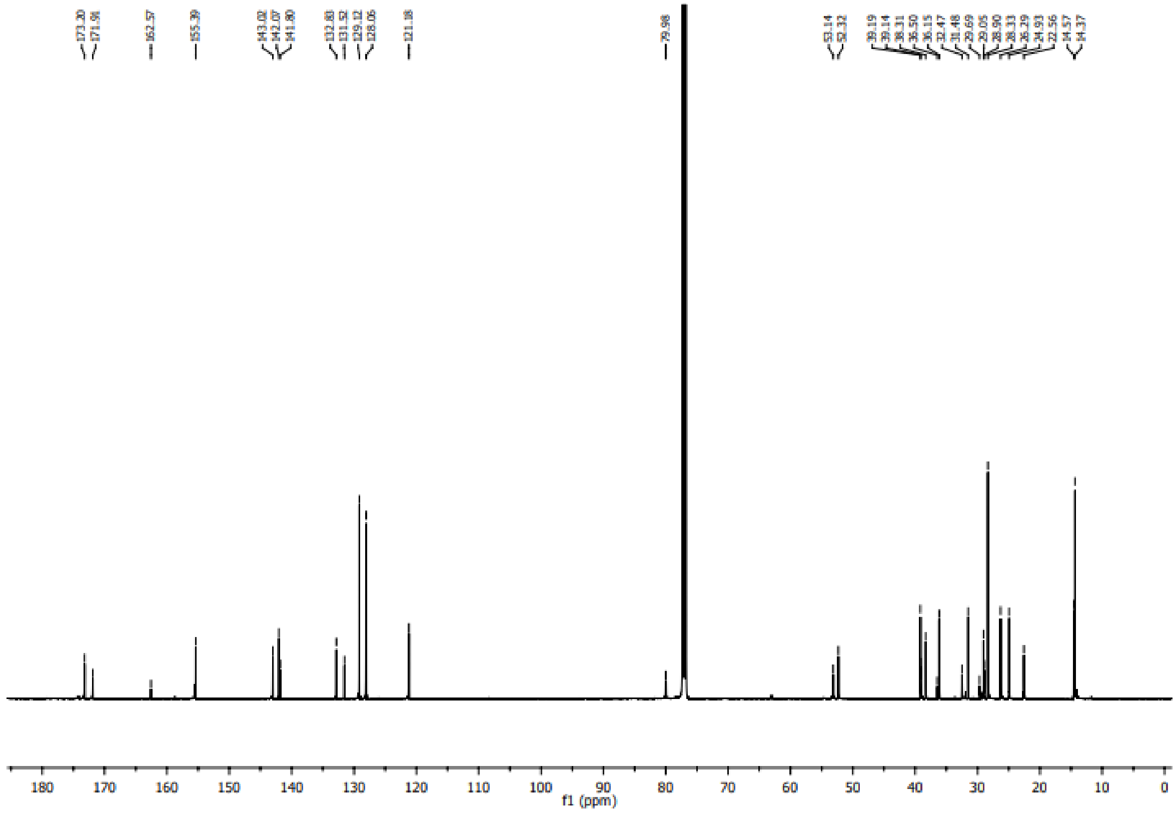

Compound **9** ($CDCl_3$)

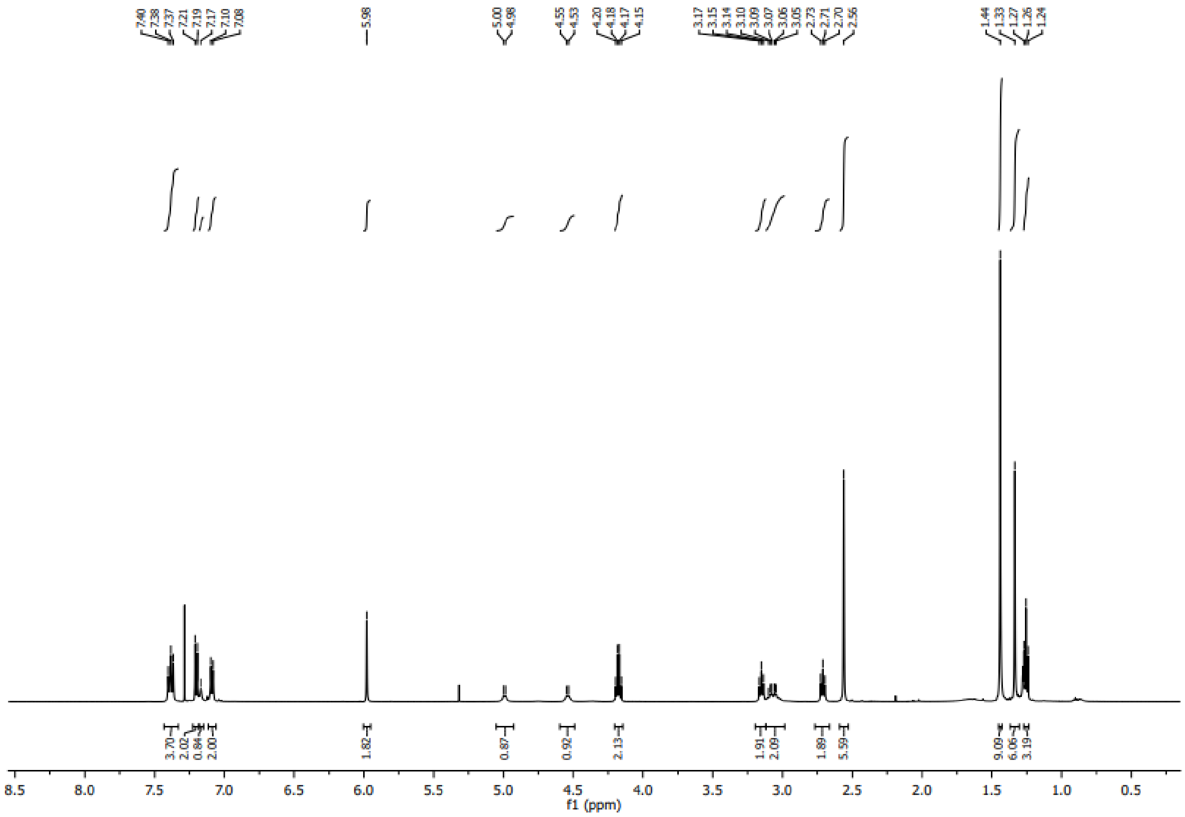

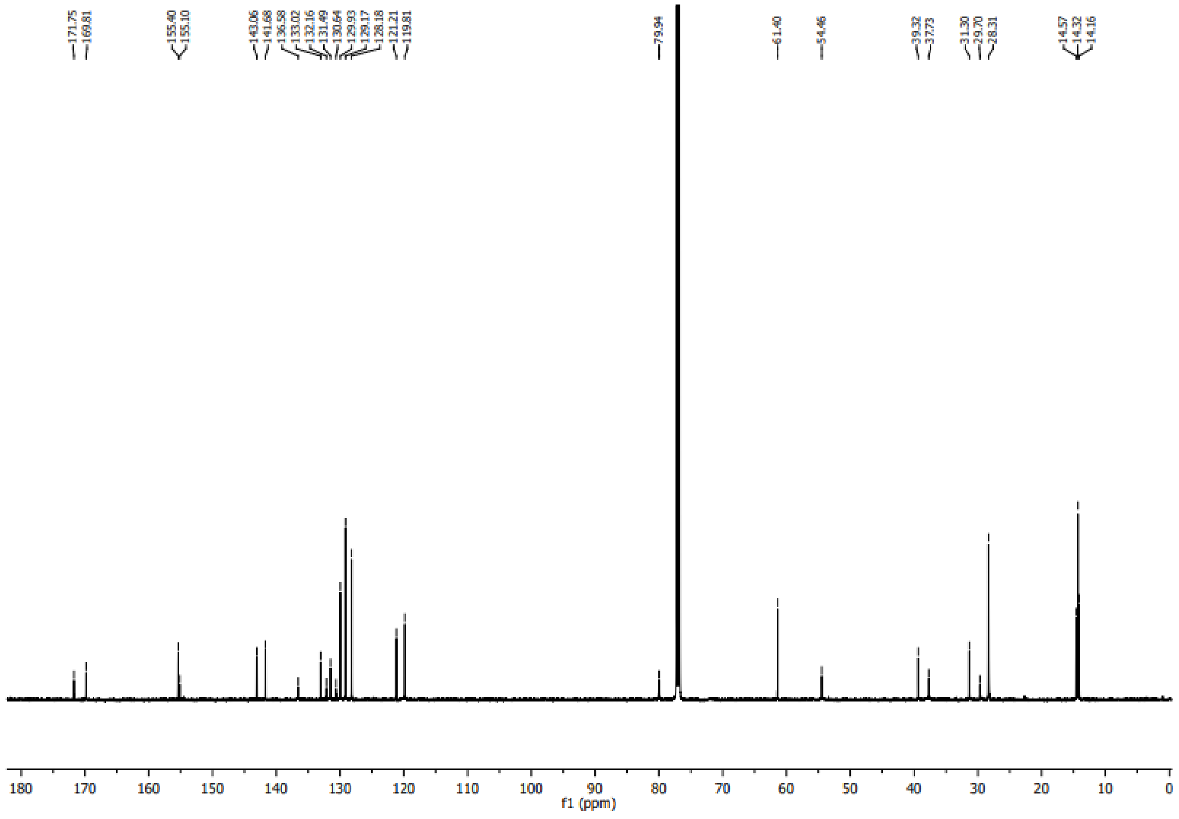

Compound **10** ($CDCl_3$)

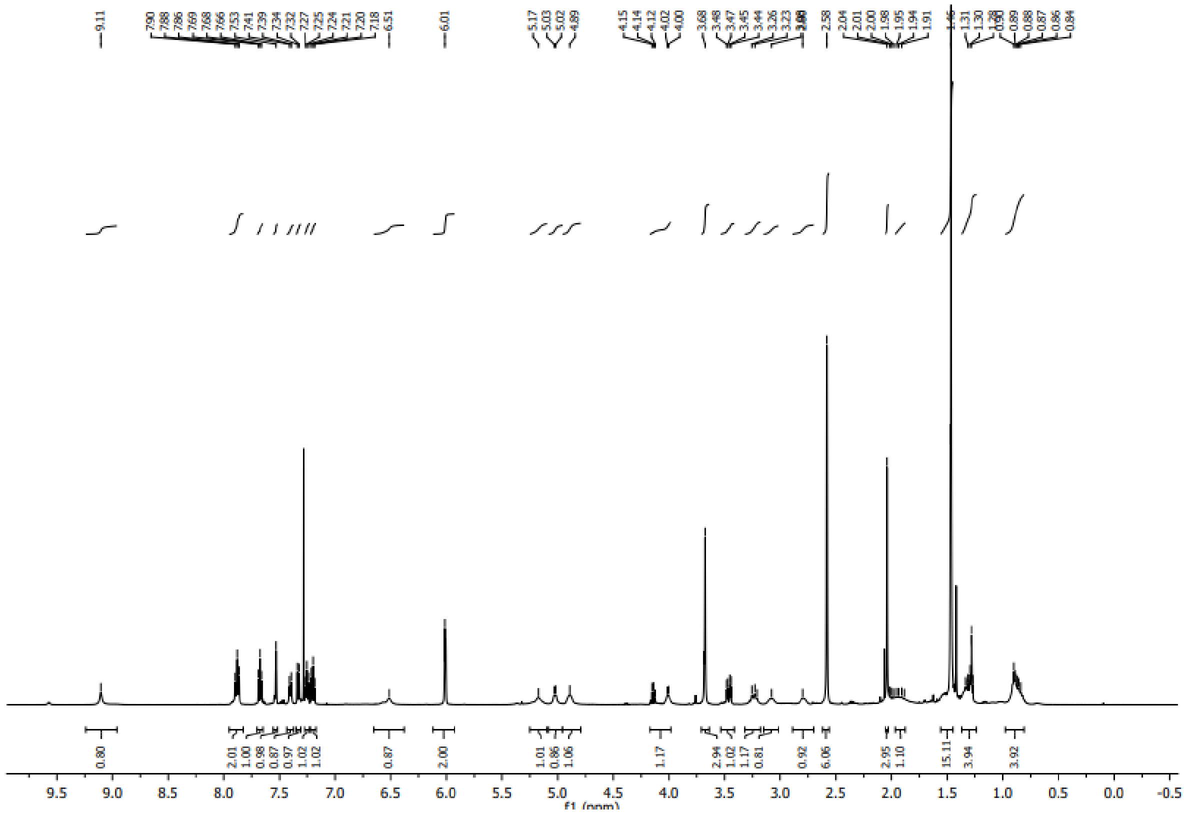

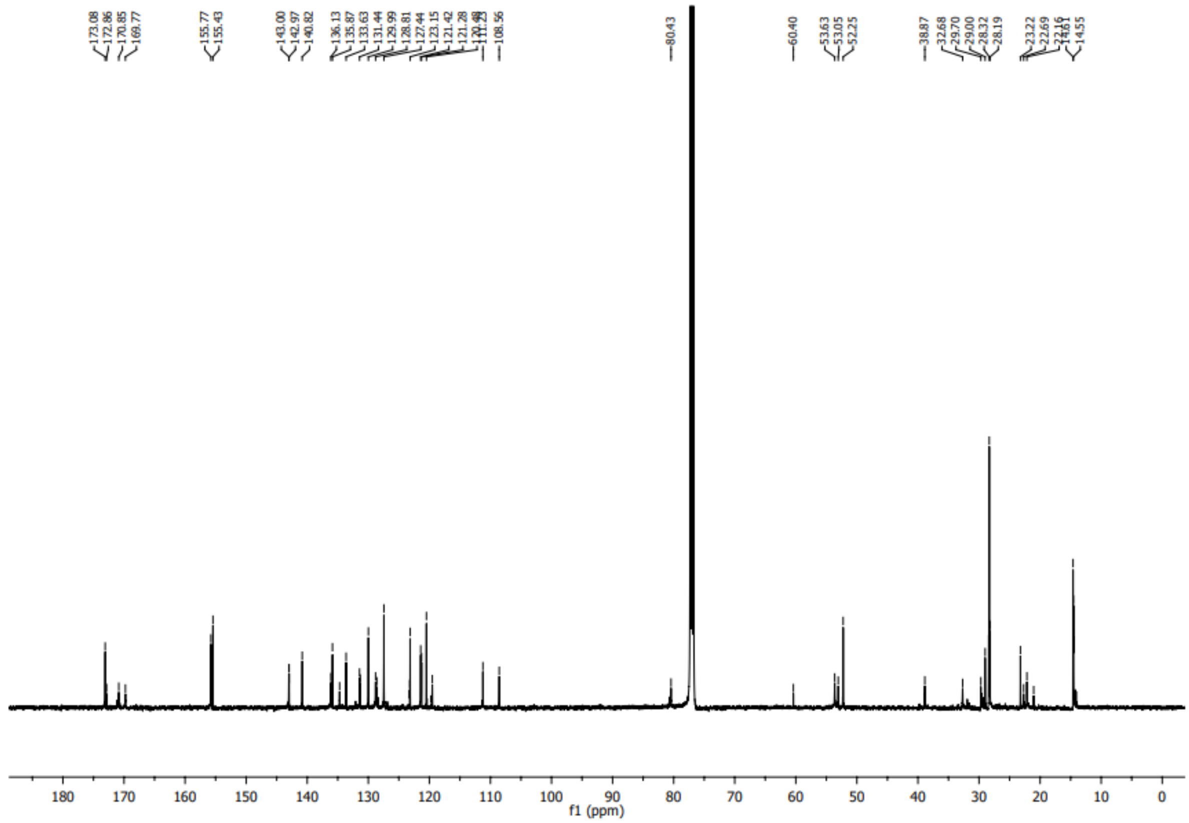

Compound **11** ($CDCl_3$)

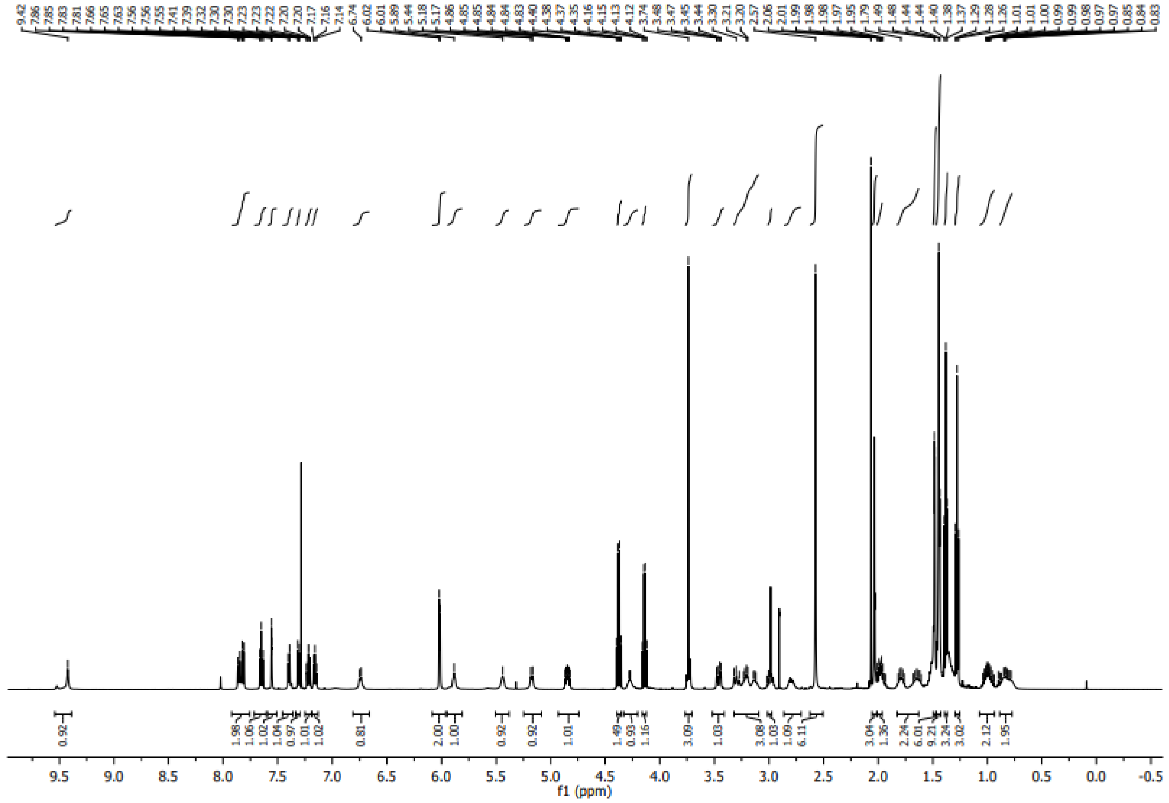

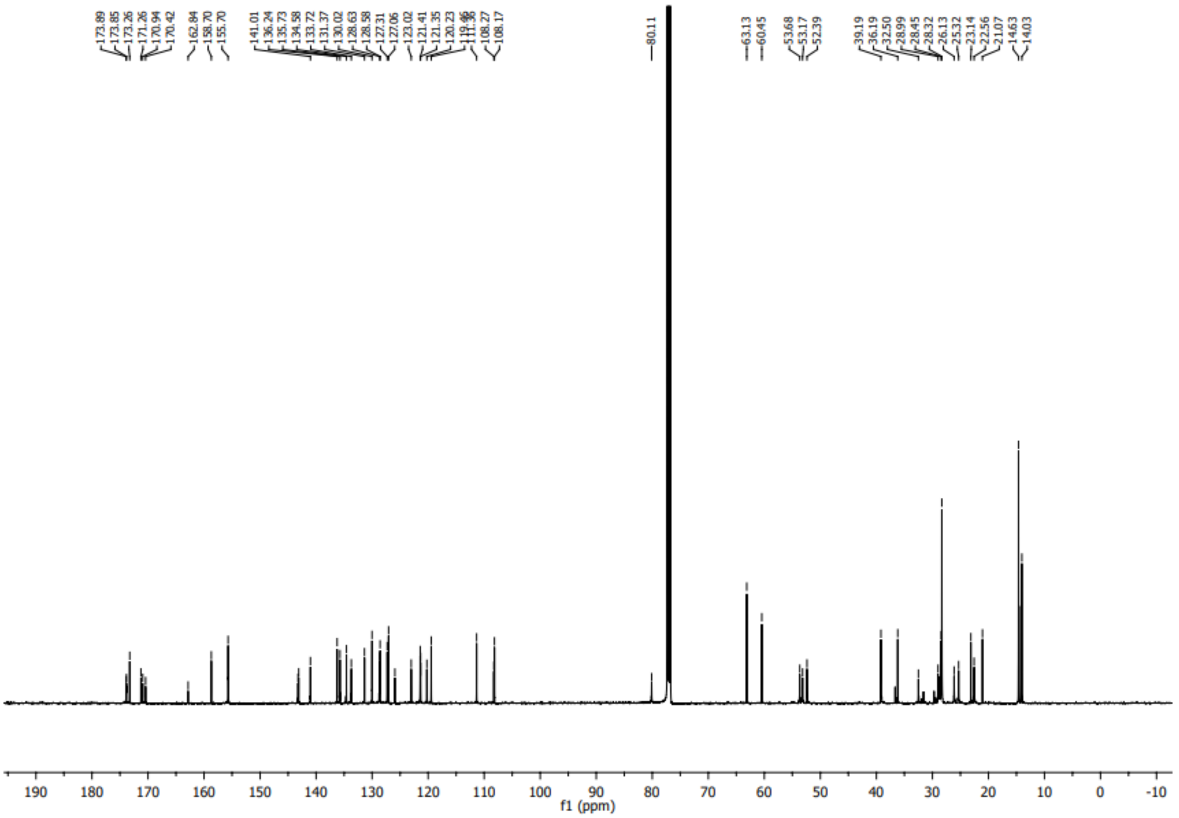

Compound **12** (MeOD)

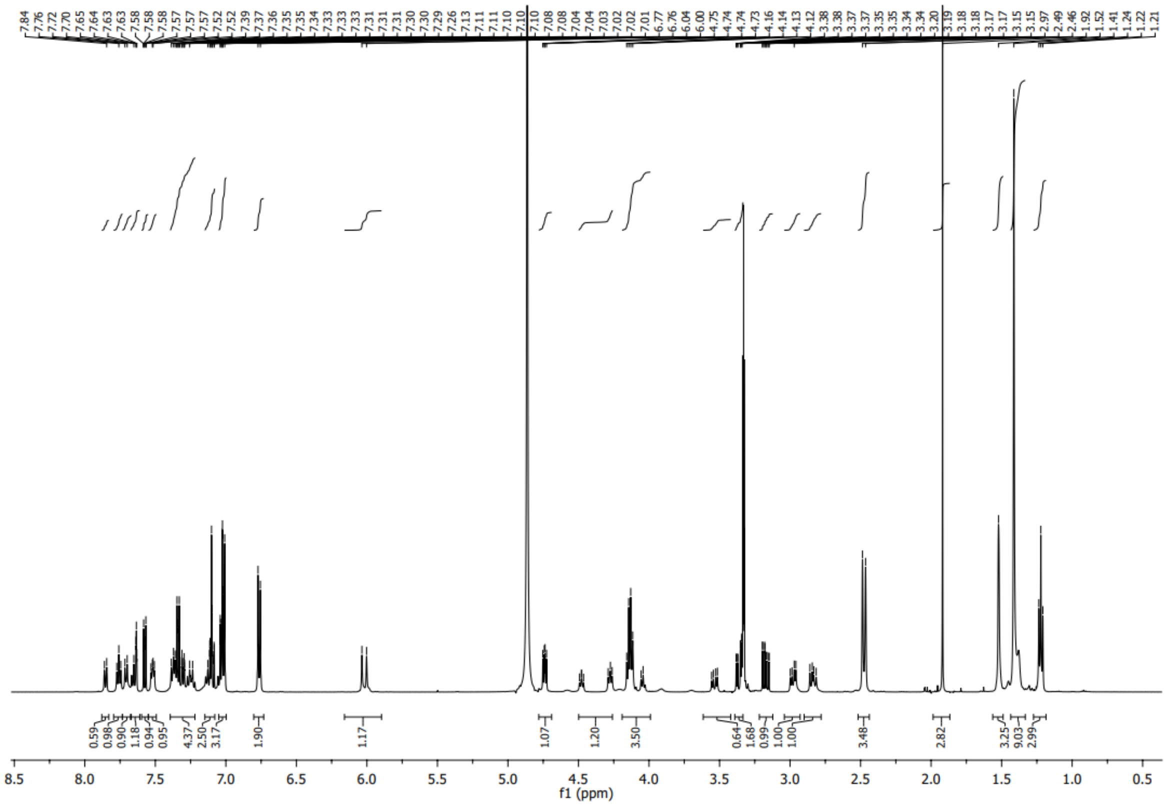

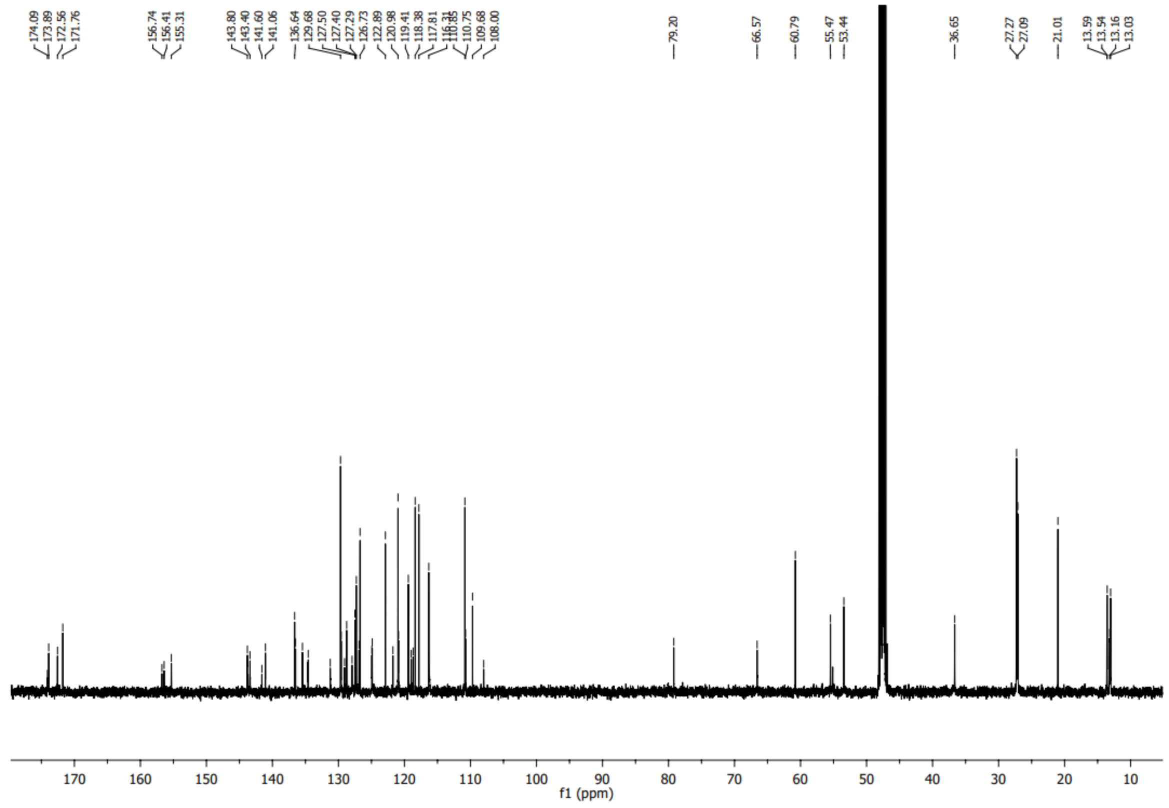

Compound **13** ($CDCl_3$)

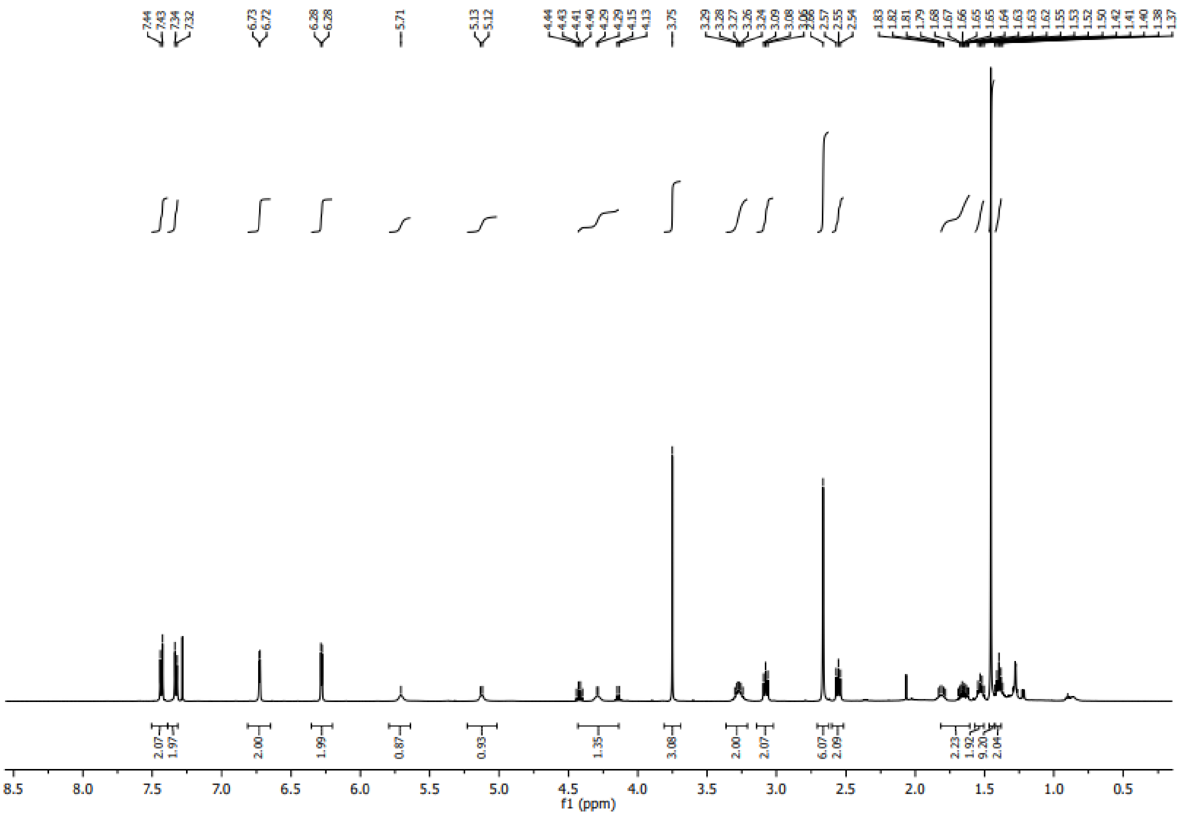

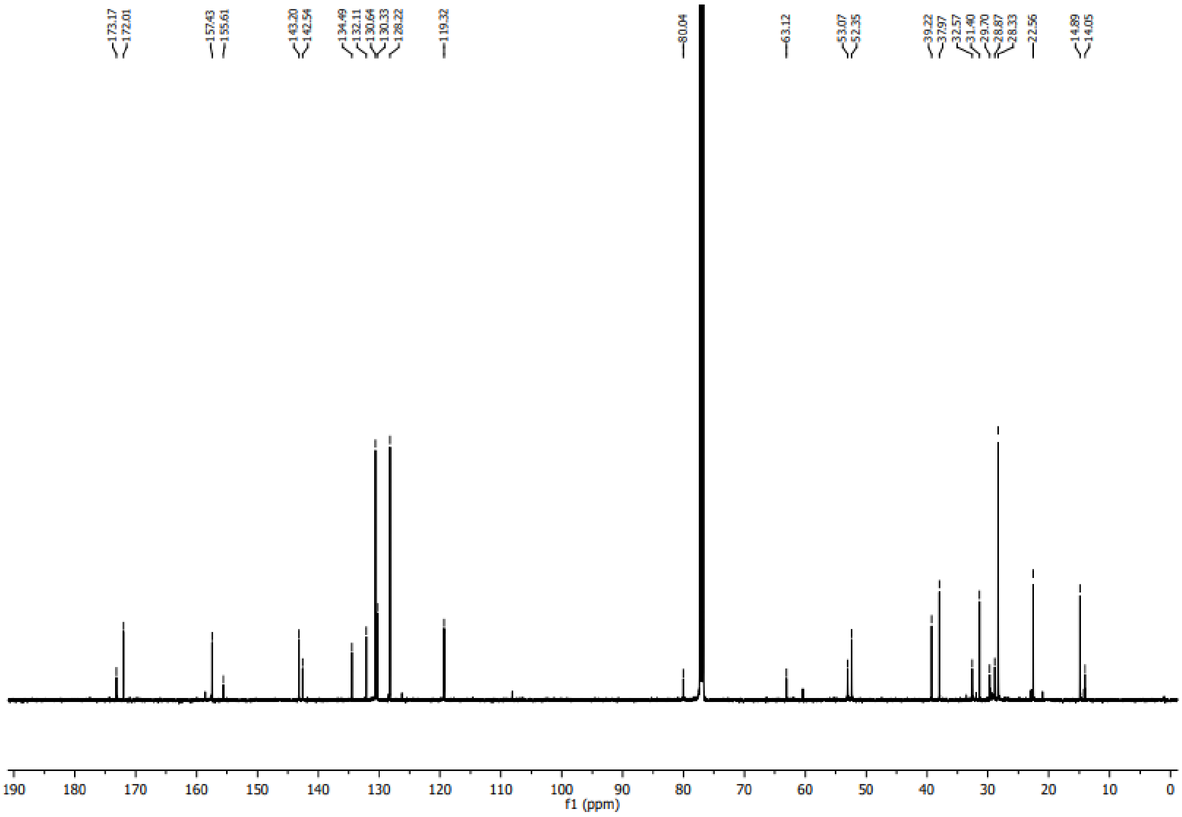

Compound **14** ($CDCl_3$)

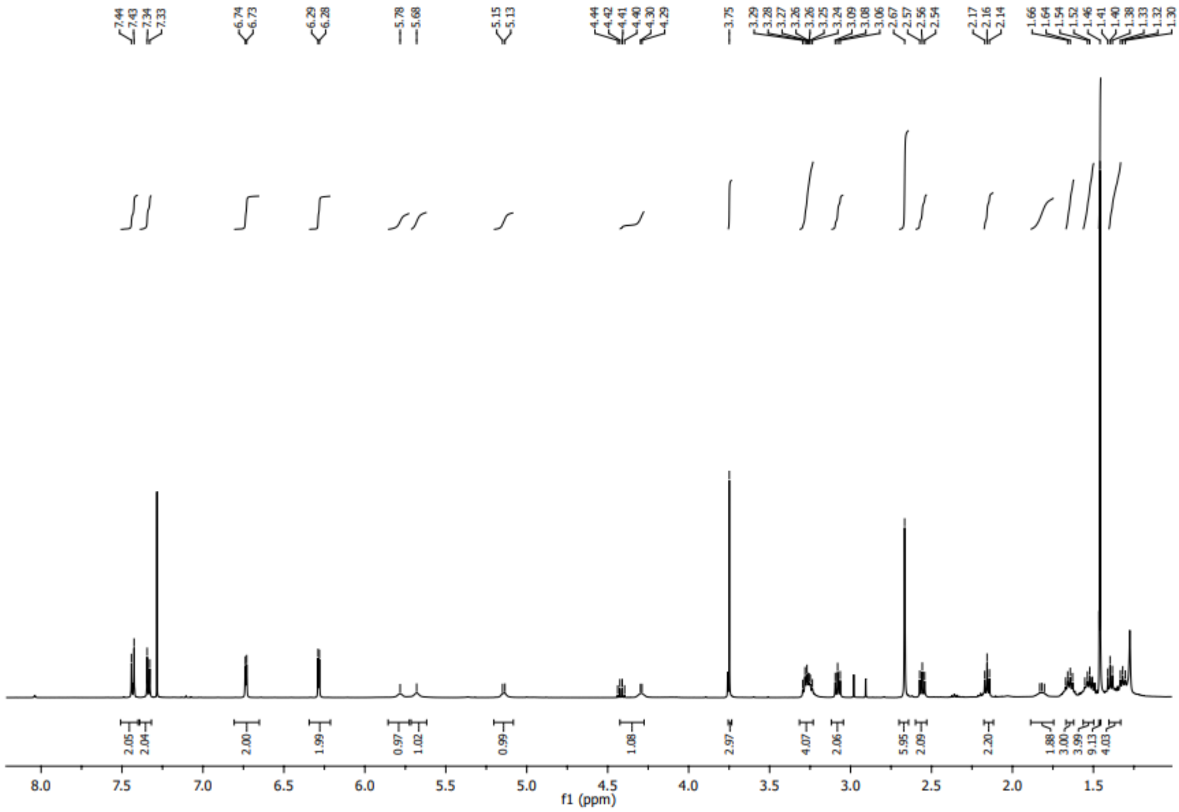

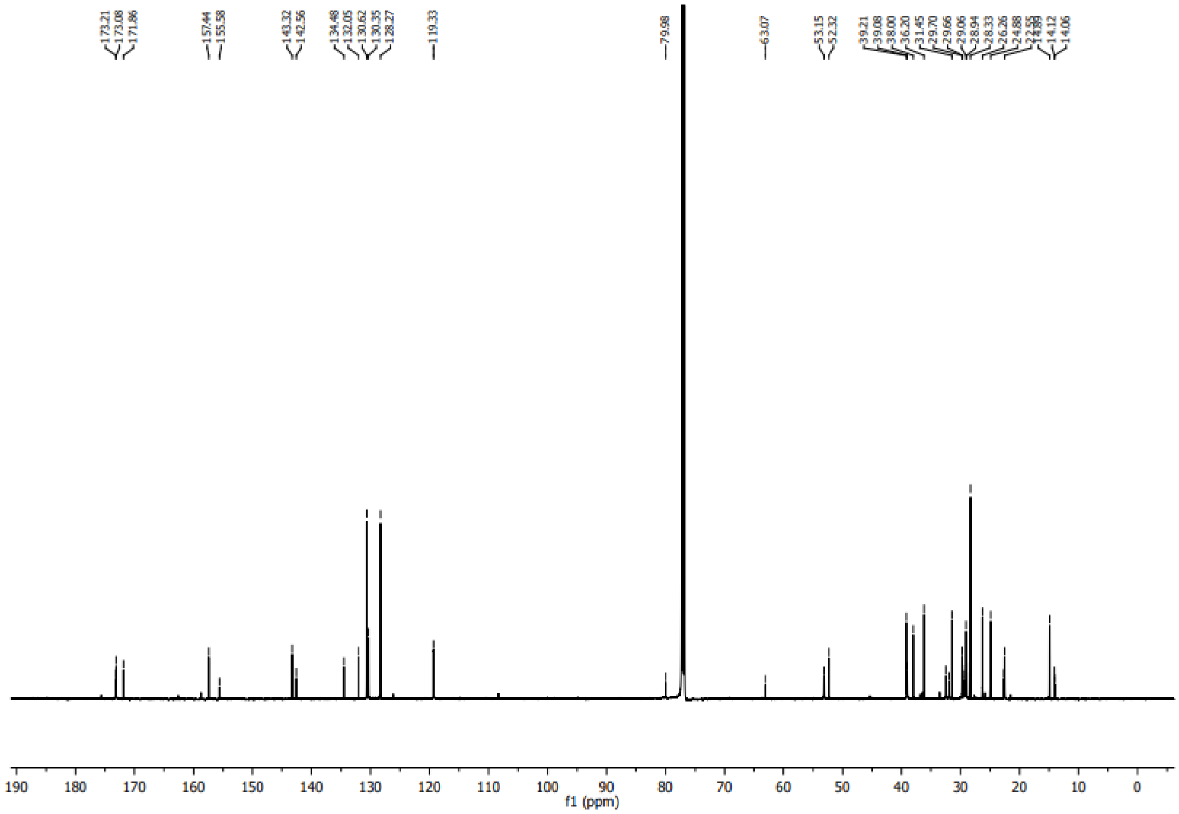

Compound 15 ($CDCl_3$)

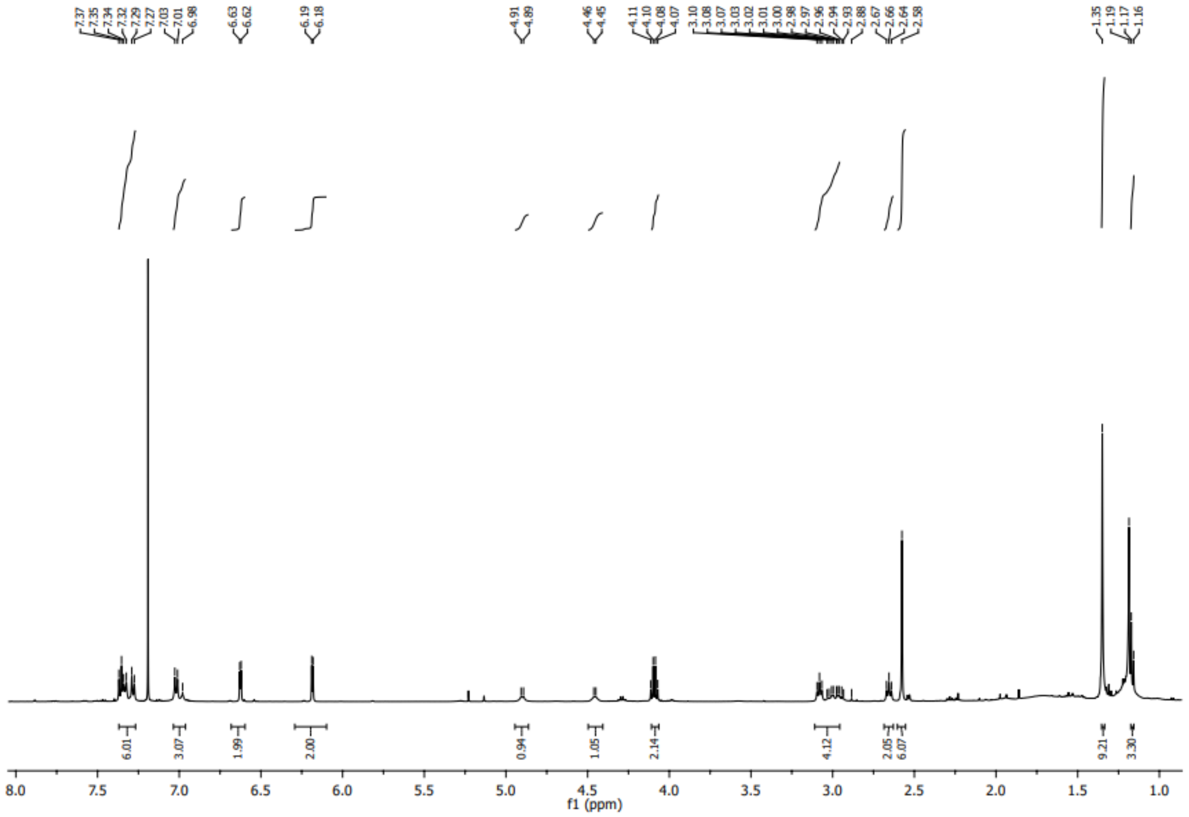

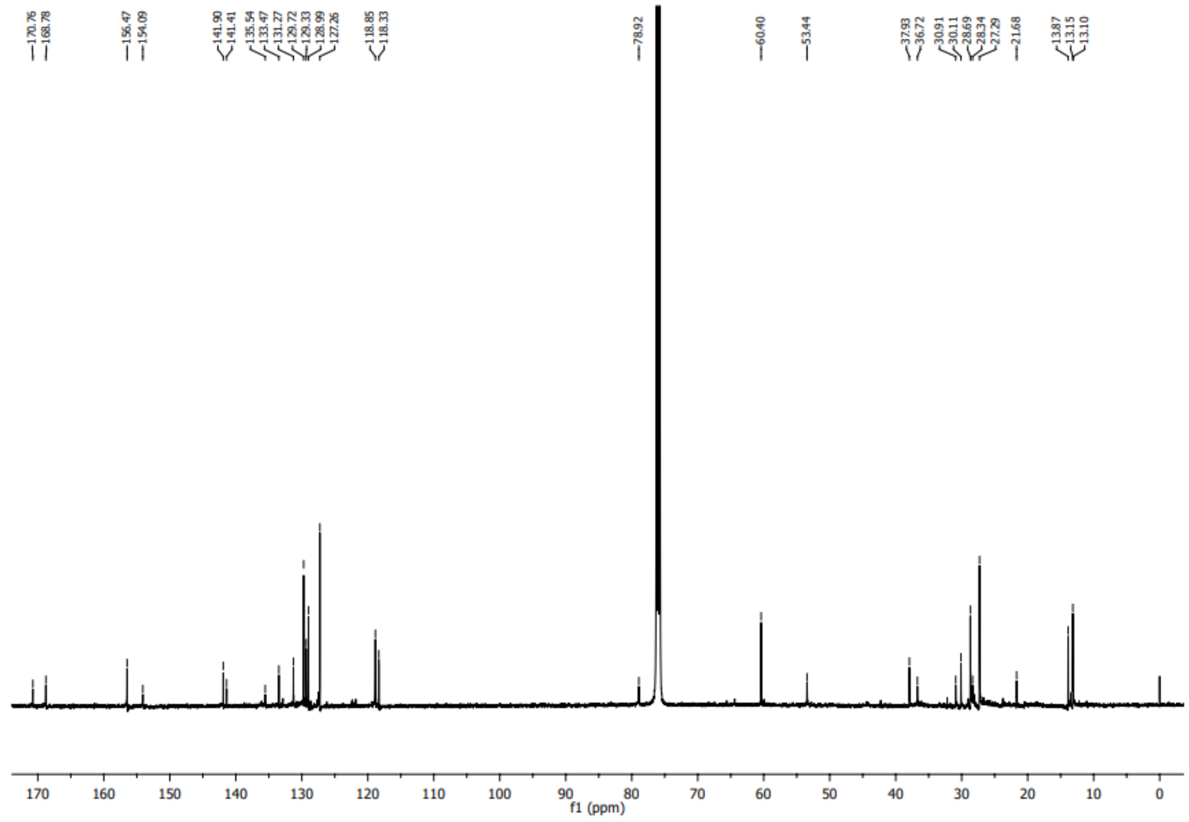

References

1. Bridge, T. et al. Site-specific encoding of photoactivity in antibodies enables light-mediated antibody–antigen binding on live cells. *Angew. Chem. Int. Ed.* **2019**, *48*, 17986–17993 (2019).
2. Bridge, T., Sachdeva, A. Engineering Homogeneous Photoactive Antibody Fragments. In: Tsai, YH., Elsässer, S.J. (eds) Genetically Incorporated Non-Canonical Amino Acids. *Methods in Molecular Biology*, vol 2676. Humana, New York, NY. https://doi.org/10.1007/978-1-0716-3251-2_2 (2023).
3. Osbourn, M. et al. HpARI Protein secreted by a helminth parasite suppresses interleukin-33. *Immunity* **2017**, *47*, 739–751.
4. Vacca, F. et al. A helminth-derived suppressor of ST2 blocks allergic responses. *Elife* **2020**, *9*, e54017.
5. Mendive-Tapia, L. et al. Spacer-free BODIPY fluorogens in antimicrobial peptides for direct imaging of fungal infection in human tissue. *Nat. Commun.* **2016**, *9*, 10940.
6. Lee, J. *et al.* Expanding the limits of the second genetic code with ribozymes. *Nat. Commun.* **2019**, *10*, 5097.
