# Supplementary material for: Inserting “OFF-to-ON” BODIPY Tags into Cytokines: A Fluorogenic Interleukin IL-33 for Real-Time Imaging of Immune Cells
Source: ACS Cent Sci. 2023 Dec 20;10(1):143–54. doi: 10.1021/acscentsci.3c01125 (PMC10823590; doi:10.1021/acscentsci.3c01125)
Supplement: Supplementary file 4 — oc3c01125_si_002.pdf [file oc3c01125_si_002.pdf]

oc-2023-01125n.R1

Name: Peer Review Information for "Inserting ‘OFF-to-ON’ BODIPY tags into cytokines: a fluorogenic interleukin IL-33 for real-time imaging of immune cells"

First Round of Reviewer Comments

Reviewer: 1

Comments to the Author

I have had the pleasure of reviewing 'Inserting ‘OFF-to-ON’ BODIPY tags into cytokines: a

fluorogenic interleukin IL-33 for real-time imaging of

immune cells' and found it to be a manuscript that is in principle suitable for publication in ACSCS, but will require some additional experiments and controls, as well as the inclusion of some additional key experimental data and procedures in the SI. Namely, the following:

- The title does not cover the content. Whereas in principle the approach could be used to image an immune cell, I would hardly class a HEK293TBlue-IL33 an immune cell. The over-expression of the ST2 and IL-1RAcP in this cell make it a mere model system. I do think that testing the approach in an actual immune cell expressing the ST2/-receptor at native, rather than over-expression levels, and in cells that have the correct machinery for activation and uptake of IL-33.

- I was also a little confused in the narrative by the heavy focus on the use of ivtt for the incorporation of the bodipy-UAAs. Whereas I find this a valuable exploration of this biochemical space, it did jar a little in the narrative, that the authors suddenly switch to a click based approach. A clearer rationale, and perhaps a shortening/move to the supplementary of the IVTT-part of the work would benefit the flow of the text.

- The final main point I have regarding the text relates to figure 5 and S17. Here the no-wash imaging of the IL-33-fluorogens is presented. What I could not get clear, as there is no description of the washing experiment in the experimental is how washing, of what I assume are live cells, can lead to signal loss. Are these very long washing steps that allow for exocytosis? Or are the cells permeabilised to lead to loss of protein. The hypothesis posed by the authors that it is unbound IL33 does not quite hold for me, as the taken-up IL33 would still be retained inside an endosome during this washing. A clearer description of this washing experiment is essential as I am not sure that these experiments support the hypothesis in this format.

- I also miss a co-localisation study to confirm the proposed lysosomal localisation.

- I also miss the control experiment where a non-IL-33 HEK cell is used to distinguish background uptake.

- The final issue I have is that the relatively modest turn-on has made it a bit of a 'goldilocks'-experiment, where the conditions need to be 'just right' to get an image. Have the researchers perhaps performed the uptake assays at different concentrations, so that a sense can be given of the concentration range (and laser/detector setting range) over which these probes can be used?

I have some additional smaller points:

- There is no description of the IL-33 ELISA experiment

- It is unclear why the authors use oNB-IL-33 in figure 4c for their ELISA assay and not the Bodipy analogues

- I miss a discussion (and even a mention) of protein expression yields, and obtained purities. I think it woul be very valuable to have the isolated yields of modified proteins per liter culture included in the SI.

- The movies do not play on my computer and could therefore not be evaluatedd.

- The mass spectra in figure S20 are only the deconvoluted mass spectra. Inclusion of the full unprocessed LC-traces and ion envelopes, as well as an indiciation which part of the ion envelopes were used for the deconvolution are essential for assessing the purity (for example, by inclusion of a coommassie-stained gel in Figure S16).

-Page S23 has a 50 mg and a 200 mg/mL, which should maybe be replaced by a micro-sign.

Again, once these comments are addressed, I think the manuscript would fit very well within ACSCS.

Reviewer: 2

Comments to the Author

The authors are presenting here a generic platform to site-specifically incorporate small BODIPY tags into cytokine proteins (although really any proteins). The success of the manuscript is a pair of propargylated BODIPYs that can be clicked onto azidoPhe in proteins. These labelled cytokines showed native activity profiles and could be imaged with wash-free fluorescence microscopy. This is a neat story in itself. The authors also provide another story of preparing BODIPY-bearing amino acids, which can be ligated to different tRNA constructs, but are not successfully incorporated by ribosomal synthesis. This story does not fit with the rest of the discussion, and I suggest would be better published separately. I believe there is great value in publishing negative results such as this, and indeed this work can be incorporated into a story that confirms that other BODIPY amino caids of the size of BODIPY-FL can be incorporated, for example.

My other suggestion for improving the manuscript is a clearer explanation/ discussion of the definition of fluorogenic here: it is not fluorogenic upon clicking to the protein, but rather in a more lipophilic

environment. This should be spelled out more clearly, and the advantages enumerated. What if the protein environment being labelled is particularly hydrophilic?

Author's Response to Peer Review Comments:

Formatting needs

SI PARAGRAPH: If the manuscript is accompanied by any supporting information for publication, a brief description of the supplementary material is required in the manuscript. The appropriate format is: Supporting Information. Brief statement in non-sentence format listing the contents of the material supplied as Supporting Information.

Answer: Addressed in the revised manuscript.

GENERAL REF FORMATTING: Periodical references should contain authors' surnames followed by initials, article title, journal abbreviation, year, volume number, and page range. Refs with more than 10 authors should list the first 10 and then be followed by "et al." Web sources must include access date.

Answer: Addressed in the revised manuscript.

TOC MISSING: Provide a TOC image per journal guidelines (3.25 in. × 1.75 in. (8.25 cm × 4.45 cm); on the last page of the Manuscript) with the heading "TOC Graphic" above the graphic. Make sure to designate the file as "Graphic for Manuscript."

Answer: Addressed in the revised manuscript.

SYNOPSIS MISSING: The synopsis should be no more than 200 characters (including spaces) and should reasonably correlate with the TOC graphic. The synopsis is intended to explain the importance of the article to a broader readership across the sciences. Please place your synopsis in the manuscript file after the TOC graphic, and label it as "Synopsis."

Answer: Addressed in the revised manuscript.

SI HEADER: The supporting information should be formatted with a cover sheet listing authors, author affiliations, corresponding author email, manuscript title, and the number of pages, figures, and tables. The Author affiliations must match the MS.

Answer: Addressed in the revised manuscript.

Reviewer 1

1. *The title does not cover the content. Whereas in principle the approach could be used to image an immune cell, I would hardly class a HEK293TBlue-IL33 an immune cell. The overexpression of the ST2 and IL-1RAcP in this cell make it a mere model system. I do think that testing the approach in an actual immune cell expressing the ST2/-receptor at native, rather than over-expression levels, and in cells that have the correct machinery for activation and uptake of IL-33.*

Answer: We thank the reviewer for the comment. We agree with the reviewer that HEK293T cells are a model system, and we further analyzed the uptake of IL33(**6**) in HMC1.1 cells. HMC1.1 cells are a human mast cell line that can be stimulated by IL-33 (Inflamm. Res. 2010, 59, 207-218, new reference 78) and therefore a suitable system to test the behaviour of our constructs in human immune cells with native levels of ST2 receptors. Specifically, we performed fluorescence microscopy and flow cytometry experiments where we demonstrated the specific uptake of IL-33(**6**) via ST2 receptors by titration with increasing concentrations of the fluorogenic protein and competition experiments with the ST2 inhibitor HpBARI. These new results are included in the revised manuscript (revised Figure 5 and new Figure S20).

2. *I was also a little confused in the narrative by the heavy focus on the use of ivtt for the incorporation of the bodipy-UAAs. Whereas I find this a valuable exploration of this biochemical space, it did jar a little in the narrative, that the authors suddenly switch to a click based approach. A clearer rationale, and perhaps a shortening/move to the supplementary of the IVTT-part of the work would benefit the flow of the text.*

Answer: We thank the reviewer for the observation. Our previous version of the manuscript included several experiments to explore the chemical diversification around BODIPY-UAAs as well as their evaluation for protein incorporation via different strategies (from flexizymes to chemical amino acylation). Importantly, some of the presented results -despite negative- supported important conclusions, for instance, the preferential incorporation of small-sized BODIPY structures (e.g., BODIPY-FL) over larger hydrophobic amino acids (Trp-BODIPY). However, given that this observation is also highlighted by reviewer 2, we decided to remove entirely from the main text all the results and discussion around chemical derivatization of BODIPYs for flexizyme-mediated ligation and subsequent assays with dFx and eFx. These are now included as a Supplementary Discussion in the Supporting Information.

3. *The final main point I have regarding the text relates to figure 5 and S17. Here the no-wash imaging of the IL-33-fluorogens is presented. What I could not get clear, as there is no description of the washing experiment in the experimental is how washing, of what I assume*

are live cells, can lead to signal loss. Are these very long washing steps that allow for exocytosis? Or are the cells permeabilised to lead to loss of protein. The hypothesis posed by the authors that it is unbound IL33 does not quite hold for me, as the taken-up IL33 would still be retained inside an endosome during this washing. A clearer description of this washing experiment is essential as I am not sure that these experiments support the hypothesis in this format.

Answer: We thank the reviewer for the comment and apologise for the overlook. We have now included details of the washing step (one wash with 100 µL PBS) both in the text and in the legend of Figure 5. We have also clarified our observation that the similar fluorescence intensities before and after washing suggest that IL-33(**6**) can be an accurate reporter of ST2mediated internalization, which have been now confirmed with new experiments in nontransfected HEK293 cells where we observed complete lack of fluorescence signals (new Figure S17).

4. I also miss a co-localisation study to confirm the proposed lysosomal localisation.

Answer: We thank the reviewer for the comment. We have performed fluorescence microscopy experiments to analyze the co-localization of IL-33(**6**) with the lysosome marker Lysotracker. The results show partial co-localization of the two signals and are included in the new Figure S19.

5. I also miss the control experiment where a non-IL-33 HEK cell is used to distinguish background uptake.

Answer: We thank the reviewer for the comment. We have performed microscopy experiments to compare the uptake of IL-33(**6**) in transfected and non-transfected HEK293 cells. The fluorescence images show higher internalization of IL-33(**6**) in HEKBlue cells transfected with ST2 receptors and marginal signals in non-transfected HEK293 cells. These results are included in the new Figure S17.

6. The final issue I have is that the relatively modest turn-on has made it a bit of a 'goldilocks'experiment, where the conditions need to be 'just right' to get an image. Have the researchers perhaps performed the uptake assays at different concentrations, so that a sense can be given of the concentration range (and laser/detector setting range) over which these probes can be used?

Answer: To address this point, we have run new experiments where we have titrated the IL33(**6**) to determine the range of working concentrations that can be readily detected in ST2transfected HEKBlue cells using fluorescence microscopy. As shown in the new Figure S17, we observed bright, detectable signals when cells were incubated with IL-33(**6**) (1 µM and 300 nM). Furthermore, our new experiments in HMC1.1 cells also proved that IL-33(**6**) can be detected in other cell types and using different fluorescence-based assays (e.g., confocal microscopy, flow cytometry in Figures 5 and S20 respectively).

7. There is no description of the IL-33 ELISA experiment.

Answer: We apologise for the overlook, and we have now included a description of the IL-33 ELISA experiment in the Supplementary Information.

8. It is unclear why the authors use oNB-IL-33 in figure 4c for their ELISA assay and not the Bodipy analogues

Answer: We thank the reviewer for the comment. The ELISA experiments described in Figure 4c were designed to evaluate whether the replacement of Tyr residues 143 and 163 with a bulkier amino acid, such as pcY, was affecting ST2 binding and therefore identify an optimal site for fluorophore conjugation. Once we found that Tyr143 was a suitable residue for IL-33 derivatization, we prepared the fluorescent analogues IL-33(**1**), IL-33(**3**) and IL-33(**6**). Given that the main application of these fluorescent analogues was to image the intracellular trafficking of IL-33, we decided to perform SEAP functional assays -instead of ELISA assays- not only to confirm binding to ST2 by competition with the inhibitor HpBARI_Hom2 but also to examine downstream signaling through activation of the NF-κB and AP-1 pathways. The results in Figure 5 confirmed that all derivatives retained the ability to bind ST2 and cause downstream signalling to a similar extent as unlabeled IL-33.

9. I miss a discussion (and even a mention) of protein expression yields, and obtained purities. I think it woul be very valuable to have the isolated yields of modified proteins per liter culture included in the SI.

Answer: We thank the reviewer for the comment. We have included a table with the expression yields for the three labeled proteins using chemical amino acylation methods (i.e., IL33(**1**), IL-33(**3**) and IL-33(**6**), new Figure S15). We have also included in the revised text a specific mention to the isolated yields obtained in bacterial cultures during the production of the modified protein IL-33 Y143 azidoPhe (1.6 mg L^{-1}, page 18). We have also included the analysis of gels (e.g., Coomassie-stained and in-gel fluorescence to confirm the purity of IL33 Y143 azidoPhe as well as the subsequent IL-33(**3**) and IL-33(**6**) constructs. This data is presented in the new Figure S16.

10. The mass spectra in figure S20 are only the deconvoluted mass spectra. Inclusion of the full unprocessed LC-traces and ion envelopes, as well as an indiciation which part of the ion envelopes were used for the deconvolution are essential for assessing the purity (for example, by inclusion of a coommassie-stained gel in Figure S16).

Answer: We thank the reviewer for the comment. We have replaced the deconvoluted mass spectra for IL-33 Y143 pcY and IL-33 Y163 pcY with unprocessed mass spectra (new Figure S23). As mentioned above, we have also included a more detailed analysis of the purity for both IL-33(**3**) and IL-33(**6**) constructs with Coomassie-stained and in-gel fluorescence analysis in Figure S16.

11. Page S23 has a 50 mg and a 200 mg/mL, which should maybe be replaced by a microsign.

Answer: Thank you. Amended in the revised manuscript.

Reviewer 2

1. The authors are presenting here a generic platform to site-specifically incorporate small BODIPY tags into cytokine proteins (although really any proteins). The success of the manuscript is a pair of propargylated BODIPYs that can be clicked onto azidoPhe in proteins. These labelled cytokines showed native activity profiles and could be imaged with wash-free fluorescence microscopy. This is a neat story in itself. The authors also provide another story of preparing BODIPY-bearing amino acids, which can be ligated to different tRNA constructs, but are not successfully incorporated by ribosomal synthesis. This story does not fit with the rest of the discussion, and I suggest would be better published separately. I believe there is great value in publishing negative results such as this, and indeed this work can be incorporated into a story that confirms that other BODIPY amino caids of the size of BODIPYFL can be incorporated, for example.

Answer: We thank the reviewer for the comment. As detailed in our answer to reviewer 1, our first version included several results -both positive and negative- describing our exploration around the chemical diversification of BODIPY-UAAs as well as their evaluation for incorporation into proteins via different strategies, including flexizymes and chemical amino acylation. Importantly, some of the presented negative results support our conclusions around the preferential incorporation of small-sized BODIPY structures (e.g., BODIPY-FL) over larger hydrophobic amino acids (Trp-BODIPY). As suggested, we decided to remove entirely from the main text all the results and discussion around chemical derivatization of BODIPYs for flexizyme-mediated ligation and subsequent assays with dFx and eFx. These are now included as a Supplementary Discussion in the Supporting Information.

2. My other suggestion for improving the manuscript is a clearer explanation/ discussion of the definition of fluorogenic here: it is not fluorogenic upon clicking to the protein, but rather in a more lipophilic environment. This should be spelled out more clearly, and the advantages enumerated. What if the protein environment being labelled is particularly hydrophilic?

Answer: We thank the reviewer for the comment. We have included in the revised manuscript a clarification (page 18 of the revised manuscript) on the fluorogenic properties of our IL-33 constructs and their environmental sensitivity (e.g., turn-on emission upon receptor binding).
